# ﻿A taxonomic review of the family Myrmeleontidae Latreille (Neuroptera, Myrmeleontiformia) from the Korean peninsula, highlighting the conservation value of this family

**DOI:** 10.3897/zookeys.1262.163194

**Published:** 2025-12-04

**Authors:** Jiseung Kim, Neung-Ho Ahn, Sora Kim

**Affiliations:** 1 Department of Agricultural Convergence Technology, Jeonbuk National University, Jeonju 54896, Republic of Korea; 2 Laboratory of Insect Phylogenetics and Evolution, Department of Plant Protection and Quarantine, Jeonbuk National University, Jeonju 54896, Republic of Korea; 3 National Institute of Biological Resources, Ministry of Environment, Incheon, Republic of Korea

**Keywords:** Antlion, faunistic study, morphology, Neuropterida, new record, owlfly, South Korea, taxonomy

## Abstract

The family Myrmeleontidae Latreille (Neuroptera: Myrmeleontiformia) is taxonomically reviewed from South Korea. The Myrmeleontidae was a group that had received only limited attention from researchers in Korea, and the most recent taxonomic study of Korean Myrmeleontidae was conducted by Okamoto (1926). In this study, 16 species in 11 genera are identified, with four species (*Distoleon littoralis* Miller & Stange, 1999, *Myrmeleon immanis* Walker, 1853, *Paraglenurus albiventris* Matsumoto, Kikuta & Hayashi, 2021, and *Paraglenurus melanostictus* Matsumoto, Kikuta & Hayashi, 2021) reported for the first time in Korea. A comprehensive re-examination and visual documentation of all Korean species of Myrmeleontidae is conducted. A key to the tribes, genera, and species of Korean antlions is provided. Descriptions and illustrations of ten antlion larvae, including five from the tribe Myrmeleontini, are also provided. Based on the biological information from this study, the current distribution patterns and conservation value of the Myrmeleontidae in South Korea are discussed.

## ﻿Introduction

**Figure 1.**
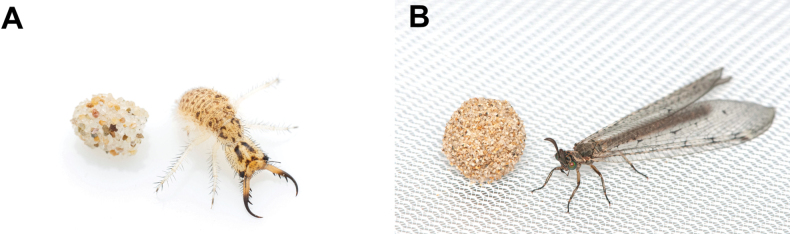
A. Eggshell and larva of *Synclisis japonica*; B. Coccon and adult of *Euroleon coreanus*.

The family Myrmeleontidae Latreille is one of the largest families of the order Neuroptera, encompassing approximately 2,160 described species (Oswald 2025). Machado et al. (2019) placed the family Ascalaphidae as a subfamily of Myrmeleontidae, whichis presently classified into four subfamilies: Ascalaphinae, Myrmeleontinae, Dendroleontinae, and Nemoleontinae. The family is distributed worldwide and is predominantly diverse in arid areas of the tropical and subtropical regions (Stange 2004). Antlions are categorized as holometabolous insects (Fig. 1). Their larvae have diverse habitats and predation strategies, and among them, the ecology of some species that create conical pits in the soil is well documented and has been the subject of numerous studies (Lucas 1989; Mansell 1999; Büsse et al. 2021). Adults are characterized by a forewing length range of 10–75 mm (Oswald and Machado 2018). The adults are skilled predators, but some species known to feed on pollen (Stelzl and Gepp 1990; Hollis et al. 2011; Michel et al. 2017; Oswald and Machado 2018; Marquez-López et al. 2024).

Myrmeleontidae was the subject of only a very limited number of studies in Korea. The most recent taxonomic study of Korean Myrmeleontidae was conducted by Okamoto (1926), describing one new and seven unrecorded species in Korean Peninsula. Almost a century after Okamoto’s research, several taxonomic revisions of Myrmeleontidae have been conducted, and new species have been discovered in neighboring countries (Hayashi et al. 2020, 2024; Matsumoto et al. 2021; Zheng et al. 2022, 2024a). To date, 13 species in 11 genera have been recorded in Korean peninsula (Paek 2010).

This paper constitutes a revision of the Korean species of Myrmeleontidae. Sixteen species, including four unrecorded species, assigned to 11 genera are recognized, accompanied by detailed descriptions and illustrations. A key to the species of the Korean Myrmeleontidae is also provided to facilitate identification. The distribution and biology are provided as fundamental data for the conservation of this group.

## ﻿Materials and methods

**Figure 2.**
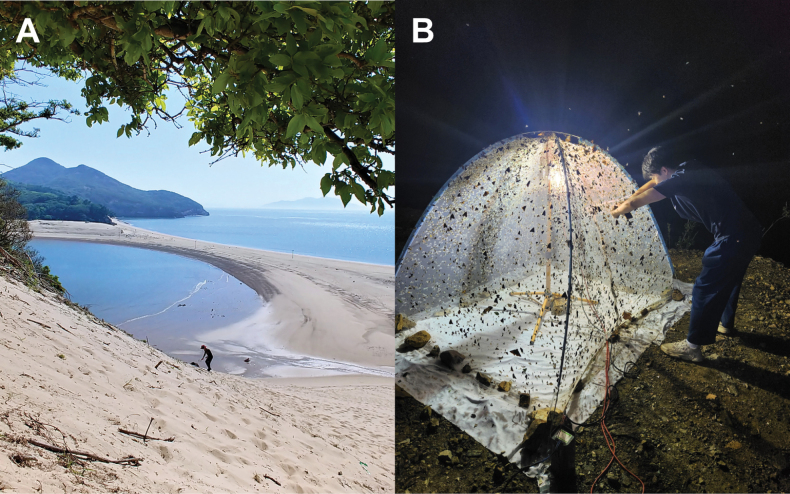
A. Collecting the larvae in the habitat (Ongjin-gun, Incheon); B. Collecting the adults with light trap (Uljin-gun, Gyeongsangbuk-do).

Specimens were obtained from South Korea through two distinct methodologies. Firstly, adults and larvae were collected directly from the field (Fig. 2), and secondly, the field-collected larvae were reared in the laboratory to adulthood. Adult samples were collected using insect nets and light traps. Larval samples were collected using a stainless-steel sand scoop. The larvae for rearing were individually put in plastic vessels, including a sufficient amount of sand to prevent cannibalism. The specimens used in this study were dried or preserved in 80% ethanol. A total of 393 individuals, 343 adults and 50 larvae sampled in 53 sites, were used for morphological observations. All specimens are deposited in the Jeonbuk National University (**JBNU**, Jeonju, South Korea).

Morphological terminology follows Sekimoto (2014), Breitkreuz et al. (2017), Wang et al. (2018), and Machado and Oswald (2020) for adults and Cesaroni et al. (2010) and Badano and Pantaleoni (2014a, 2014b) for larvae. The abbreviations used for wing veins: **1A**, **2A**, **3A** – anal veins; **CuA** – cubitus anterior; **CuP** – cubitus posteroir; **MA** – media anterior; **MP** – media posterior; **RA** – radius anterior; **RP** – radius posterior. The abbreviations used for adult size measurements: **BL** – body length from the front of the head to the tip of the abdomen; **FWL** – forewing length from its base to apex; **HWL** – hindwing length from its base to apex. The abbreviations used for larval size measurements: **BL** – body length from the front of heads, excluding mandibles, to the tip of abdomens; **HL** – head length measured ventrally from the clypeo-labrum to the head insertion with the thorax; **HW** – head width taken at the point of maximum width; **ML** – mandible length from its apex to base. Larval size measurements were taken from the largest individual. Genital preparations were made with 10% KOH at 70 °C for 20 min (Hayashi et al. 2020). After rinsing the KOH with distilled water, the apex of the abdomen was transferred to glycerin for further examination (Zheng et al. 2022).

The specimens were observed using a OM SYSTEM OM-1 Mirrorless camera (OM Digital Solutions, Japan) equipped with a M.ZUIKO DIGITAL ED 90 mm F3.5 Macro IS PRO (OM Digital Solutions, Japan), or a Canon EOS 6D camera (Canon, Japan) with a Canon Macro Lens EF 100 mm (Canon, Japan). Dissected genitalia were observed using a Tucsen Dhyana 400 DC digital camera (Tucsen Photonics, China) with Leica S8AP0 stereomicroscope (Leica Micro systems, Germany). Photographs were stacked using Mosaic software (v. 2.4) or Helicon Focus software (v. 8.2.2. Pro, Helicon Soft, Ukraine) and stacked digital images were taken using Adobe Photoshop 2023 (v. 24.7.5, Adobe, USA). The distribution map was created using QGIS 3.40.4 (OGSeo). The background map data was provided from GEOSERVICE-WEB (GEOSERVICE, Korea).

## ﻿Taxonomic accounts

**Family Myrmeleontidae Latreille, 1802**

**Subfamily Myrmeleontinae Latreille, 1802**

**Tribe Acanthaclisini Navás, 1912**

### ﻿Genus Synclisis

Taxon classificationAnimaliaNeuropteraMyrmeleontidae

Navás, 1919

7654AADE-887B-53FD-9BEA-71ABEA6B9316

Synclisis Navás, 1919a: 218. Type species: Acanthaclisis baetica Rambur, 1842. Type locality: Spain: “environs de Malaga”.

#### Diagnosis.

Adult. Large sized antlions; thorax and legs densely hairy; forewing presectoral area usually with 5–10 crossveins; forewing vein RP arising beyond CuA fork; forewing vein 2A fused with 3A basally; hindwing presectoral area usually with five crossveins; hindwing vein RP arising beyond MP fork; male with pilula axillaris; tibial spurs strongly curved, usually as long as combined lengths of tarsomeres 1–3 (Sekimoto 2014). Third instar larva. Mandibles with three equidistant teeth, the apical tooth is the largest; no setae between the base of the mandible and basal tooth; thorax with sessile setiferous processes; abdominal sternite VIII without digging setae; abdominal sternite IX triangular with a median series of digging setae (Badano and Pantaleoni 2014a).

#### Distribution.

Oriental (Malaysia, Vietnam), Palearctic (Algeria, China, Iran, Israel, Japan, Korea, Russia, Senegal, Tunisia, Ukraine, Southern Europe) (Krivokhatsky 2011; Badano and Pantaleoni 2014a; Wang et al. 2018; Hajiesmaeilian et al. 2019).

### ﻿ Synclisis japonica

Taxon classificationAnimaliaNeuropteraMyrmeleontidae

(Hagen, 1866)

394B60CE-862D-5A2D-844E-42C24E11491A

**Figure 3.**
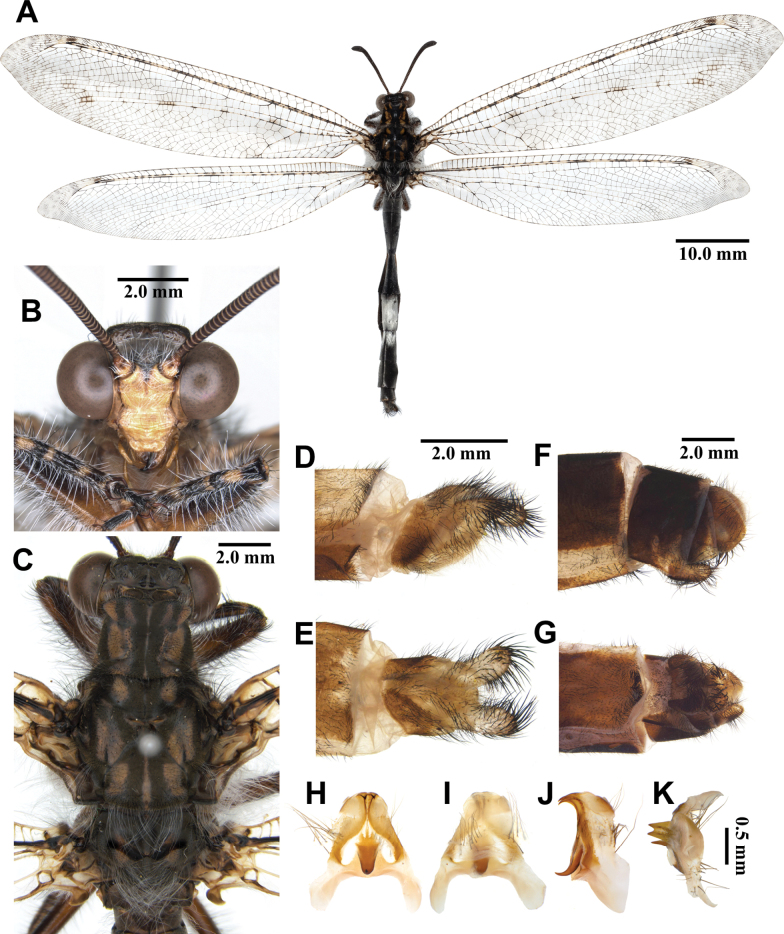
*Synclisis japonica* (Hagen, 1866a), adult. A. Dorsal habitus, male; B. Head, frontal view; C. Head and thorax, dorsal view; D, E. Male terminalia: D. Lateral view; E. Ventral view; F, G. Female terminalia: F. Lateral view; G. Ventral view; H–K. Male genitalia: H. Dorsal view; I. Ventral view; J. Lateral view; K. Caudal view.

**Figure 4.**
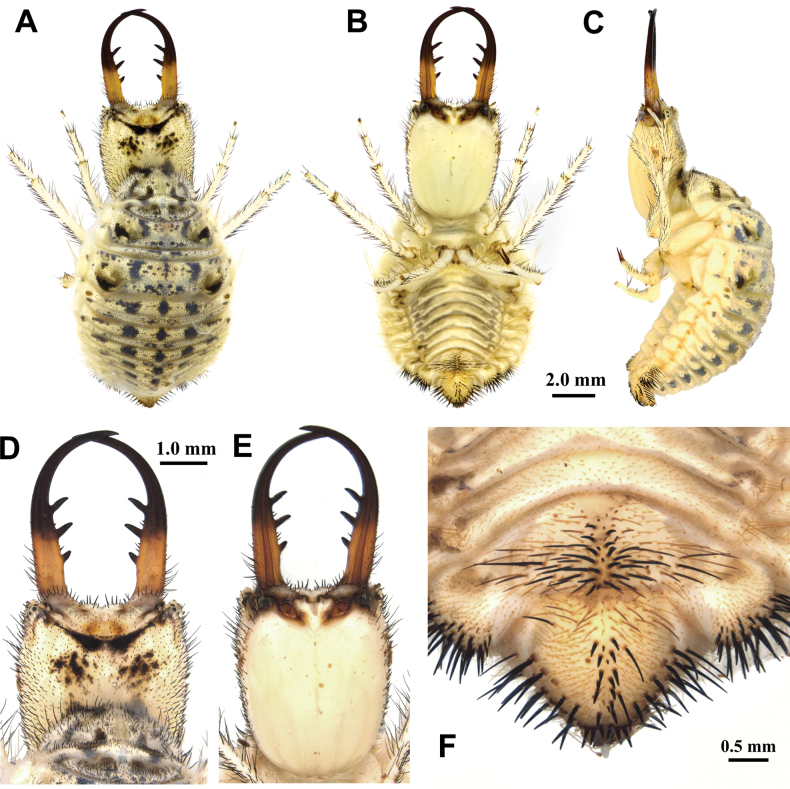
*Synclisis japonica* (Hagen, 1866a), third instar larva. A–C. Habitus: A. Dorsal view; B. Ventral view; C. Lateral view; D, E. Head: D. Dorsal view; E. Ventral view; F. Abdominal sternite IX.

Figs 3, 4, 29A, 31, 35A, 36G, 37A

Acanthaclisis japonica Hagen, 1866a: 289. Type locality: Japan: Tokyo.
Heoclisis japonica (Hagen, 1866a): Navás 1923b: 13.
Heoclisis sinensis Navás, 1923b: 13. Type locality: China: eastern, “Chen-Chia-Tchoueng”.
Synclisis japonica (Hagen, 1866a): Stange 2004: 359.

#### Specimens examined.

[**JBNU**] • 1♂1♀, Oeseonmi-ri, Onjeong-myeon, Uljin-gun, Gyeongsangbuk-do, Korea, 27.VII.2022, J.S. Kim; • 2♂5♀, Seopo-ri, Deokjeok-myeon, Ongjin-gun, Incheon, Korea, 13.VIII.2024, J.S. Kim; • 4♂2♀, Gureom-ri, Deokjeok-myeon, Ongjin-gun, Incheon, Korea, 14.VIII.2024, J.S. Kim; • 1♂, Gilgok-ri, Maehwa-myeon, Uljin-gun, Gyeongsangbuk-do, Korea, 28.VIII.2024, J.S. Kim; • 1♀, same locality, 7.IX.2024, J.S. Kim; • 5 larvae (1^st^ and 3^rd^ instar), Gureom-ri, Deokjeok-myeon, Ongjin-gun, Incheon, Korea, 14.VIII.2024, J.S. Kim.

#### Diagnosis.

*Synclisis japonica* is easily distinguished from the other Korean Myrmeleontidae species by its large body size and long wingspan. Thorax and legs are densely hairy. Tergite V and proximal half of tergite VI are densely covered with appressed shiny silver pubescence in male. In larvae, the orange area at the base of the mandible reaches the second tooth. The center of the clypeo-labrum lacks a median longitudinal black stripe. Abdominal sternite VIII has several large setae.

This species is similar to *Synclisis kawaii* (Nakahara, 1913) from southern China in general appearance. The two species can be distinguished by differences in the Banksian line and the marking pattern of the pronotum. In *S. japonica*, the anterior and posterior Banksian lines on the forewings and hindwings are distinct, while they are indistinct in *S. kawaii*. *Synclisis japonica* has a distinct black stripe on the pronotum, whereas in *S. kawaii*, it is faint (Sekimoto 2014).

#### Description.

**Male, adult. *Head*** (Fig. 3B, C). Vertex slightly narrow, moderately raised, dark brown, densely covered with short black hairs, with sparse long white hairs anteriorly. Frons yellow, densely covered with long white hairs; clypeus yellow, with sparse dark brown hairs. Antenna dark brown, slightly long, with slightly defined club, densely covered with short black hairs; flagellum comprising ~45 flagellomeres, each flagellomere with a narrow distal yellow ring. Mouthparts yellowish brown; labrum yellowish brown, with hyaline brown hairs; maxillary palpus yellowish brown; labial palpus yellowish brown, much longer than maxillary palpus; 3^rd^ labial palpomere reddish brown.

***Thorax*** (Fig. 3C). Pronotum broad, approximately as long as broad, dark brown, with pairs of longitudinal yellowish brown stripes, densely covered with long black and white hairs. Mesonotum dark brown, with pairs of longitudinal yellowish brown stripes, densely covered with long black and white hairs. Metanotum dark brown, with pair of yellow spots in the middle, densely covered with long white hairs.

***Legs*.** Yellowish brown, short. Coxae yellowish brown, densely covered with long white hairs. Femora yellowish brown, dark brown distally, densely covered with long white and black setae. Tibiae alternating yellowish brown and dark brown, densely covered with long white and black setae. Tibial spurs reddish brown, short, strongly curved, approximately as long as combined lengths of tarsomeres 1–3. Tarsi dark brown, tarsomere 5 approximately as long as combined lengths of tarsomeres 1–4. Claws reddish brown.

***Wings*** (Fig. 3A). With dark brown markings. Forewings veins and crossveins alternating pale yellow and dark brown; presectoral area with 8–10 crossveins; RP arising beyond CuA fork; CuP supporting one cell before fusing with 1A; 2A fused with 3A; pterostigma yellowish white; anterior and posterior Banksian lines distinct. Hindwings shorter and narrower than forewings; presectoral area with 6–8 crossveins; RP arising beyond MP fork; pterostigma yellowish white; anterior and posterior Banksian lines distinct; male with pilula axillaris.

***Abdomen*** (Fig. 3A). Shorter than hindwing, grayish black, densely covered with short black hairs, tergite V and proximal half of tergite VI densely covered with shiny silver pubescence.

***Genitalia*** (Fig. 3D, E, H–K). Ectoproct triangular in lateral view, covered with long black setae. Sternite IX elongated, covered with long black setae. Gonarcus brown, triangular, with short lateral arm. Mediuncus well sclerotized, reddish brown, strongly hooked in lateral view. Parameres well sclerotized, dark brown, strongly hooked in lateral view.

Size. BL: 43.0–49.7 mm; FWL: 50.7–54.8 mm; HWL: 45.1–49.6 mm.

**Female, adult.** Except terminalia, generally similar to male. Pilula axillaris absent. Shiny silver pubescence of tergite V and proximal half of tergite VI absent (Fig. 26). Terminalia (Fig. 3F, G): tergite VIII much wider than tergite IX; tergite IX narrow, triangular in lateral view; ectoproct semicircular in lateral view; lateral gonapophyses small, with long black setae; posterior gonapophyses long, curved, with long black setae; anterior gonapophyses absent; pregenital plate absent.

Size. BL: 39.5–45.4 mm; FWL: 49.6–58.5 mm; HWL: 43.7–52.9 mm.

**Larva, 3^rd^ instar.** General color yellowish white, with black markings (Fig. 4A–C). Head rectangular, longer than wide, with a pair of large black markings, lateral and ventral sides unmarked; mandibles pale orange with a dark apex; interdental mandibular setae (0) (1–3) (1–3) (0); external setae short, restricted in proximal part (Fig. 4D, E). Abdominal sternite VIII with black digging setae, thicker in proximity of the distal margin; abdominal sternite IX triangular, with black digging setae, caudal margin with large black setae (Fig. 4F).

Size. BL: 18.9 mm; HL: 5.7 mm, HW: 4.2 mm, ML: 5.1 mm.

#### Biological notes.

*Synclisis japonica* occurs in areas with developed natural dunes along the west and east coasts of South Korea, excluding the south coast (Fig. 37A) where adults mainly emerge from late July to early September. Larvae are mainly observed in well-preserved coastal dunes where *Carex* (Cyperaceae) grows (Fig. 36G). They are ambush predators, concealing themselves in the sand of dunes. When they detect prey, they rapidly emerge from the sand and crawl forward to hunt. After capturing their prey, they crawl backward to burrow back into the sand, often leaving only the prey exposed on the dune’s surface. Larvae of various development stages are observed at the same time. Eggs were observed in same place where larvae were collected.

#### Distribution.

Korea, Japan, China, Russia (Krivokhatsky 2011; Sekimoto 2014; Wang et al. 2018).

#### Remarks.

*Synclisis japonica* is the largest antlion species in Korea, first reported from Korea by Okamoto (1926).

### ﻿Tribe Myrmeleontini Latreille, 1802

#### ﻿Genus Baliga

Taxon classificationAnimaliaNeuropteraMyrmeleontidae

Navás, 1912

7AA345C6-9621-56C5-BAA4-0BB055ABCE1A

Baliga Navás, 1912a: 110. Type species: Myrmeleon asakurae Okamoto, 1910. Type locality: Taiwan: Horisha.
Balaga Navás, 1912a: 110.
Baga Navás, 1930a: 37.

##### Diagnosis.

Adult. Medium to large sized antlions; wings without marking; forewing presectroal area usually with 5–10 crossveins; forewing vein 2A fused with 3A; forewing vein RP arising opposite or slightly beyond CuA fork; hindwing presectoral area usually with five crossveins; hindwing vein RP arising opposite or slightly beyond MP fork; male usually with pilula axillaris; tibial spurs approximately as long as tarsomere 1 (Sekimoto 2014). Third instar larva. Head and mandibles elongated; abdominal sternite IX elongated, without or with few reduced digging setae; hind femur with a dark brown spot (Hayashi et al. 2020; Lin et al. 2021).

##### Distribution.

Australia (Queensland), Oriental (Bangladesh, India, Myanmar, Sri Lanka, Vietnam, Indonesia, Malaysia, Micronesia, Philippines), Palearctic (China, Japan, Korea) (Hassan et al. 2022).

#### ﻿ Baliga micans

Taxon classificationAnimaliaNeuropteraMyrmeleontidae

(McLachlan, 1875)

BFA8529C-DE2D-5568-A445-D7DC53645F2E

**Figure 5.**
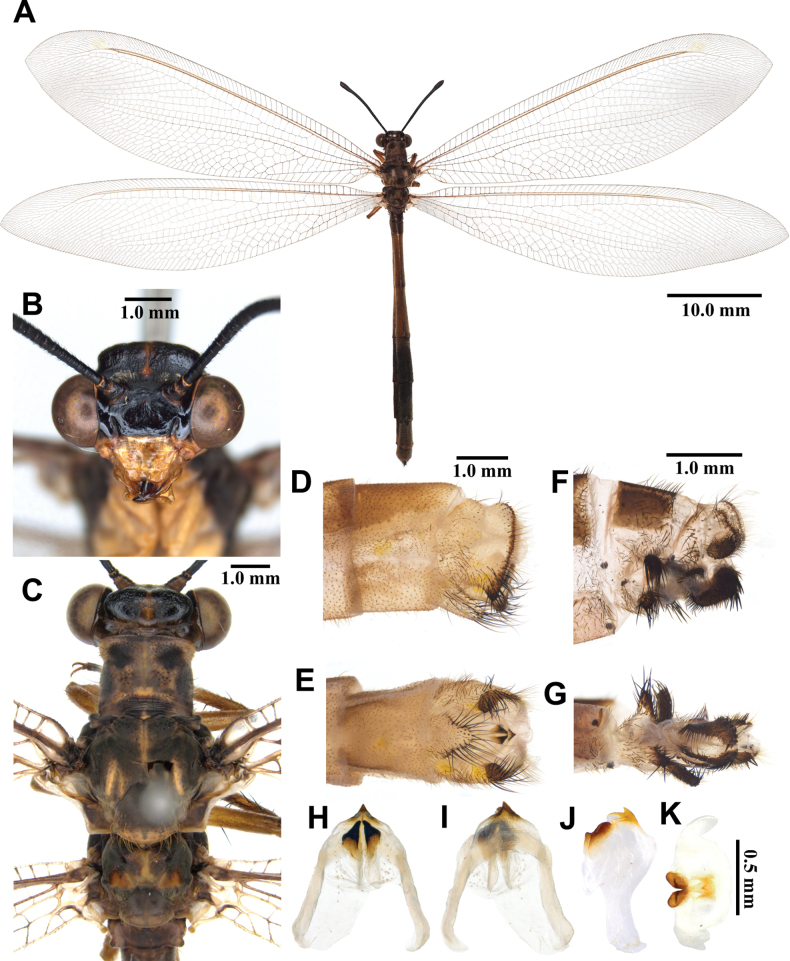
*Baliga micans* (McLachlan, 1875), adult. A. Dorsal habitus, male; B. Head, frontal view; C. Head and thorax, dorsal view; D, E. Male terminalia: D. Lateral view; E. Ventral view; F, G. Female terminalia: F. Lateral view; G. Ventral view; H–K. Male genitalia: H. Dorsal view; I. Ventral view; J. Lateral view; K. Caudal view.

**Figure 6.**
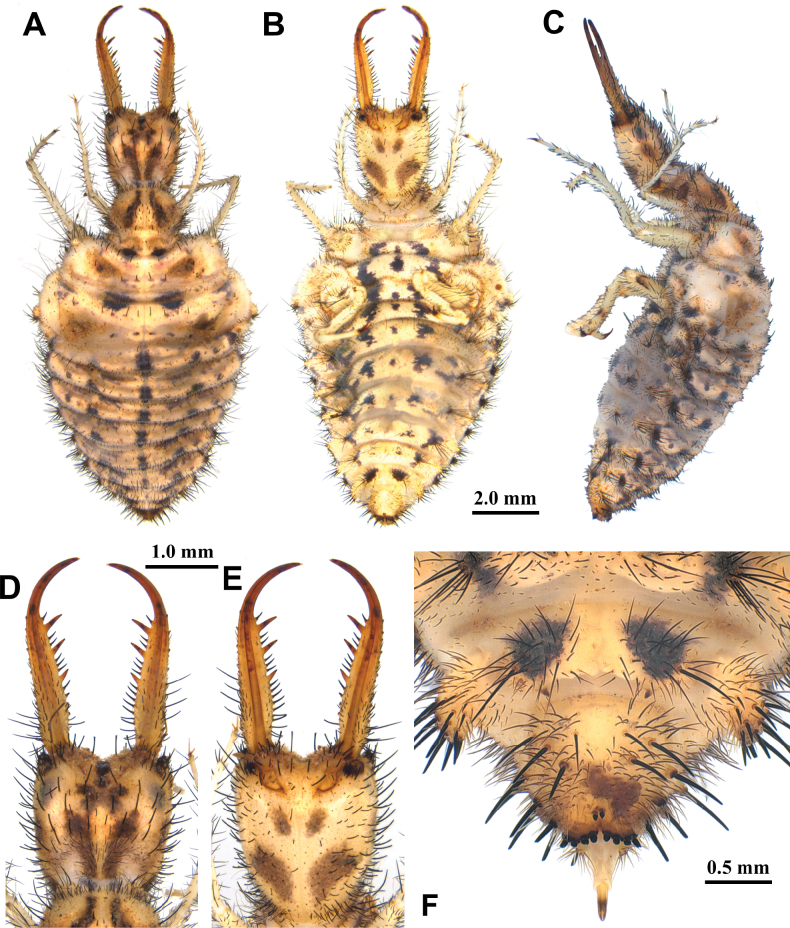
*Baliga micans* (McLachlan, 1875), third instar larva. A–C. Habitus: A. Dorsal view; B. Ventral view; C. Lateral view; D, E. Head: D. Dorsal view; E. Ventral view; F. Abdominal sternite IX.

Figs 5, 6, 35B, 36A, 37A

Myrmeleon micans McLachlan, 1875: 176. Type locality: Japan: Yokohama.
Balaga micans (McLachlan, 1875): Navás 1912a: 111.
Hagenomyia micans (McLachlan, 1875): Okamoto 1914: 250.
Baliga micans (McLachlan, 1875): Stange 2004: 297.

##### Specimens examined.

[**JBNU**] • 1♀, Oeseonmi-ri, Onjeong-myeon, Uljin-gun, Gyeongsangbuk-do, Korea, 27.VII.2022, J.S. Kim; • 1♂, Oegok-ri, Toji-myeon, Gurye-gun, Jeollanam-do, Korea, 2.VII.2023, J.S. Kim; • 2♂, Samjung-ri, Macheon-myeon, Hamyang-gun, Gyeongsangnam-do, Korea, 14.VII.2023, H. Han; • 1♀, Gilgok-ri, Maehwa-myeon, Uljin-gun, Gyeongsangbuk-do, Korea, 20.VII.2023, DB Choi; • 1♂, Yulji-ri, Susan-myeon, Jecheon-si, Chungcheongbuk-do, 30.VII.2024, J.S. Kim; • 1♂, Sindu-ri, Wonbuk-myeon, Taean-gun, Chungcheongnam-do, 31.VII.2024, J.S. Kim; • 4♂2♀, Gureom-ri, Deokjeok-myeon, Ongjin-gun, Incheon, Korea, 15.VIII.2024, J.S. Kim; • 1♀, Buchun-ri, Hwagae-myeon, Hadong-gun, Gyeongsangnam-do, Korea, 27.VIII.2024, J.S. Kim; • 1♂1♀, Gilgok-ri, Maehwa-myeon, Uljin-gun, Gyeongsangbuk-do, Korea, 7.IX.2024, J.S. Kim; 1 larva (3^rd^ instar), Yongdu-dong, Buk-gu, Gwangju, Korea, 2.VII.2023, J.S. Kim; 1 larva (3^rd^ instar), Nogok-ri, Bongsan-myeon, Hapcheon-gun, Gyeongsangnam-do, Korea; 28.VI.2024, J.S. Kim; 1 larva (2^nd^ instar), Namseo-ri, Seo-myeon, Ulleung-gun, Gyeongsangbuk-do, Korea, 11.VII.2024, J.S. Kim; • 4 larvae (3^rd^ instar), Hakbong-ri, Banpo-myeon, Gongju-si, Chungcheongnam-do, Korea, 1.X.2024, J.S. Kim; • 3 larvae (2^nd^ instar), Deokjin-dong, Deokjin-gu, Jeonji-si, Jeonbuk-do, Korea, 4.X.2024, J.S. Kim; • 2 larvae (3^rd^ instar), Changwon-ri, Nam-myeon, Yeongwol-gun, Gangwon-do, Korea, 24.V.2025, J.S. Kim.

##### Diagnosis.

*Baliga micans* is similar to *B. ryukuensis* Hayashi & Matsumoto, 2020 from southern Japan (Amami Island, Tokunoshima Island, Okinawa Island) in general appearance (Hayashi et al. 2020). Compared to *B. micans*, the male of *B. ryukyuensis* has a larger sternite IX, an un-sclerotized dorsolateral edge, and more separated and sclerotized parameres. In females, *B. ryukyuensis* differs by having slightly shorter lateral gonapophyses (Hayashi et al. 2020). It is easily distinguished from *B. asakurae* (Okamoto, 1910) and *B. kimurai* Hayashi, 2020 by the size of the pterostigma and the color pattern on the pronotum (Hayashi et al. 2020). In larvae, mandibles have three equidistant teeth with the apical tooth slightly stronger. Abdominal sternite IX is elongated and has 0–2 short digging setae in front of rastra.

##### Description.

**Male, adult. *Head*** (Fig. 5B, C). Vertex wide, strongly raised, black, with short black hairs. Frons black; clypeus yellow. Antenna black, slightly long, with slightly defined club, densely covered with short black hairs; flagellum comprising ~38 flagellomeres. Mouthparts yellowish brown; labrum yellowish brown, with hyaline brown hairs; maxillary palpus yellowish brown, 5^th^ maxillary palpomere dark brown; labial palpus yellowish brown, 3^rd^ labial palpomere tapering to acute apex.

***Thorax*** (Fig. 5C). Pronotum broad, approximately as long as broad, dark brown, anterior margin yellow, with yellow longitudinal midline, moderately covered with black hairs. Mesonotum dark brown, with sparse yellow and black hairs. Metanotum dark brown, with pair of yellow spots at the middle, with sparse yellow hairs.

***Legs*.** Yellow, short. Coxae yellow, moderately covered with long brown hairs. Femora yellowish brown, moderately covered with black setae. Tibiae yellowish brown, moderately covered with black setae. Tibial spurs reddish brown, short, almost straight, approximately as long as combined lengths of tarsomeres 1–3. Tarsi dark brown, tarsomere 5 approximately as long as combined lengths of tarsomeres 1–4; claws reddish brown.

***Wings*** (Fig. 5A). Without markings. Forewings veins and crossveins mostly brown; presectoral area with 6–8 crossveins; RP arising opposite or slightly beyond CuA fork; CuP supporting one or two cells before fusing with 1A; 3A fused with 2A; pterostigma yellowish white; anterior Banksian line absent, posterior Banksian line distinct. Hindwings approximately as long as forewings, narrower than forewings; presectoral area with 4–6 crossveins; RP arising opposite or slightly beyond MP fork; pterostigma yellowish white; anterior Banksian line absent, posterior Banksian line distinct; male with pilula axillaris.

***Abdomen*** (Fig. 5A). Shorter than hindwing, dark brown, densely covered with brown hairs.

***Genitalia*** (Fig. 5D, E, H–K). Ectoproct rectangular covered with long black setae. Sternite IX narrow, covered with long black setae. Gonarcus yellowish white, arched, with long lateral arms. Mediuncus well sclerotized, dark brown, moderately hooked in lateral view. Parameres well sclerotized, dark brown, triangular in ventral view.

Size. BL: 34.5–36.5 mm; FWL: 39.9–44.4 mm; HWL: 40.3–44.9 mm.

**Female, adult.** Except terminalia, generally similar to male. Pilula axillaris absent. Terminalia (Fig. 5F, G): tergite VIII wider than tergite IX; tergite IX narrow, oval in lateral view; ectoproct semicircular in lateral view; lateral gonapophyses rectangular in lateral view, slightly bigger than ectoproct, with long black setae; posterior gonapophyses long, slender, with long black setae; anterior gonapophyses long, with long black setae; pregenital plate distinct, semicircular, presented on posterior margin of sternite VII.

Size. BL: 34.5–36.5 mm; FWL: 39.9–44.4 mm; HWL: 40.3–44.9 mm.

**Larva, 3^rd^ instar.** General color yellowish brown, with dark brown markings (Fig. 6A–C) . Head longer than wide, with an anterior pair of spots and a V-shaped dark brown marking on dorsal side, with two pairs of dark brown spots on ventral side, with three dark brown spots in lateral side; mandibles yellowish brown; interdental mandibular setae (6–10) (2–3) (2–3) (1); external setae long. Abdominal sternite VIII with sparse black setae (Fig. 6D, E). Abdominal sternite IX with 0–2 short digging setae in front of rastra; a paired rastra each with three or four digging setae (Fig. 6F).

Size. BL: 11.8 mm; HL: 2.6 mm; HW: 2.1 mm; ML: 3.4 mm.

##### Biological notes.

*Baliga micans* is a species that is commonly observed and is distributed throughout South Korea, including Ulleungdo Island (Fig. 37A). This species is primarily observed in areas characterized by mountainous topography but also collected in urban forests and in the vicinity of residential areas. Adults emerge from July to September in South Korea. Larvae are pit builders. They were collected in fine soil under tree bases, rocks, and artificial structures that are protected from rainfall (Fig. 36A).

##### Distribution.

Korea, Japan, China, Vietnam, India, Indonesia, Malaysia, Papua New Guinea, the Philippines (Stange 2004; Sekimoto 2014; Hayashi et al. 2020).

##### Remarks.

There are two views between the genera *Baliga* Navás and *Hagenomyia* Banks. According to the first opinion, *Baliga* is treated as the synonym of *Hagenomiya* (Esben-Petersen 1913; Markl 1954; Wang et al. 2018). Conversely, the opposing view treats both *Baliga* and *Hagenomyia* as valid genera (Stange 2004; Hayashi et al. 2020). In this study, we concur with the latter perspective but the relationship between the genera *Baliga* and *Hagenomyia* requires further study (Wang et al. 2018; Lin et al. 2024).

#### ﻿Genus Euroleon

Taxon classificationAnimaliaNeuropteraMyrmeleontidae

Esben-Petersen, 1918

F9983F11-1B5D-59F1-8A17-3BD712647558

Euroleon Esben-Petersen, 1918a: 125. Type species: Myrmeleon europaeus McLachlan, 1873.
Teula Navás, 1930b: 5.

##### Diagnosis.

Adult. Medium sized antlions; forewing vein CuA2 and CuP+1A generally parallel; forewing vein RP arising beyond CuA fork; forewing vein 2A fused with 3A; forewing anterior Banksian lines absent; forewing posterior Banksian lines distinct; hindwing vein RP arising beyond MP fork; hindwing presectoral area with four or more crossveins; male with pilula axillaris (Wang et al. 2018). Third instar larva. Mandibles with three equidistant teeth with the apical tooth slightly longer; external margin of the mandible with long setae; abdominal sternite VIII with odontoid processes; abdominal sternite IX with an anterior row of digging setae and two short rastra (Badano and Pantaleoni 2014a).

##### Distribution.

Palearctic (widely distributed) (Badano and Pantaleoni 2014a).

#### ﻿ Euroleon coreanus

Taxon classificationAnimaliaNeuropteraMyrmeleontidae

Okamoto, 1926

E7F7B732-9839-564D-992F-7EB21F369C6C

**Figure 7.**
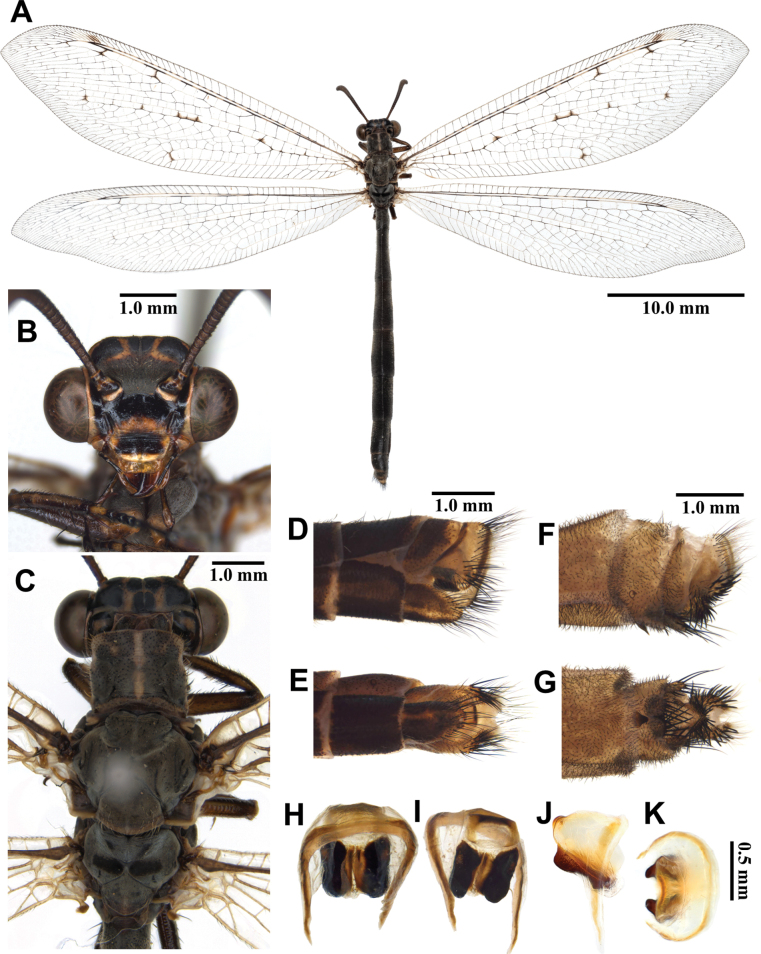
*Euroleon coreanus* Okamoto, 1926, adult. A. Dorsal habitus, male; B. Head, frontal view; C. Head and thorax, dorsal view; D, E. Male terminalia: D. Lateral view; E. Ventral view; F, G. Female terminalia: F. Lateral view; G. Ventral view; H–K. Male genitalia: H. Dorsal view; I. Ventral view; J. Lateral view; K. Caudal view.

**Figure 8.**
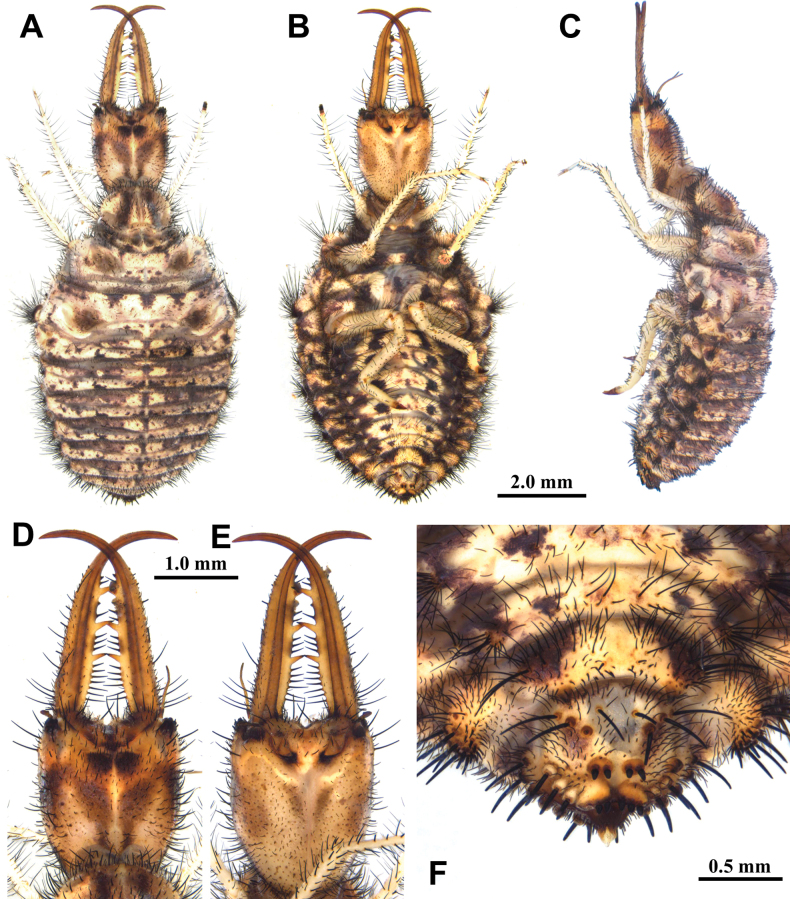
*Euroleon coreanus* Okamoto, 1926, third instar larva. A–C. Habitus: A. Dorsal view; B. Ventral view; C. Lateral view; D, E. Head: D. Dorsal view; E. Ventral view; F. Abdominal sternite IX.

Figs 7, 8, 32B, 35C, 36B, 37F

Euroleon coreanus Okamoto, 1926: 19. Type locality: Korea.
Euroleon sjostedti Navás, 1928: 30. Type locality: China: Gansu: Sanchowfu.
Teula sinica Navás, 1930b: 6. Type locality. China: Hebei: Chengde.
Euroloen alienus Navás, 1932: 111. Type locality: China: Shaanxi: Tapaischan: Tsinling-schan mont.
Euroleon sinuosus Navás, 1935: 42. Type locality: China: Shaanxi: Tapaischan: Tsinling-schan mont.
Euroleon sinicus (Navás, 1930b): Hölzel 1970a: 128.
Euroleon sanxianus Yang, 1997: 618. Type locality: China: Hubei: Yichang: Zigui: Jiulingtou.
Euroleon flavicorpus Wang, 2009: 53. Type locality: China: Shanxi: Jincheng: Yangcheng.

##### Specimens examined.

[**JBNU**] • 1♂3♀ (reared from larva), Yulji-ri, Susan-myeon, Jecheon-si, Chungcheongbuk-do, Korea, 15.VI.2023, J.I. Shim; • 4♂4♀ (reared from larva), Changwon-ri, Nam-myeon, Yeongwol-gun, Gangwon-do, Korea, 25.VI.2023, J.S. Kim; • 6♂, Yulji-ri, Susan-myeon, Jecheon-si, Chungcheongbuk-do, Korea, 30.VII.2024, J.S. Kim; • 2 larvae (3^rd^ instar), Changwon-ri, Nam-myeon, Yeongwol-gun, Gangwon-do, Korea, 25.VI.2023, J.S. Kim; 1 larva (3^rd^ instar), Changwon-ri, Nam-myeon, Yeongwol-gun, Gangwon-do, Korea, 18.V.2024, J.S. Kim.

##### Diagnosis.

Compared to other species in the genus *Euroleon*, *E. coreanus* has the morphological characteristics of a dark brown thorax and abdomen, a pair of brownish spots and a middle stripe on the pronotum, few dark brown markings on the wings, a small axillary plate, and a black marking on the clypeus. In larvae, the hind coxae are unmarked, abdominal sternite IX has an anterior row of four digging setae, a paired rastra each with three digging setae.

##### Description.

**Male, adult. *Head*** (Fig. 7B, C). Vertex wide, strongly raised, dark brown. Frons Yellow, broad dark brown band extending from below vertex to below base of antenna, with short yellow hairs; clypeus yellow, with black marking at middle. Antenna dark brown, short, with slightly defined club, densely covered with short black hairs; flagellum comprising ~30 flagellomeres. Mouthparts reddish brown; labrum yellow, with hyaline black hairs; maxillary palpus dark brown; labial palpus dark brown, much longer than maxillary palpus, spindle-shaped.

***Thorax*** (Fig. 7C). Pronotum broad, length shorter than width, dark brown, yellow longitudinal midline, with long black hairs. Mesonotum dark brown, darker anteriorly, with long yellow hairs. Metanotum dark brown, with pair of yellow marking in the middle, with yellow hairs.

***Legs*.** Coxae yellow, moderately covered with long brown hairs. Femora yellowish brown, moderately covered with black setae. Tibiae yellowish brown, moderately covered with black setae. Tibial spurs reddish brown, short, almost straight, approximately as long as combined lengths of tarsomeres 1–3. Tarsi reddish brown, tarsomere 5 approximately as long as combined lengtsh of tarsomeres 1–4; claws reddish brown.

***Wings*** (Fig. 7A). With dark brown markings. Forewings veins and crossveins mostly brown; presectoral area with seven or eight crossveins; RP arising beyond CuA fork; CuP supporting one cell before fusing with 1A; 3A fused with 2A; pterostigma white; anterior Banksian line absent, posterior Banksian line distinct. Hindwings approximately as long as forewings; narrower than forewing; presectoral area with 3–5 crossveins; RP arising beyond MP fork; pterostigma white; anterior Banksian line indistinct, posterior Banksian line distinct; male with pilula axillaris.

***Abdomen*** (Fig. 7A). Shorter than hindwing, dark brown, densely covered with brown hairs.

***Genitalia*** (Fig. 7D, E, H–K). Ectoproct rectangular, covered with long black setae. Sternite IX narrow, covered with long black setae. Gonarcus brown, arched, with long lateral arms. Mediuncus lightly sclerotized, dark brown, moderately raised. Parameres well sclerotized, dark brown, rectangular in ventral view.

Size. BL: 24.3–28.6 mm; FWL: 26.8–29.5 mm; HWL: 25.0–27.6 mm.

**Female, adult.** Except terminalia, generally similar to male. Pilula axillaris absent. Terminalia (Fig. 7F, G): tergite VIII wider than tergite IX; tergite IX narrow, triangular in lateral view; ectoproct semicircular in lateral view; lateral gonapophyses semicircular in lateral view; smaller than ectoproct; posterior gonapophyses short, with long black setae; anterior gonapophyses small, with long black setae; pregenital plate distinct, triangular, presented on membrane below tergite VIII.

Size. BL: 24.8–25.9 mm; FWL: 27.4–31.2 mm; HWL: 25.6–29.8 mm.

**Larva, 3^rd^ instar.** General color pale brown, with dark brown markings (Fig. 8A–C). Head longer than wide, with an anterior pair of spots and a V-shaped dark brown marking on dorsal side, with two pairs of dark brown spots on ventral side; with a pair of spots in lateral side; mandibles reddish brown; interdental mandibular setae (6–7) (3) (2–3) (2); external setae long (Fig. 8D, E). Abdominal sternite VIII with long black setae. Abdominal sternite IX with an anterior row of four digging setae, a paired rastra each with three digging setae (Fig. 8F).

Size. BL: 8.9 mm; HL: 2.3 mm, HW: 1.6 mm, ML: 2.6 mm.

##### Biological notes.

*Euroleon coreanus* is observed very locally in calcareous grasslands of Chungcheongbuk-do and Gangwon-do in South Korea (Fig. 37F). Adults emerge from late June in South Korea. Larvae are pit builders. They were collected from dry, fine soils in calcareous grasslands with open surroundings (Fig. 36B).

##### Distribution.

Korea, China, Russia, Kazakhstan, Mongolia (Bao and Wang 2006).

##### Remarks.

*Euroleon coreanus* was recorded as new species by Okamoto (1926) based on specimens from Korea. In contrast, Okamoto (1926) described *E. coreanus* seems to be common in Korea, whereas we affirm that this species is distributed very locally in calcareous grasslands in Chungcheongbuk-do and Gangwon-do.

#### ﻿Genus Myrmeleon

Taxon classificationAnimaliaNeuropteraMyrmeleontidae

Linnaeus, 1767

CDDAE2C4-7080-5E16-A4B1-44F0D319AEBC

Myrmeleon Linnaeus, 1767: 913. Type species: Myrmeleon formicarius Linnaeus, 1767. Type locality: Europe.
Macroleon Banks, 1909: 4.
Enza Navás, 1912a: 113.
Myrmeleodes Navás, 1912b: 242.
Moreyus Navás, 1914c: 55.
Morter Navás, 1915a: 466.
Neleon Navás, 1915b: 53.
Neseurus Navás, 1916: 53.
Myrmeleonellus Esben-Petersen, 1918b: 17.
Leptoleon Esben-Petersen, 1918b: 18.
Cocius Navás, 1919b: 296.
Dicholeon Navás, 1920: 193.
Tafanerus Navás, 1921: 62.
Talosus Navás, 1923a: 35.
Banya Navás, 1923c: 145.
Grocus Navás, 1925: 185.
Colinus Navás, 1925: 187.
Afroleon Navás, 1927a: 13.
Neurocolinus Navás, 1930c: 42.
Nemeyus Navás, 1934a: 502.
Nezuela Navás, 1934b: 155.
Bordus Navás, 1936a: 165.
Congoleon Navás, 1936a: 337.
Hypsoleon Navás, 1936b: 103.
Nelneja Navás, 1936c: 104.

##### Diagnosis.

Adult. Medium to large sized antlions; wing without marking; forewing presectoral area with ~5–10 crossveins; forewing vein RP arising opposite or beyond CuA fork; forewing vein 2A fused with 3A; hindwing presectoral area usually with five crossveins; hindwing vein RP arising opposite or beyond MP fork; male usually with pilula axillaris; tibial spurs approximately as long as Ta1 (Sekimoto 2014). Third instar larva. Mandibles with three equidistant teeth with the apical tooth slightly longer; external margin of the mandibles provided with long setae; labial palpi normally four-articulate; abdominal sternite VIII provided with odontoid processes; abdominal sternite IX at least with an anterior row group of digging setae and two short rastra each with four digging setae, some species with additional digging setae (Badano and Pantaleoni 2014a).

##### Distribution.

Cosmopolitan (Badano and Pantaleoni 2014a).

#### ﻿ Myrmeleon bore

Taxon classificationAnimaliaNeuropteraMyrmeleontidae

(Tjeder, 1941)

E818B5F5-8C22-5998-8FDD-D2969000983D

**Figure 9.**
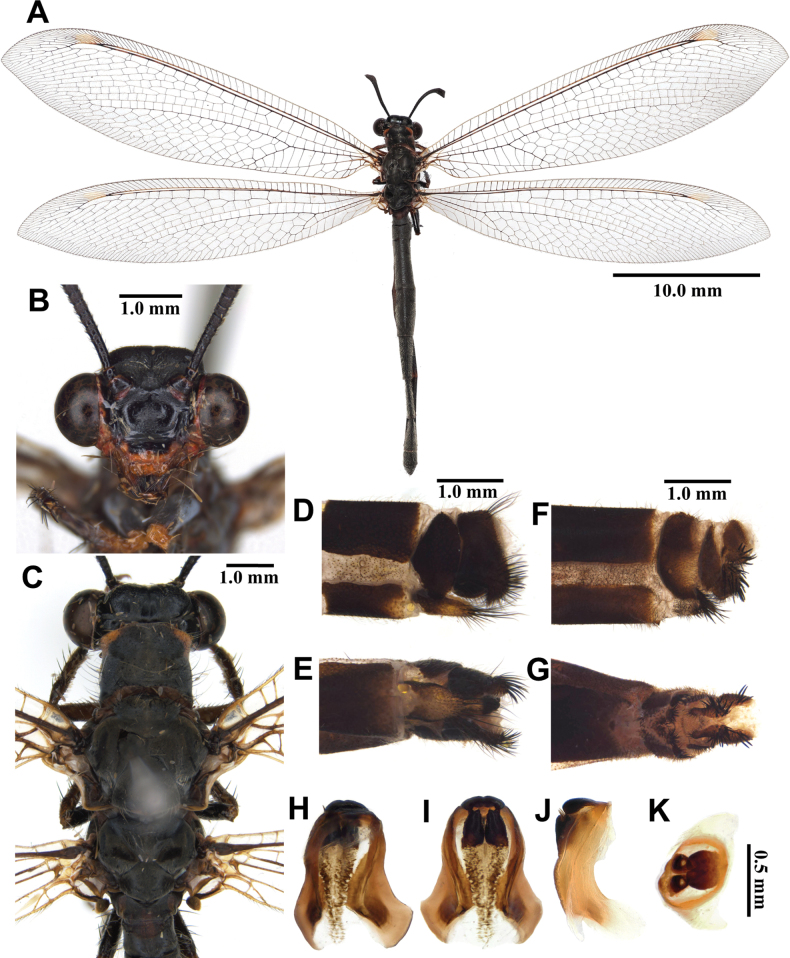
*Myrmeleon bore* Tjeder, 1941, adult. A. Dorsal habitus, male; B. Head, frontal view; C. Head and thorax, dorsal view; D, E. Male terminalia: D. Lateral view; E. Ventral view; F, G. Female terminalia: F. Lateral view; G. Ventral view; H–K. Male genitalia: H. Dorsal view; I. Ventral view; J. Lateral view; K. Caudal view.

**Figure 10.**
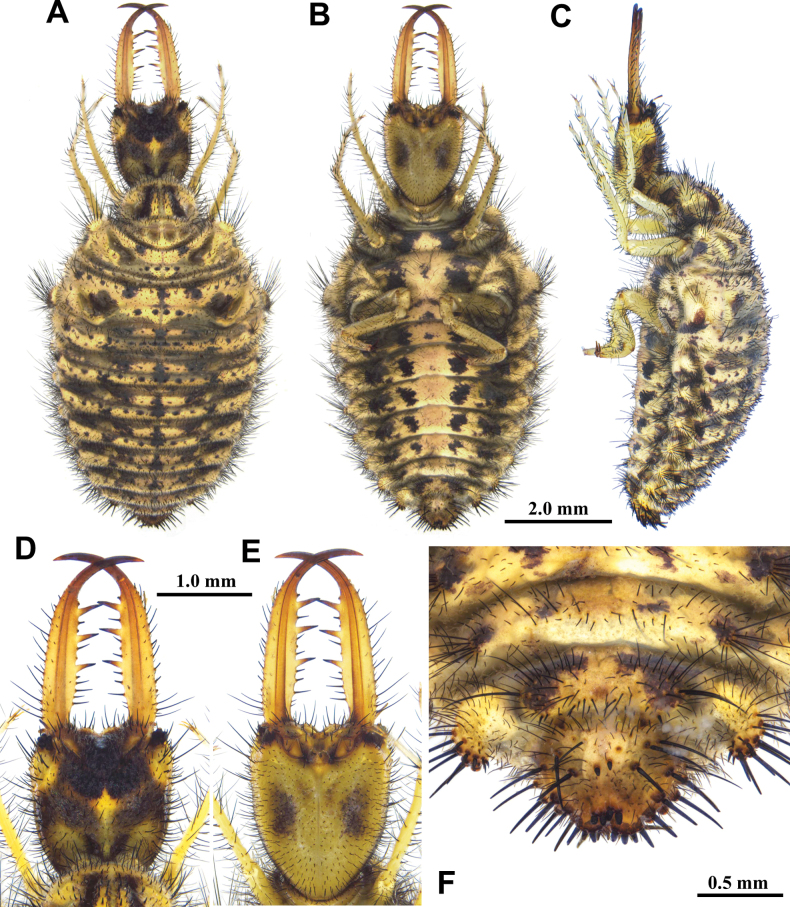
*Myrmeleon bore* Tjeder, 1941, third instar larva. A–C. Habitus: A. Dorsal view; B. Ventral view; C. Lateral view; D–E. Head: D. Dorsal view; E. Ventral view; F. Abdominal sternite IX.

Figs 9, 10, 35D, 36C, 37B

Grocus bore Tjeder, 1941: 74. Type locality: Sweden: Kalmar: Öland: Byrum.
Enza otiosus Navás, 1912: 114. Type locality: Japan.
Myrmeleon bore (Tjeder, 1941): Meinander 1962: 71.
Morter bore (Tjeder, 1941): Friheden 1973: 32.
Myrmeleon exigus Yang, 1999: 148. Type locality: China: Fujian: Dongshan.
Myrmeleon tschernovi Krivokhatsky, Shapoval & Shapoval, 2014: 173. Type locality: Russia: Kaliningrad: Curonian Spit: field station “Fringilla”.

##### Specimens examined.

[**JBNU**] • 1♂, Oeseonmi-ri, Onjeong-myeon, Uljin-gun, Gyeongsangbuk-do, Korea, 9.VII.2022, J.S. Kim; • 4♀, Sindu-ri, Wonbuk-myeon, Taean-gun, Chungcheongnam-do, 20.VIII.2022, J.S. Kim; • 1♀, Sindu-ri, Wonbuk-myeon, Taean-gun, Chungcheongnam-do, 17.VI.2023, J.S. Kim; • 1♂ (reared from larva), Sindu-ri, Wonbuk-myeon, Taean-gun, Chungcheongnam-do, 18.VI.2023, J.S. Kim; • 1♀, Yulji-ri, Susan-myeon, Jecheon-si, Chungcheongbuk-do, Korea, 24.VI.2023, J.S. Kim; • 2♂1♀, Oeseonmi-ri, Onjeong-myeon, Uljin-gun, Gyeongsangbuk-do, Korea, 13.VII.2024, J.S. Kim; • 1♂, Naewol-ri, Bigeum-myeon, Sinan-gun, 26.VII.2024, M.K. Jeong; ; • 1♂, Sindu-ri, Wonbuk-myeon, Taean-gun, Chungcheongnam-do, 31.VII.2024, J.S. Kim; • 1♂, Gorangpo-ri, Jangnam-myeon, Yeoncheon-gun, Gyeonggi-do, 9.VIII.2024, Y.T. Jang; • 1♀, Samgot-ri, Jung-myeon, Yeoncheon-gun, Gyeonggi-do, 10.VIII.2024, Y.T. Jang; • 1♂3♀, Seopo-ri, Deokjeok-myeon, Ongjin-gun, Incheon, Korea, 13.VIII.2024, J.S. Kim; • 1♂1♀, Gureom-ri, Deokjeok-myeon, Ongjin-gun, Incheon, Korea, 14.VIII.2024, J.S. Kim; • 2♂4♀, Gureom-ri, Deokjeok-myeon, Ongjin-gun, Incheon, Korea, 15.VIII.2024, J.S. Kim; • 2♂, Gilgok-ri, Maehwa-myeon, Uljin-gun, Gyeongsangbuk-do, Korea, 7.IX.2024, J.S. Kim; • 3 larvae (3^rd^ instar), Sindu-ri, Wonbuk-myeon, Taean-gun, Chungcheongnam-do, 18.VI.2023, J.S. Kim; • 2 larvae (3^rd^ instar), Samgeum-ri, Geumgangsong-myeon, Uljin-gun, Gyeongsangbuk-do, Korea, 5.VI.2024, J.S. Kim; • 2 larvae (3^rd^ instar), Seopo-ri, Deokjeok-myeon, Ongjin-gun, Incheon, Korea, 13.VIII.2024, J.S. Kim; 1 larva (3^rd^ instar), Gureom-ri, Deokjeok-myeon, Ongjin-gun, Incheon, Korea, 15.VIII.2024, J.S. Kim; • 2 larvae (2^nd^ and 3^rd^ instar), Goeok-ri, Yongjin-eup, Wanju-gun, Jeonbuk-do, Korea, 8.III.2025, Y.T. Jang.

##### Diagnosis.

*Myrmeleon bore* is similar to *M. formicarius* in general appearance. The two species can be distinguished by differences in the Banksian line, the pilula axillaris, and the larval hind coxa. In *M. bore*, the posterior Banksian lines in the forewings are distinct, while they are indistinct in *M. formicarius*. *Myrmeleon bore* has a pilula axillaris in males, whereas it is absent in *M. formicarius*. Unlike the larvae of *M. formicarius*, which have some dark markings on the hind coxa, the larvae of *M. bore* have no such markings.

##### Description.

**Male, adult. *Head*** (Fig. 9B, C). Vertex wide, strongly raised, black, moderately covered with short black hairs, with sparse long hyaline hairs anteriorly. Frons black; clypeus yellow, with black marking extending from frons. Antenna black, short, with slightly defined club, densely covered with short black hairs; flagellum comprising ~30 flagellomeres. Mouthparts yellowish brown; labrum yellowish brown, with several black hairs; maxillary palpus mostly dark brown; labial palpus mostly dark brown, spindle-shaped.

***Thorax*** (Fig. 9C). Pronotum broad, length shorter than width, dark brown, with yellow anterior corners, with hyaline hairs and long black hairs. Mesonotum dark brown, darker anteriorly, with yellowish white hairs. Metanotum dark brown, with pair of yellow spots at the middle, with yellowish white hairs.

***Legs*.** Coxae dark brown, moderately covered with long brown hairs. Femora yellow, moderately covered with black setae. Tibiae yellow; moderately covered with black setae; foretibia largely dark brown; midtibia dark brown at distal end; hind tibia dark brown at distal end and ventral surface. Tibial spurs reddish brown, short, almost straight, approximately as long as length of tarsomere 1. Tarsi dark brown, tarsomere 5 shorter than combined lengths of tarsomeres 1–4; claws reddish brown; short; curved.

***Wings*** (Fig. 9A). Without marking. Forewing veins and crossveins mostly dark brown; presectoral area with 6–8 crossveins; RP arising beyond CuA fork; CuP supporting one cell before fusing with 1A; 3A fused with 2A; pterostigma yellowish white; anterior Banksian line indistinct, posterior Banksian line distinct. Hindwings shorter and narrower than forewings; presectoral area with five crossveins; RP arising beyond MP fork; pterostigma yellowish white; anterior Banksian line absent, posterior Banksian line distinct; male with pilula axillaris.

***Abdomen*** (Fig. 9A). Shorter than hindwing, dark brown, densely covered with hyaline hairs.

***Genitalia*** (Fig. 9D, E, H–K). Ectoproct rectangular in lateral view, covered with long black setae. Sternite IX narrow, covered with long black setae. Gonarcus brown, arched, with long lateral arms. Mediuncus well sclerotized, black, waterdrop-shaped in lateral view. Parameres well sclerotized, dark brown, triangular in ventral view.

Size. BL: 21.1–28.7 mm; FWL: 22.8–30.2 mm; HWL: 21.0–28.6 mm.

**Female, adult.** Except terminalia, generally similar to male. Pilula axillaris absent. Terminalia (Fig. 9F, G): tergite VIII wider than tergite IX; tergite IX narrow, semicircular in lateral view; ectoproct semicircular in lateral view; lateral gonapophyses semicircular in lateral view, smaller than ectoproct; posterior gonapophyses short; with long black setae; anterior gonapophyses small, with long black setae; pregenital plate distinct, semicircular, presented on posterior margin of sternite VII.

Size. BL: 24.5–27.3 mm; FWL: 26.8–32.9 mm; HWL: 24.2–30.6 mm.

**Larva, 3^rd^ instar.** General color yellowish brown, with dark brown markings (Fig. 10A–C). Head triangular, longer than wide, with an anterior large dark marking and a V-shaped dark brown marking on dorsal side, with a pair of dark brown spots on ventral side; with a pair of dark brown spots in lateral side; mandibles yellowish brown; interdental mandibular setae (5) (2–3) (1–3) (1); external setae long (Fig. 10D, E). Abdominal sternite VIII with long black setae. Abdominal sternite IX with sparse ventral digging setae and four short digging setae in front of rastra; a paired rastra each with four digging setae (Fig. 10F).

Size. BL: 8.7 mm; HL: 2.1 mm, HW: 1.5 mm, ML: 2.3 mm.

##### Biological notes.

*Myrmeleon bore* is a species that is frequently observed around sandy environments throughout South Korea (Fig. 37B) with adults emerging from June. Larvae are pit builders. They were collected in various sandy environments such as coastal dunes, riverbanks, and dried-up valleys (Fig. 36C).

##### Distribution.

Korea, Japan, China, Russia, Uzbekistan, Europe (Stange 2004; Sekimoto 2014).

##### Remarks.

*Myrmeleon bore* was first reported from Korea by Kuwayama (1959). *Enza otiosus* Navás, 1912 has been considered a synonym of *M. bore* by Stange (2004), Sekimoto (2014), and Wang et al. (2018). However, based on the priority of the nomenclature of the International Code of Zoological Nomenclature (ICZN 1999), *M. bore* should be treated as a synonym of *M. otiosus*. The relationship between both species needs further research because the type localities of *E. otiosus* (holotype in Japan) and *M. bore* (syntypes in Sweden and Norway) are a great distance apart (Hassan et al. 2022).

#### ﻿ Myrmeleon formicarius

Taxon classificationAnimaliaNeuropteraMyrmeleontidae

Linnaeus, 1767

CC882A2F-3624-5581-9991-EB17F04C3E2D

**Figure 11.**
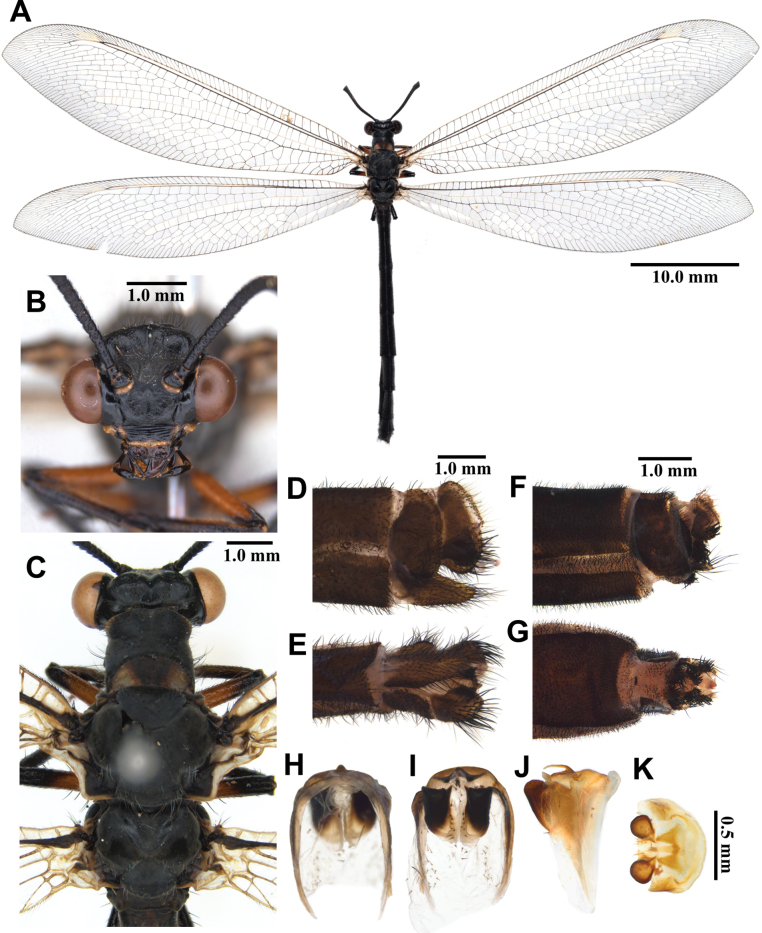
*Myrmeleon formicarius* (Linnaeus, 1758), adult. A. Dorsal habitus, male; B. Head, frontal view; C. Head and thorax, dorsal view; D, E. Male terminalia: D. Lateral view; E. Ventral view; F, G. Female terminalia: F. Lateral view; G. Ventral view; H–K. Male genitalia: H. Dorsal view; I. Ventral view; J. Lateral view; K. Caudal view.

**Figure 12.**
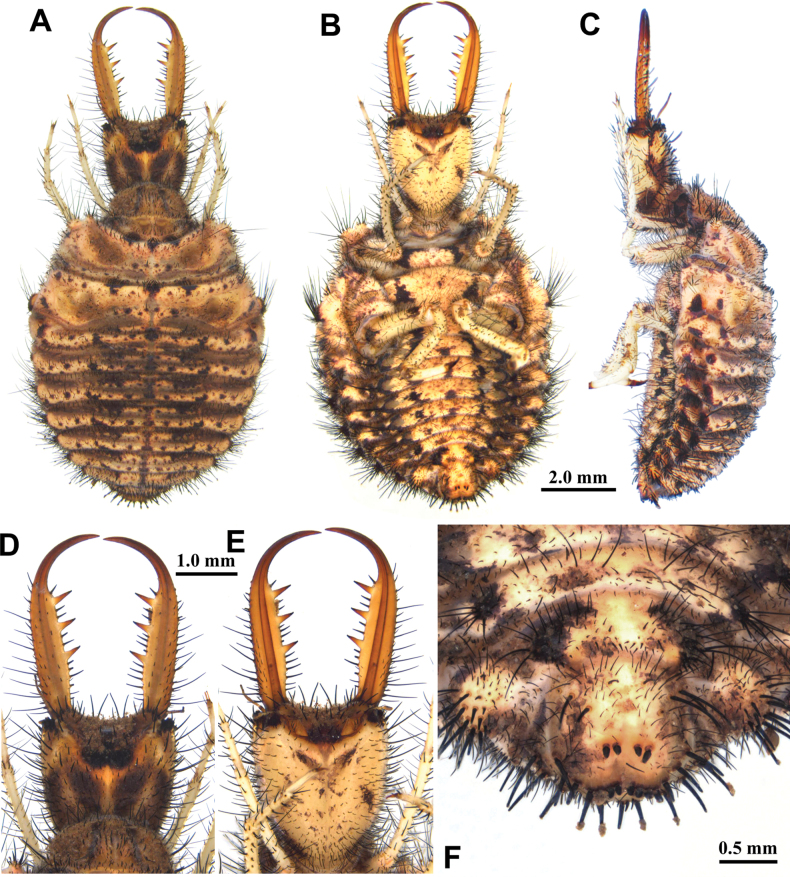
*Myrmeleon formicarius* (Linnaeus, 1758), third instar larva. A–C. Habitus: A. Dorsal view; B. Ventral view; C. Lateral view; D, E. Head: D. Dorsal view; E. Ventral view; F. Abdominal sternite IX.

Figs 11, 12, 35E, 36D, 37B

Myrmeleon formicarius Linnaenus, 1767: 914. Type locality: Europe.
Hemerobius formicalynx Linnaeus, 1758: 550. Type locality: Africa.
Myrmeleon neutrus Fischer von Waldheim, 1822: 51. Type locality: Russia: Siberia, Nerchinsk.
Myrmeleon innotatus Rambur, 1842: 406. Type locality: Hungary.
Myrmeleon nigrivenosus Okamoto, 1905: 116. Type locality: Japan: near Sapporo.
Myrmeleon formicarius formicarius Linnaeus, 1767: Steinmann 1963: 216.
Myrmeleon formicarius nigrilabrus Steinmann, 1963: 216. Type locality: Hungary: Vértes.

##### Specimens examined.

[**JBNU**] • 1♀ (reared from larva), Changwon-ri, Nam-myeon, Yeongwol-gun, Gangwon-do, Korea, 25.VI.2023, J.S. Kim; • 1♂, Palmi-ri, Sindong-myeon, Chuncheon-si, Gangwon-do, Korea, 14.V.2024, J.S. Kim; • 1♂1♀, Changwon-ri, Nam-myeon, Yeongwol-gun, Gangwon-do, Korea, 18.V.2024, J.S. Kim; • 1♀, Changwon-ri, Nam-myeon, Yeongwol-gun, Gangwon-do, Korea, 12.VI.2024, J.S. Kim; • 1♀, Gilgok-ri, Maehwa-myeon, Uljin-gun, Gyeongsangbuk-do, Korea, 21.VI.2024, Y.T. Jang; 1 larva (3^rd^ instar), Yulji-ri, Susan-myeon, Jecheon-si, Chungcheongbuk-do, 24.VI.2023, 1 larva (3^rd^ instar), Changwon-ri, Nam-myeon, Yeongwol-gun, Gangwon-do, Korea, 25.VI.2023, J.S. Kim; • 2 larvae (3^rd^ instar), Changwon-ri, Nam-myeon, Yeongwol-gun, Gangwon-do, Korea, 12.VI.2024, J.S. Kim.

##### Diagnosis.

Compared to other species in the genus *Myrmeleon*, *M. formicarius* has the morphological characteristics of an indistinct posterior Banksian line in the forewing, the femora being approximately half reddish orange, a mostly pale yellow MA in both the forewings and hindwings, and the pilula axillaris is absent. Larvae of *M. formicarius* have some dark markings on the hind coxa. Abdominal sternite IX with four short digging setae and two short rastra each with four digging setae.

##### Description.

**Male, adult. *Head*** (Fig. 11B, C). Vertex wide, strongly raised, black. Frons black, with short hyaline hairs; clypeus yellow, with black marking extending from frons to ventral 4/5. Antenna black, short, with slightly defined club, covered with short black hairs; flagellum comprising ~35 flagellomeres. Mouthparts dark brown; labrum dark brown, with several brown hairs; maxillary palpus dark brown; labial palpus dark brown, spindle-shaped.

Thorax (Fig. 11B). Pronotum broad, length shorter than width, dark brown, with yellow anterior corners, with hyaline hairs and long lateral black hairs. Mesonotum and metanotum dark brown; with long yellowish white hairs.

***Legs*.** Coxae dark brown, moderately covered with long yellowish white hairs. Femora reddish orange; dark brown on distal 1/2; moderately covered with black setae. Tibiae dark brown; moderately covered with black setae. Tibial spurs reddish brown, short, almost straight, approximately as long as length of Ta1. Tarsi dark brown, Tarsomere 5 shorter than combined lengths of tarsomeres 1–4; claws reddish brown.

***Wings*** (Fig. 11A). Without marking. Forewings veins and crossveins dark brown and pale yellow; presectoral area with 10–13 crossveins; RP arising beyond CuA fork; CuP supporting one cell before fusing with 1A; 3A fused with 2A; pterostigma yellowish white; anterior Banksian line indistinct, posterior Banksian line indistinct. Hindwing shorter and narrower than forewing; presectoral area with 5–7 crossveins; RP arising beyond MP fork; pterostigma yellowish white; anterior Banksian line indistinct, posterior Banksian line indistinct; male without pilula axillaris.

***Abdomen*** (Fig. 11A). Shorter than hindwing, dark brown, densely covered with short black hairs and hyaline hairs.

***Genitalia*** (Fig. 11D, E, H–K). Ectoproct semicircular, covered with long black setae. Sternite IX narrow, covered with long black setae. Gonarcus dark brown, arched, with long lateral arms. Mediuncus lightly sclerotized, dark brown, strongly hooked in lateral view. Parameres well sclerotized, black, rectangular in ventral view.

Size. BL: 29.9–31.1 mm; FWL: 35.1–36.0 mm; HWL: 32.3–33.7 mm.

**Female, adult.** Except terminalia, generally similar to male. Terminalia (Fig. 11F, G): tergite VIII wider than tergite IX; tergite IX narrow, semicircular in lateral view; ectoproct semicircular in lateral view; lateral gonapophyses semicircular in lateral view, smaller than ectoproct; posterior gonapophyses long, with long black setae; anterior gonapophyses small, with long black setae; pregenital plate distinct, rectangular, presented on posterior margin of sternite VII.

Size. BL: 29.3–33.1 mm; FWL: 36.3–39.3 mm; HWL: 33.4–36.8 mm.

**Larva, 3^rd^ instar.** General color reddish brown, with dark brown markings (Fig. 12A–C). Head triangular, longer than wide, with an anterior large dark marking and a V-shaped dark brown marking on dorsal side; with two pairs of dark brown spots on ventral side; with a pair of dark brown spots in lateral side; mandibles reddish brown; interdental mandibular setae (6) (2–4) (2–3) (1); external setae long (Fig. 12D, E). Hind coxa with some dark markings (Fig. 12B, C). Abdominal sternite VIII with sparse black setae. Abdominal sternite IX with four short digging setae in front of rastra; a paired rastra each with four digging setae (Fig. 12F).

Size. BL: 9.5 mm; HL: 2.6 mm, HW: 2.2 mm, ML: 2.9 mm.

##### Biological notes.

*Myrmeleon formicarius* is distributed locally in Gangwon-do, Chungcheongbuk-do, and Gyeongsangbuk-do in South Korea (Fig. 37B), primarily observed around grasslands environments such as logged areas and cemeteries. Adults emerge earlier (from May) than those of other species in South Korea. Larvae are pit builders, collected from dry, fine soil in open grassland environments or on cut slopes beside trails (Fig. 36D).

##### Distribution.

Korea, Japan, China, Russia, Tajikistan, Kyrgyzstan, Kazakhstan, Iran, Armenia, Turkey, Egypt, Europe (Sekimoto 2014; Yang et al. 2023).

##### Remarks.

*Myrmeleon formicarius* is is widely distributed in the Palaearctic Region; Okamoto (1926) first reported this species from Korea. However, Kuwayama (1959) confirmed only *Grocus bore* Tjeder, 1941 from Korea and noted that he could not confirm any Korean specimens of *M. formicarius*. In this study, we confirm the presence of *M. formicarius* in Korea and report it with a detailed description and illustrations.

#### ﻿ Myrmeleon immanis

Taxon classificationAnimaliaNeuropteraMyrmeleontidae

Walker, 1853

638D5ADD-2904-5970-8E8F-8EEA2CCA0C05

**Figure 13.**
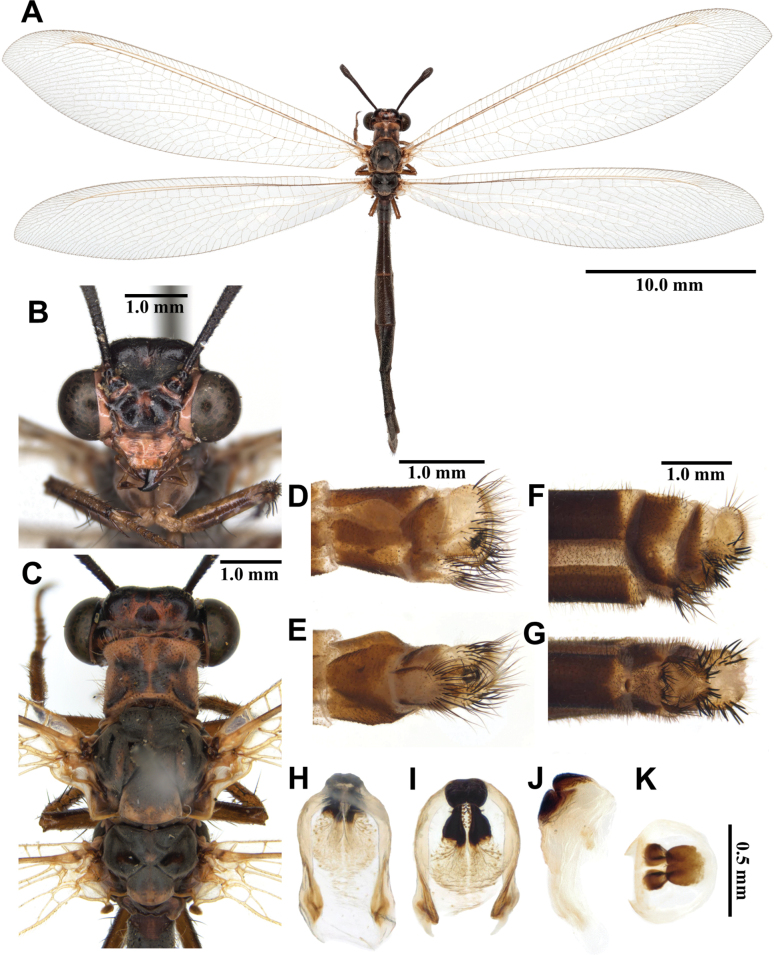
*Myrmeleon immanis* Walker, 1853, adult. A. Dorsal habitus, male; B. Head, frontal view; C. Head and thorax, dorsal view; D, E. Male terminalia: D. Lateral view; E. Ventral view; F, G. Female terminalia: F. Lateral view; G. Ventral view; H–K. Male genitalia: H. Dorsal view; I. Ventral view; J. Lateral view; K. Caudal view.

**Figure 14.**
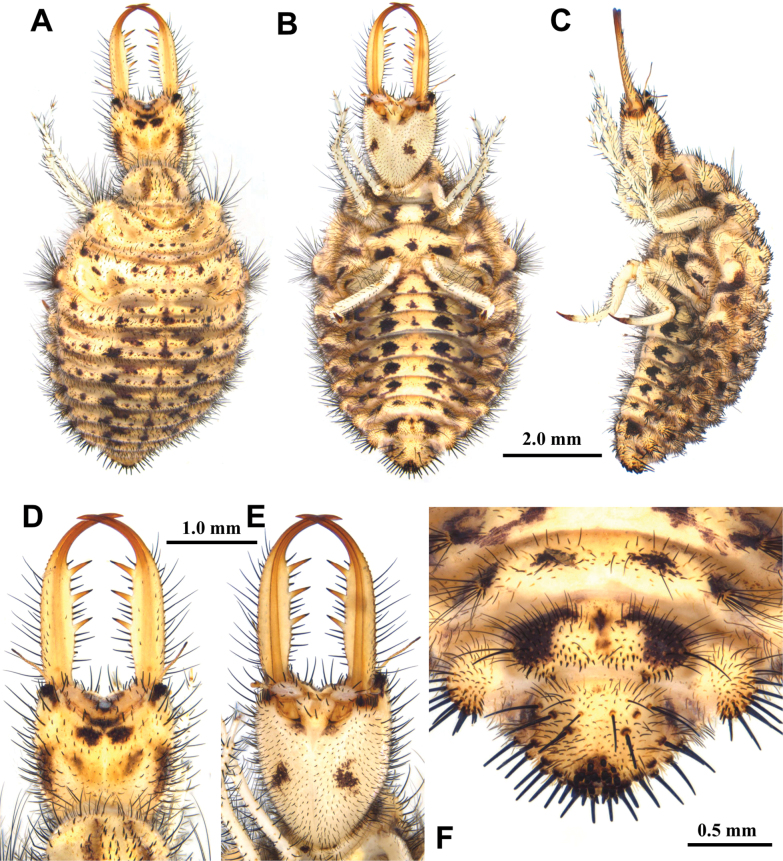
*Myrmeleon immanis* Walker, 1853, third instar larva. A–C. Habitus: A. Dorsal view; B. Ventral view; C. Lateral view; D, E. Head: D. Dorsal view; E. Ventral view; F. Abdominal sternite IX.

Figs 13, 14, 35F, 36E, 37B

Myrmeleon immanis Walker, 1853: 381. Type locality: China.
Myrmeleon medialis Navás, 1932: 110. Type locality: Russia: Buryatiya: Chikoy.
Myrmeleon procubitalis Navás, 1935:41. Type locality: Russia: Buryatiya: Chikoy.
Grocus pallens Hölzel, 1970b: 255. Type locality: Mongolia: Bulgan.

##### Specimens examined.

[**JBNU**] • 3♂2♀, Sindu-ri, Wonbuk-myeon, Taean-gun, Chungcheongnam-do, Korea, 20.VIII.2022, J.S. Kim; • 2♂, Sindu-ri, Wonbuk-myeon, Taean-gun, Chungcheongnam-do, Korea, 17.VI.2023, J.S. Kim; • 1♂1♀ (reared from larva), Sindu-ri, Wonbuk-myeon, Taean-gun, Chungcheongnam-do, Korea, 18.VI.2023, J.S. Kim; • 1♀, Sindu-ri, Wonbuk-myeon, Taean-gun, Chungcheongnam-do, Korea, 31.VII.2024, J.S. Kim; • 3 larvae (3^rd^ instar), Sindu-ri, Wonbuk-myeon, Taean-gun, Chungcheongnam-do, Korea, 18.VI.2023, J.S. Kim.

##### Diagnosis.

In *Myrmeleon immanis*, wing veins and crossveins are yellowish brown. The pronotum has yellow anterior and lateral margins that form an M-shaped yellow marking. The vertex has yellow markings, while in *M. bore* and *M. formicarius*, the vertex is only black. In larvae, abdominal sternite IX has dense short digging setae in front of the rastra.

##### Description.

**Male, adult. *Head*** (Fig. 13B, C). Vertex wide, slightly raised, reddish brown. Frons dark brown, with yellow spot at middle; clypeus yellow, with long pale brown hairs. Antenna dark brown, short, with slightly defined club, densely covered with short black hairs; flagellum comprising ~30 flagellomeres. Mouthparts brown; labrum yellow, with several hyaline black hairs; maxillary palpus brown; labial palpus brown, spindle-shaped.

***Thorax*** (Fig. 13C). Pronotum broad, length shorter than width, dark brown, yellow anterior and lateral margins forming M-shaped yellow marking, covered with hyaline hairs. Mesonotum and metanotum dark brown, covered with several hyaline hairs.

***Legs*.** Coxae moderately covered with long white hairs; forecoxae mostly yellowish brown, midcoxa and hind coxa dark brown. Femora mostly yellowish brown, partly dark brown; moderately covered with black hairs. Tibiae yellowish brown; moderately covered with black hairs. Tibial spurs reddish brown proximally, dark brown distally, short, almost straight, in forelegs and midlegs approximately as long as combined lengths of tarsomeres 1–4, in hindleg approximately as long as length of tarsomere 1. Tarsi yellowish white, tarsomere 5 as long as combined lengths of tarsomeres 1–4; claws reddish brown.

***Wings*** (Fig. 13A). Without markings. Forewings veins and crossveins yellowish brown; presectoral area with seven or eight crossveins; RP arising beyond CuA fork; CuP supporting one cell before fusing with 1A; 2A fused with 3A; pterostigma white; anterior Banksian line absent, posterior Banksian line distinct. Hindwing shorter and narrower than forewing; anterior Banksian lines absent; presectoral area with five crossveins; RP arising beyond MP fork; pterostigma white; posterior Banksian lines distinct; male with pilula axillaris.

***Abdomen*** (Fig. 13A). Shorter than hindwing, dark brown, densely covered with short hyaline hairs.

***Genitalia*** (Fig. 13D, E, H–K). Ectoproct rectangular, covered with long black setae. Sternite IX narrow, covered with long black setae. Gonarcus pale brown, arched, with long lateral arms. Mediuncus well sclerotized, black, rectangular in ventral view. Parameres well sclerotized, black, triangular in ventral view.

Size. BL: 21.8–24.6 mm; FWL: 22.7–25.7 mm; HWL: 21.0–23.4 mm.

**Female, adult.** Except terminalia, generally similar to male. Pilula axillaris absent. Terminalia (Fig. 13F–G): tergite VIII wider than tergite IX; tergite IX narrow, triangular in lateral view; ectoproct semicircular in lateral view; lateral gonapophyses semicircular in lateral view, smaller than ectoproct; posterior gonapophyses long; with long black setae; anterior gonapophyses small, with long black setae; pregenital plate distinct, semicircular, presented on posterior margin of sternite VII.

Size. BL: 21.8–24.6 mm; FWL: 22.7–25.7 mm; HWL: 21.0–23.4 mm.

**Larva, 3^rd^ instar.** General color yellowish white, with dark brown markings (Fig. 14A–C). Head triangular, longer than wide, with an anterior pair of spots and a pale V-shaped marking on dorsal side; with a pair of dark brown spots on ventral side; with a pair of dark brown spots in lateral side; mandibles yellowish white; interdental mandibular setae (7–9) (3) (2) (1); external setae long (Fig. 14D, E). Legs yellowish white (Fig. 14B, C). Abdominal sternite VIII with sparse black setae. Abdominal sternite IX with short dense digging setae in front of rastra; a paired rastra each with four digging setae (Fig. 14F).

Size. BL: 7.8 mm; HL: 1.8 mm, HW: 1.5 mm, ML: 1.9 mm.

##### Biological notes.

*Myrmeleon immanis* is distributed some coastal environments of Chungcheongnam-do in South Korea (Fig. 37B). This species has been observed around sandy environments with well-developed coastal dunes. Adults emerge from June in South Korea. Larvae are pit builders. Unlike the larvae of *M. bore*, which are observed in various sandy environments, the larvae of this species were collected restrictively only in foredunes where *Leymus* (Poaceae) grows (Fig. 36E).

##### Distribution.

Korea (new record), China, Russia, Mongolia, Romania, Indochina (Wang et al. 2018).

##### Remarks.

*Myrmeleon immanis* is the smallest species of the genus in Korea. In Japan, a morphologically similar species, *Myrmeleon solers* Walker, 1853, is known to be distributed. Although *M. solers* has also been recorded from China by Kuwayama (1959), according to Wang et al. (2018), Chinese specimens of the could not be confirmed, suggesting the possibility that *M. solers* is an endemic species to Japan.

### ﻿Subfamily Dendroleontinae Banks, 1899

**Tribe Dendroleontini Banks, 1899**

#### ﻿Genus Dendroleon

Taxon classificationAnimaliaNeuropteraMyrmeleontidae

Brauer, 1866

E6F1E7E4-1017-5112-9B81-F150EC0BEA13

Dendroleon Brauer, 1866: 42. Type species: Myrmeleon pantherinus Fabricius, 1787. Type locality: Austria.
Borbon Navás, 1914b: 111.
Neglurus Navás, 1912c: 171.
Pantherleon Yang, 1986: 431.

##### Diagnosis.

Adult. Medium to large sized antlions; antenna long; wing with brown markings; forewing presectoral area usually with four crossveins; forewing vein RP arising usually before CuA fork; forewing veins 2A and 3A separate, usually connected by one or two crossveins; hindwing presectoral area usually with one crossvein; hindwing vein RP arising before MP fork; male with pilula axillaris. Legs long and slender, hind femur plus tibia longer than entire length of head plus thorax; tibial spurs approximately as long as combined lengths of tarsomeres 1 and 2. Female gonapophyses divided into tubercular or short digitiform anterior and long digitiform posterior branch; a pair of gonapophyses with slender sclerotized setae (Sekimoto 2014; Zheng et al. 2024a). Third instar larva. Mandibles upturned, with three pairs of equidistant teeth; mesothoracic and abdominal spiracles not prominent; thoracic setiferous processes pedunculated; abdominal sternite VIII without odontoid processes; abdominal sternite IX longer than wide, triangular; rastra and fossoria weakly developed (Zheng et al. 2024a).

##### Distribution.

Asia (Azerbaijan, China, Georgia, India, Indonesia, Japan, Malaysia, Mongolia, Russian (Far East), Vietnam), Europe (Austria, Bulgaria, Croatia, Czech Republic, Finland, France, Germany, Hungary, Italy, Malta, Poland, Romania, Russian Caucasus, Slovakia, Slovenia, Switzerland, Turkey, Ukraine), North America (Canada, Mexico, the United States), Oceania (Australia, Papua New Guinea) (Zheng et al. 2024a).

#### ﻿ Dendroleon pupillaris

Taxon classificationAnimaliaNeuropteraMyrmeleontidae

(Gerstaecker, 1893)

24B6DDCA-9340-5226-A075-B3745B285ED8

Figs 15, 37C

Glenurus pupillaris Gerstaecker, 1893: 120. Type locality: Japan: Honshu: Kanto: Kanagawa: Yokohama.
Dendroleon pupillaris (Gerstaecker, 1893): Okamoto 1914: 249.

##### Specimens examined.

[**JBNU**] 1♀, Buun-ri, Sannae-myeon, Namwon-si, Jeonbuk-do, Korea, 22.VIII.2023, H. Han; • 1♂, Gancheok-ri, Gandong-myeon, Hwacheon-gun, Gangwon-do, Korea, 23.VIII.2024, J.S. Kim.

##### Diagnosis.

*Dendroleon pupillaris* is distinguished from other species of the *D. pupillaris* group by its continuous forewing cubital curved stripe. The pronotum has a faint dark brown longitudinal middle stripe.

##### Description.

**Figure 15.**
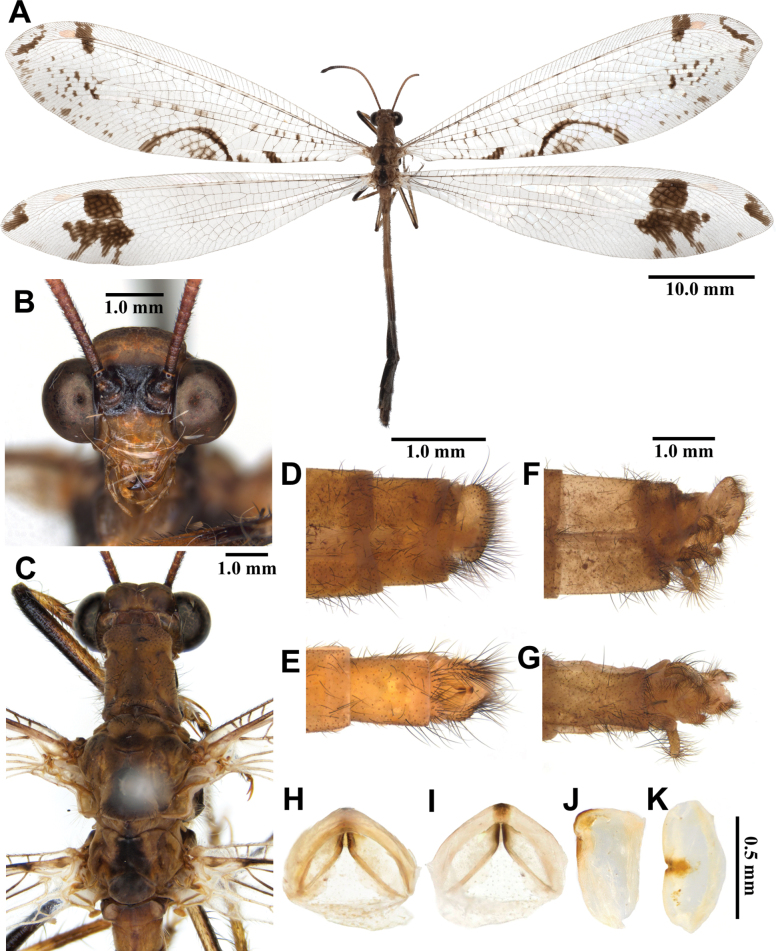
*Dendroleon pupillaris* (Gerstaecker, 1893), adult. A. Dorsal habitus, male; B. Head, frontal view; C. Head and thorax, dorsal view; D, E. Male terminalia: D. Lateral view; E. Ventral view; F, G. Female terminalia: F. Lateral view; G. Ventral view; H–K. Male genitalia: H. Dorsal view; I. Ventral view; J. Lateral view; K. Caudal view.

**Male, adult. *Head*** (Fig. 15B, C). Vertex slightly narrow, strongly raised, yellowish brown. Frons yellow, with broad dark brown band at middle; clypeus yellow, with long black hairs. Antenna reddish brown, long, with slightly defined club, densely covered with short black hairs; flagellum comprising ~35 flagellomeres. Mouthparts yellowish brown; labrum yellow, with hyaline brown hairs; maxillary palpus yellow; labial palpus yellow.

***Thorax*** (Fig. 15C). Pronotum slender, longer than broad, yellowish brown, with slender dark brown longitudinal middle stripe. Mesonotum and metanotum yellowish brown, with broad dark brown longitudinal median stripe.

***Legs*.** Coxae yellow, moderately covered with long pale yellow hairs. Femora mostly dark brown; partly yellow; moderately covered with black setae. Tibiae mostly yellow; partly dark brown; densely covered with short black hairs. Tibial spurs yellowish brown, long, slight curved, approximately as long as combined lengths of tarsomeres 1 and 2. Tarsi dark brown, tarsomere 5 shorter than combined lengths of tarsomeres 1–4; claws yellowish brown.

***Wings*** (Fig. 15A). With brown markings. Forewings attractively marked with shades of brown; veins and crossveins with white and brown; presectoral area with three or four crossveins; RP arising before CuA fork; CuP supporting two or three cells before fusing with 1A; 2A and 3A separate, connected by two crossveins; pterostigma pale pink and white; anterior Banksian line distinct, posterior Banksian line absent. Hindwings approximately as long as forewings, narrower than forewings, with large brown marking extending from proximal part of pterostigma to posterior margin; presectoral area with one crossvein; RP arising before MP fork; pterostigma pale pink and white; anterior Banksian line distinct; posterior Banksian line absent; male with pilula axillaris.

***Abdomen*** (Fig. 15A). Shorter than hindwing, reddish brown, densely covered with black hairs.

Genitalia (Fig. 15D, E, H–K). Ectoproct semicircular, covered with long black setae. Sternite IX narrow, covered with long black setae. Gonarcus brown, arched. Mediuncus lightly sclerotized, dark brown, moderately raised. Parameres broad, dark brown posteriorly, well sclerotized posteriorly.

Size. BL: 29.3 mm; FWL: 35.0 mm; HWL: 35.0 mm.

**Female, adult.** Except terminalia, generally similar to male. Pilula axillaris absent. Terminalia (Fig. 15F, G): tergite VIII wider than tergite IX; tergite IX narrow, rectangular in lateral view; ectoproct oval in lateral view; a pair of gonapophyses present below tergite IX; lateral gonapophyses smaller than ectoproct with long black setae; posterior gonapophyses long, curved, with long black setae; anterior gonapophyses long, with long black setae; pregenital plate distinct, small, triangular, presented on membrane below tergite VIII.

Size. BL: 29.1 mm; FWL: 36.8 mm; HWL: 37.0 mm.

##### Biological notes.

*Dendroleon pupillaris* is a species that is restricted in areas with well-developed mountains, such as Jiri Mountain and Gangwon-do in South Korea (Fig. 37C). Adults emerge later than those of other species, mainly from August to September, in South Korea. For detailed information on larval ecology, refer to Matsumoto et al. (2016).

##### Distribution.

Korea, Japan (Woo et al. 1998; Zheng et al. 2024a).

##### Remarks.

*Dendroleon pupillaris* was first recorded in Korea by Woo et al. (1998) on a checklist, without any taxonomic description or

##### remarks.

This record was referred to and included in the checklist of Paek (2010). In other countries, *D. pupillaris* has been described as a species only recorded in Japan (Sekimoto 2014; Zheng et al. 2024a). In this study, we confirm its distribution in Korea and provide a detailed description and illustrations.

#### ﻿Genus Nepsalus

Taxon classificationAnimaliaNeuropteraMyrmeleontidae

Navás, 1914

AA1E7393-AF97-5B5F-9134-240E91922BC1

Nepsalus Navás, 1914a: 250. Type species: Nepsalus indicus Navás, 1914. Type locality: Malaysia: Gunong Inas.

##### Diagnosis.

Adult. Wings with some broad brown markings; anterior Banksian line present; rhegma area with dark brown spot; forewing cubital area with a brown marking; male pilula axillaris present (Zheng et al. 2022, 2024b). Third instar larva. Mandible hardly upturned, with pseudo-teeth developed in addition to three normal teeth. Mesothoracic tuft of long hair-like setae absent. Thoracic setiferous processes pedunculated and well developed. Abdomen generally green with many dark brownish markings; a pair of setiferous processes present on each side of abdominal segments 2–7; abdominal sternite VIII without odontoid processes; abdominal sternite IX longer than wide, triangular (Zheng et al. 2022).

##### Distribution.

Asia (China, India, Japan, Korea peninsula, Malay peninsula, Russia (Far East), Vietnam) (Zheng et al. 2022).

#### ﻿ Nepsalus jezoensis

Taxon classificationAnimaliaNeuropteraMyrmeleontidae

(Okamoto, 1910)

C07DF553-7D18-5967-AEA2-50FB66B0B21F

**Figure 16.**
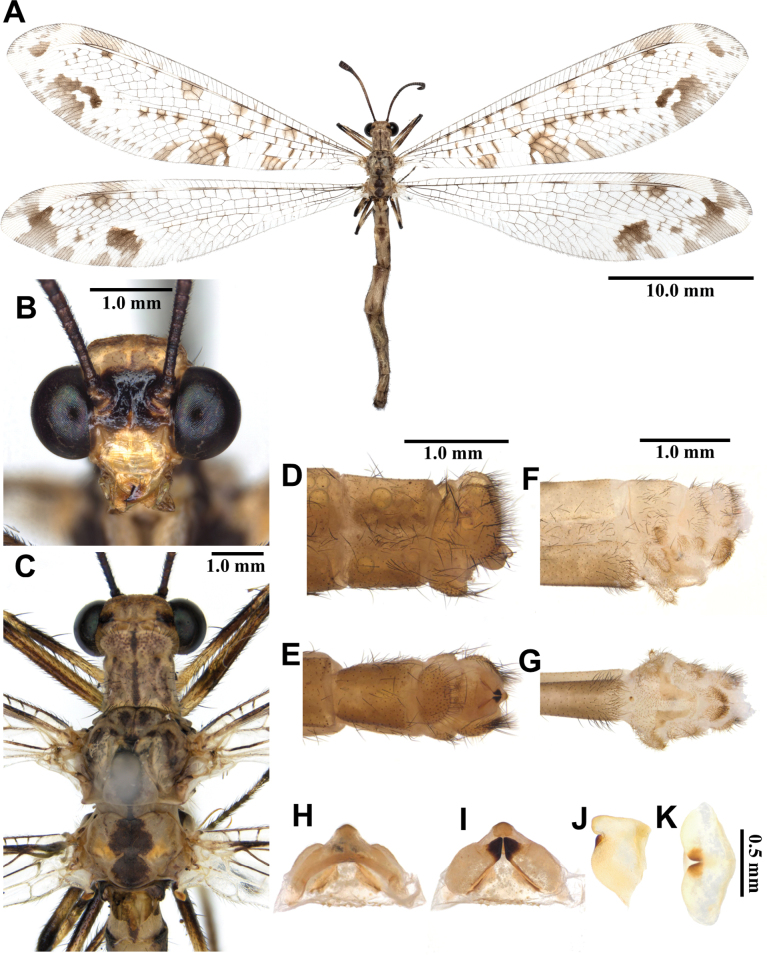
*Nepsalus jezoensis* (Okamoto, 1910), adult. A. Dorsal habitus, male; B. Head, frontal view; C. Head and thorax, dorsal view; D, E. Male terminalia: D. Lateral view; E. Ventral view; F, G. Female terminalia: F. Lateral view; G. Ventral view; H–K. Male genitalia: H. Dorsal view; I. Ventral view; J. Lateral view; K. Caudal view.

**Figure 17.**
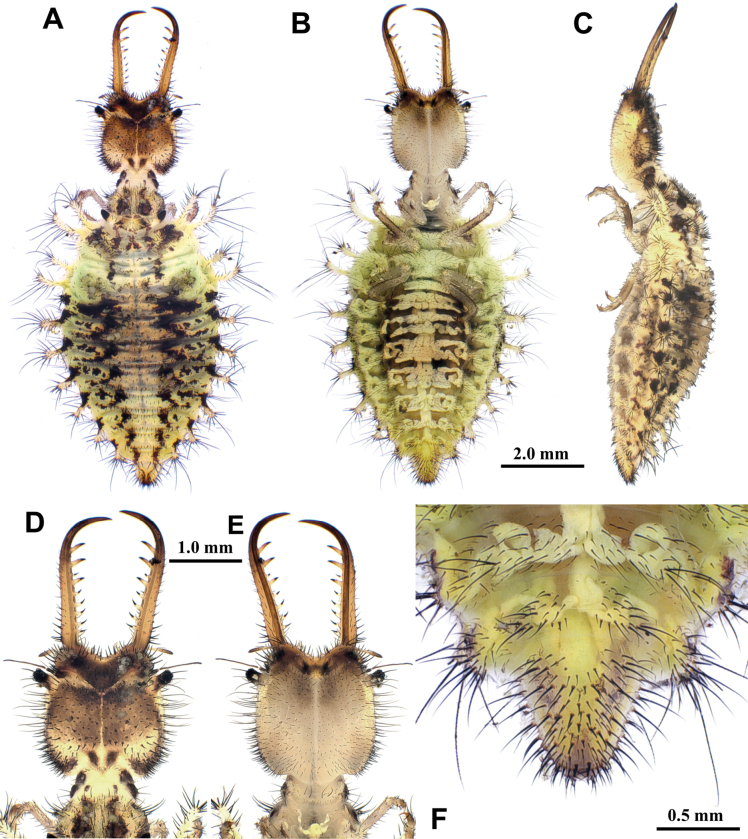
*Nepsalus jezoensis* (Okamoto, 1910), third instar larva. A–C. Habitus: A. Dorsal view; B. Ventral view; C. Lateral view; D, E. Head: D. Dorsal view; E. Ventral view; F. Abdominal sternite IX.

Figs 16, 17, 35G, 36F, 37C

Dendroleon jezoensis Okamoto, 1910: 280. Type locality: Japan: Honshu: Kanto: Tochigi: Nikko.
Glenurus jezoensis Matsumura, 1908: 41. nom. nud.
Gatzara jezoensis (Okamoto, 1910): Miller et al. 1999: 52.
Nepsalus jezoensis (Okamoto, 1910): Zheng et al. 2022: 17.

##### Specimens examined.

[**JBNU**] 1♀, Samjung-ri, Macheon-myeon, Hamyang-gun, Gyeongsangnam-do, Korea, 17.IX.2022, J.S. Kim; • 1♀ (reared form larva), Naejang-dong, Jeongeup-si, Jeonbuk-do, Korea, 14.VII.2023, J.S. Kim; • 7♂2♀ (reared form pupa), Jungsan-ri, Sicheon-myeon, Sancheong-gun, Gyeongsangnam-do, Korea, 25.VI.2024, J.S. Kim; • 1♂, Oeseonmi-ri, Onjeong-myeon, Uljin-gun, Gyeongsangbuk-do, Korea, 13.VII.2024, J.S. Kim; • 1♀, Sa-ri, Heuksan-myeon, Sinan-gun, Jeollanam-do, Korea, 3.IX.2024, J.S. Kim; 1 larva (3^rd^ instar), Naejang-dong, Jeongeup-si, Jeonbuk-do, Korea, 14.VII.2023, J.S. Kim; • 3 larvae (2^nd^ and 3^rd^ instar), Wongi-ri, Gui-myeon, Wanju-gun, Jeonbuk-do, Korea, 8.III.2025, J.S. Kim; 1 larva (2^nd^ instar), Gaegok-ri, Daechi-myeon, Cheongyang-gun, Chungcheongnam-do, Korea, 15.III.2025, J.S. Kim; 1 larva (2^nd^ instar), Gwangdae-ri, Daechi-myeon, Cheongyang-gun, Chungcheongnam-do, Korea, 16.III.2025, J.S. Kim; 1 larva (3^rd^ instar), Wongi-ri, Gui-myeon, Wanju-gun, Jeonbuk-do, Korea, 29.III.2025, J.S. Kim.

##### Diagnosis.

*Nepsalus jezoensis*is is similar to *Nepsalus insularum* Hayashi, Saito & Matsumoto, 2024 from southern Japan (Amamio, Okinawa, Kume, and Ishigaki islands) in general appearance. These two species can be distinguished by differences in the patterns on their wings, pronotum, and abdomen. In *N. jezoensis*, the forewing cubital area with an arcuate dark brown marking fused with a large brown spot, while they are not fused in *N. insularum. Nepsalus insularum* has wider blackish markings on the thorax, and larger dark brown markings on the abdomen. In larvae, the mandibles are equipped with five or six pseudo-teeth, and the legs are pale brown distally (Hayashi et al. 2024).

##### Description.

**Male, adult. *Head*** (Fig. 16B, C). Vertex slightly narrow, moderately raised, yellowish white. Frons yellow, with broad dark brown band at middle; clypeus yellow, with long black hairs. Antenna dark brown, slightly long, with slightly defined club, densely covered with short black hairs; flagellum comprising ~35 flagellomeres. Mouthparts yellow; labrum yellow, with dark and brown hairs; maxillary palpus yellowish brown; labial palpus yellowish brown.

***Thorax*** (Fig. 16C). Pronotum slender, longer than broad, yellowish white, with slender dark brown longitudinal median stripe. Mesonotum and metanotum yellowish white, with dark spot at middle.

***Legs*.** Coxae yellow, moderately covered with long black hairs. Femora mostly dark brown; partly yellow; moderately covered with black setae. Tibiae mostly dark brown; partly yellow; densely covered with short black hairs. Tibial spurs yellowish brown, long, slight curved, approximately as long as combined lengths of tarsomeres 1 and 2. Tarsi pale brown, tarsomere 5 shorter than combined lengths of tarsomeres 1–4; claws reddish brown, opposable.

***Wings*** (Fig. 16A). With brown markings. Forewings veins and crossveins mostly brown; presectoral area with 3–5 crossveins; RP arising before CuA fork; CuP supporting one or two cells before fusing with 1A; 2A and 3A separate, connected by one crossvein; pterostigma white; anterior Banksian line distinct, posterior Banksian line absent. Hindwing approximately as long as forewing, narrower than forewing, with small brown markings along posterior margin; presectoral area with one crossvein; RP arising before MP fork; pterostigma white; anterior Banksian line distinct, posterior Banksian line absent; male with pilula axillaris.

***Abdomen*** (Fig. 16A). Shorter than hindwing, yellowish white, moderately covered with black hairs.

***Genitalia*** (Fig. 16D, E, H–K). Ectoproct rectangular, covered with long black setae. Sternite IX narrow, covered with long black setae. Gonarcus yellowish brown, arched. Mediuncus lightly sclerotized, reddish brown, strongly raised. Parameres broad, dark brown posteriorly, well sclerotized posteriorly.

Size. BL: 18.2–21.8 mm; FWL: 23.2–26.7 mm; HWL: 22.1–26.1 mm.

**Female, adult.** Except terminalia, generally similar to male. Pilula axillaris absent. Terminalia (Fig. 16F, G): tergite VIII wider than tergite IX; tergite IX narrow, rectangular in lateral view; ectoproct semicircular in lateral view; a pair of gonapophyses present below tergite IX; lateral gonapophyses semicircular in lateral view, smaller than ectoproct; posterior gonapophyses long, with long black setae; anterior gonapophyses long, with long black setae; pregenital plate distinct, small, triangular, presented on membrane below tergite VIII.

Size. BL: 20.8–23.5 mm; FWL: 25.1–30.9 mm; HWL: 24.9–30.1 mm.

**Larva, 3^rd^ instar.** General color pale brown and pale green, with dark brown markings (Fig. 17A–C). Head rectangular, longer than wide, with a triangular dark brown spot anteriorly and a pair of dark brown spots on dorsal side; without marking on ventral side; with a pair of dark brown spots in lateral side; mandibles yellowish brown, with five or six pseudo-teeth developed in addition to three normal teeth; external setae short (Fig. 17D, E). Abdominal sternite VIII with sparse black setae. Abdominal sternite IX with sparse black setae (Fig. 17F).

Size. BL: 7.1 mm; HL: 1.9 mm, HW: 1.7 mm, ML: 2.5 mm.

##### Biological notes.

*Nepsalus jezoensis* is a species observed mainly in mountainous topography throughout South Korea (Fig. 37C) where adults mainly emerge from July to September. Larvae were collected on rock walls or tree bark where lichens grow densely, and they camouflage their bodies with lichens (Fig. 36F). In larval habitats, empty cocoons can be easily observed.

##### Distribution.

Korea, Japan, Russia (Zheng et al. 2022; Hayashi et al. 2024).

##### Remarks.

This species was originally classified in the genus *Gatzara* Navás, but it was reassigned to the genus *Nepsalus* Navás by Zheng et al. (2022) based on morphological and genetic analysis.

### ﻿Subfamily Nemoleontinae Banks, 1911

**Tribe Nemoleontini Banks, 1911**

#### ﻿Genus Deutoleon

Taxon classificationAnimaliaNeuropteraMyrmeleontidae

Navás, 1927

5D57B27E-DCBF-5F97-967D-49D349F71053

Deutoleon Navás, 1927b: 19. Type species: Deutoleon turanicus Navás, 1927. Type locality: Russia: Baikal.

##### Diagnosis.

Adult. Forewing presectoral area usually with seven crossveins. Hindwing with two presectoral crossveins. Hind tibial spurs at least twice as long as tarsomere 1. Male without pilula axillaris (Zhan et al. 2012).

##### Distribution.

Palearctic (Korea, China, Russia, Moldova, Mongolia, Ukraine, Hungary, Kazakhstan, Kyrgyzstan) (Krivokhatsky 2011; Szőke 2021).

#### ﻿ Deutoleon lineatus lineatus

Taxon classificationAnimaliaNeuropteraMyrmeleontidae

(Fabricius, 1798)

308DA52A-E725-5D15-B641-50AD4C848EE1

Figs 18, 32A, 37F

Myrmeleon lineatus Fabricius, 1798: 205. Type locality: Russia: southern.
Myrmeleon ornatus Olivier, 1811: 123. Type locality: Russia: southern.
Myrmeleon sibiricum Fischer Von Waldheim, 1822: 45. Type locality: Russia: Irkutsk: Siberia, near Irkutsk.
Formicaleo lineatus (Fabricius, 1798): Hagen 1866b: 404.
Myrmeleon ambiguus Klapálek, 1901: 209. Type locality: Russia: Minusinsk.
Distoleon lineatus (Fabricius, 1798): Kuwayama 1924: 82.
Deutoleon lineatus (Fabricius, 1798): Navás 1927b 18.
Deutoleon turanicus Navás, 1927b: 19. Type locality: Russia: Baikal.
Deutoleon lineatus lineatus (Fabricius, 1798): Krivokhatsky 2011: 124.

##### Specimens examined.

[**JBNU**] • 1♀, Yulji-ri, Susan-myeon, Jecheon-si, Chungcheongbuk-do, 24.VI.2023, J.S. Kim; • 3♂7♀, Changwon-ri, Nam-myeon, Yeongwol-gun, Gangwon-do, Korea, 12.VI.2024, J.S. Kim; • 1♂, Yulji-ri, Susan-myeon, Jecheon-si, Chungcheongbuk-do, 25.V.2025, J.S. Kim.

##### Diagnosis.

*Deutoleon lineatus lineatus* is similar to *D. lineatus turanicus* in general appearance. However, they can be easily distinguished because the forewing veins of *D. lineatus lineatus* are only yellow, while in *D. lineatus turanicus* they are alternately black and yellow.

##### Description.

**Figure 18.**
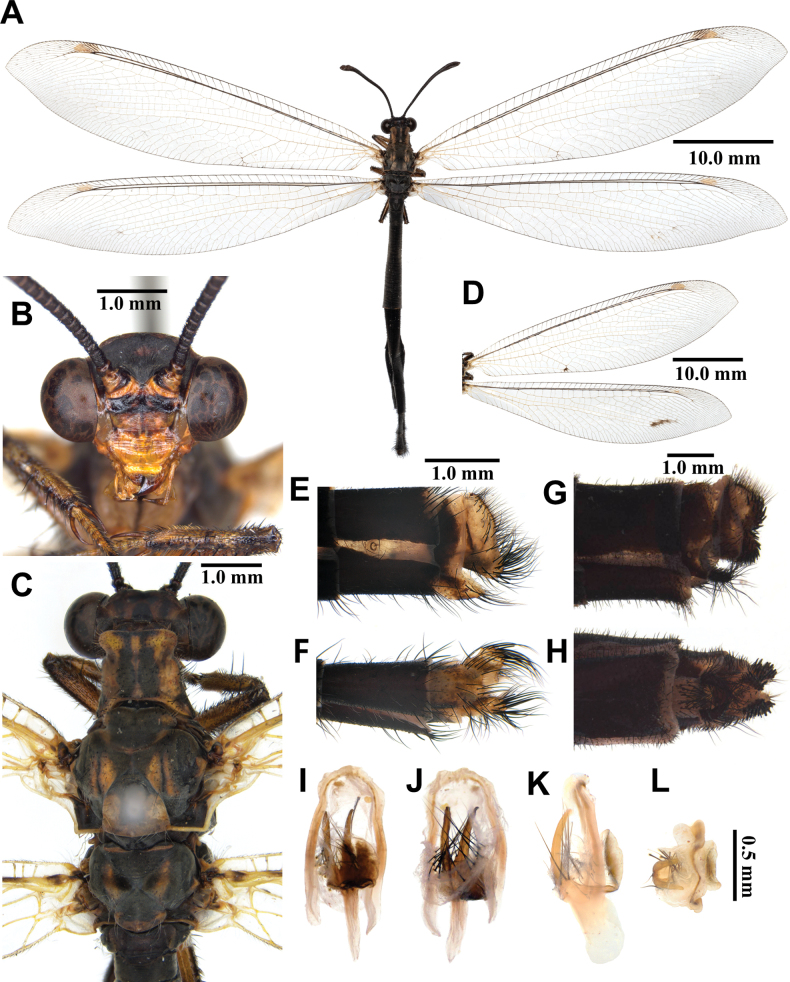
*Deutoleon lineatus* (Fabricius, 1798), adult. A. Dorsal habitus, male; B. Head, frontal view; C. Head and thorax, dorsal view; D. Wing, female; E, F. Male terminalia: E. Lateral view; F. Ventral view; G, H. Female terminalia: G. Lateral view; H. Ventral view; I–L. Male genitalia: I. Dorsal view; J. Ventral view; K. Lateral view; L. Caudal view.

**Male, adult. *Head*** (Fig. 18B, C). Vertex slightly wide, moderately raised, yellow. Frons yellow, with dark brown band in the middle; clypeus yellow, with long black hairs. Antenna dark brown, slightly long, with slightly defined club, densely covered with short black hairs; flagellum comprising ~45 flagellomeres. Mouthparts yellowish brown; labrum yellow, with hyaline brown hairs; maxillary palpus yellowish brown; labial palpus yellowish brown, spindle-shaped.

***Thorax*** (Fig. 11C). Pronotum slender, longer than broad, whitish-yellow, with slender dark brown longitudinal middle stripe. Mesonotum and metanotum yellowish white, with dark spot at middle.

***Legs*.** Coxae yellow, moderately covered with long black hairs. Femora mostly dark brown; partly yellow; moderately covered with black setae. Tibiae mostly dark brown; partly yellow; densely covered with short black hairs. Tibial spurs reddish brown, slightly long, slightly curved, approximately as long as combined lengths of tarsomeres 1 and 2. Tarsi pale brown, tarsomere 5 longer than combined lengths of tarsomeres 1–4; claws reddish brown.

***Wings*** (Fig. 18A). With dark brown markings. Forewings veins and crossveins mostly yellow; presectoral area with 7–9 crossveins; RP arising beyond CuA fork; CuP supporting one or two cells before fusing with 1A; 2A fused with 3A; pterostigma yellowish white; anterior Banksian lines distinct; posterior Banksian lines distinct. Hindwing approximately as long as forewing, narrower than forewing; presectoral area with two crossveins; RP arising before MP fork; pterostigma yellowish white; anterior Banksian and posterior Banksian lines absent; male without pilula axillaris.

***Abdomen*** (Fig. 18A). Dark brown, moderately covered with black hairs.

***Genitalia*** (Fig. 18E, F, I–L). Ectoproct covered with long black setae. Sternite IX narrow, covered with long black setae. Gonarcus reddish brown, arched. Mediuncus absent. Parameres well sclerotized, dark brown, strongly arched. Parameres well sclerotized, dark brown, with long black setae, moderately hooked in lateral view.

Size. BL: 33.9–35.7 mm; FWL: 35.9–38.9 mm; HWL: 35.1–38.3 mm.

**Female, adult.** Except terminalia, generally similar to male. Hindwing of female with one distinct dark brown stripe in rhegma area (Fig. 18D). Terminalia (Fig. 18G–H): tergite VIII slightly wider than tergite IX; tergite IX narrow, triangular in lateral view; ectoproct semicircular in lateral view; lateral gonapophyses semicircular in lateral view, smaller than ectoproct; posterior gonapophyses long, with long black setae; anterior gonapophyses absent; pregenital plate absent.

Size. BL: 32.8–36.2 mm; FWL: 38.0–42.4 mm; HWL: 36.7–40.9 mm.

##### Biological notes.

*Deutoleon lineatus lineatus* is a species that is observed very locally in calcareous grasslands of Chungcheongbuk-do and Gangwon-do in South Korea (Fig. 37F). Adults are mainly observed resting on grass stems during the daytime (Fig. 32A). Behavior that appeared to be territorial conflict against *Orthetrum* sp. was also observed. No attraction of the adults to light traps was observed at night; the species was active only during the day and at dusk. Adults emerge for a short period from late May to late June in South Korea. Larvae were not examined during this study, and nothing is known so far about the larvae of this species (Szőke 2021).

##### Distribution.

Korea, China, Russia, Moldova, Mongolia, Ukraine, Hungary, Kazakhstan, Kyrgyzstan (Krivokhatsky 2011; Szőke 2021)

##### Remarks.

*Deutoleon lineatus lineatus* is distributed in Europe to northern Asia. This species was first reported by Kuwayama (1924) from Korea. Another species in the same genus, *D. turanicus* Navás, is treated as a subspecies (Krivokhatsky 2011).

#### ﻿Genus Distoleon

Taxon classificationAnimaliaNeuropteraMyrmeleontidae

Banks, 1910

58208645-DE3E-50E3-BE4B-3D7BF4EBF344

Distoleon Banks, 1910: 42. Type species: Distoleon verticalis Banks, 1910. Type locality: Australia: Mid-Queensland.
Formicaleo Brauer, 1855: 719.
Eidoleon Esben-petersen, 1918: 15.
Salvaza Navás, 1917:12.
Feinerus Navás, 1919a: 190.
Nefeirus Navás, 1926: 103.
Dolicholeon Navás, 1929: 190.
Hyloleon Navás, 1929: 188.
Nasma Navás, 1930b: 409.
Feina Navás, 1931: 263.
Vessa Navás, 1931: 265.
Formileo Navás, 1933: 312.
Nima Navás, 1935: 53.

##### Diagnosis.

Adult. Medium to large sized antlions; forewing presectoral area usually with approximately 5–10 crossveins; forewing vein RP arising beyond CuA fork; forewing vein 2A fused with 3A; hindwing presectoral area with one crossvein; hindwing vein RP arising before MP fork; male without pilula axillaris; tibial spurs approximately as long as combined lengths of tarsomeres 1–3 in hindleg (Sekimoto 2014). Third instar larva. Mandibles equipped with three equidistant teeth; first pair of mesothoracic setiferous processes pedunculated, second pair sub-pedunculated; sternite VIII with odontoid processes; sternite IX with two rastra each with four digging setae (Badano and Pantaleoni 2014a).

##### Distribution.

Old world (Badano and Pantaleoni 2014a).

#### ﻿ Distoleon littoralis

Taxon classificationAnimaliaNeuropteraMyrmeleontidae

Miller & Stange, 1999

C89B726E-56A5-53AA-B6BF-27143C364692

**Figure 19.**
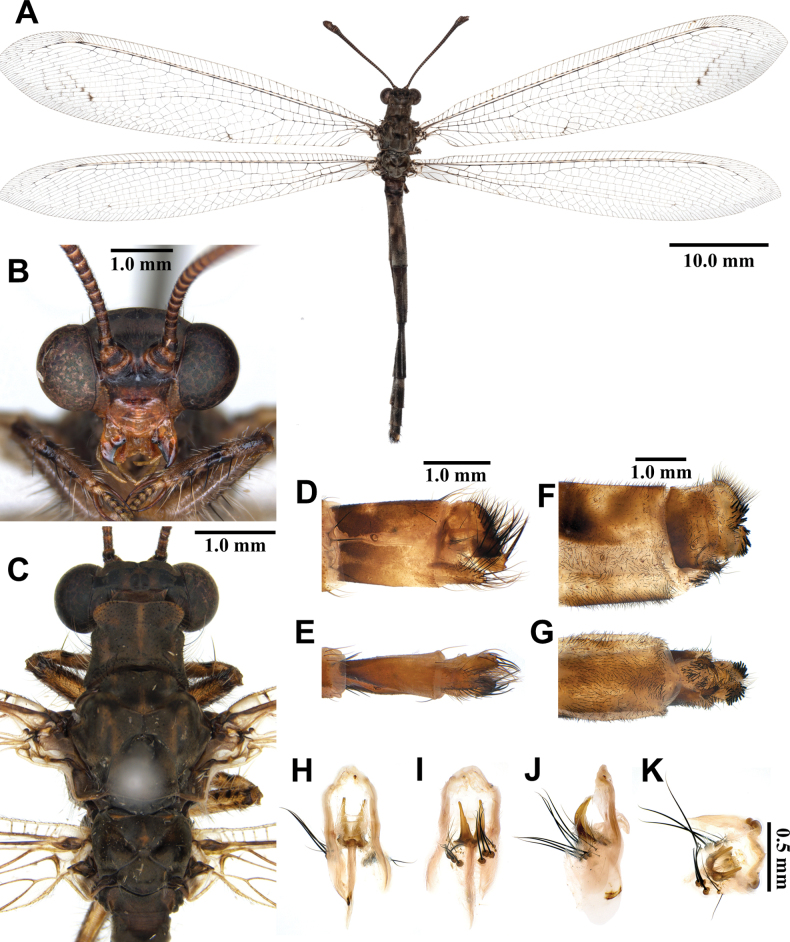
*Distoleon littoralis* Miller & Stange, 1999, adult. A. Dorsal habitus, male; B. Head, frontal view; C. Head and thorax, dorsal view; D, E. Male terminalia: D. Lateral view; E. Ventral view; F, G. Female terminalia: F. Lateral view; G. Ventral view; H–K. Male genitalia: H. Dorsal view; I. Ventral view; J. Lateral view; K. Caudal view.

**Figure 20.**
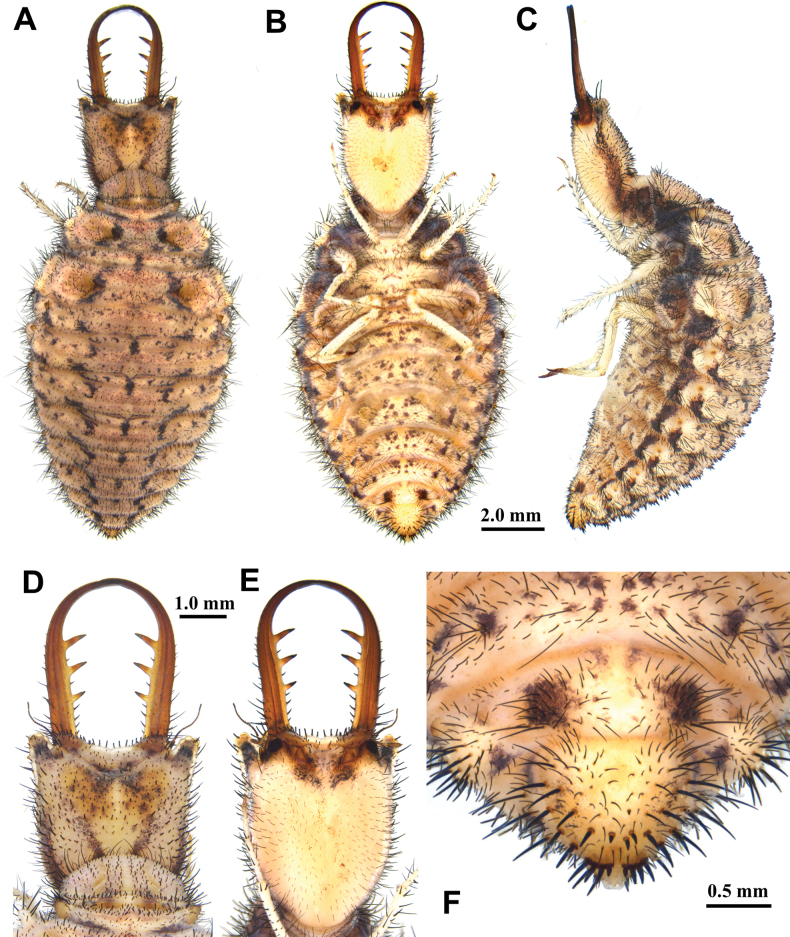
*Distoleon littoralis* Miller & Stange, 1999, third instar larva. A–C. Habitus: A. Dorsal view; B. Ventral view; C. Lateral view; D, E. Head: D. Dorsal view; E. Ventral view; F. Abdominal sternite IX.

Fig. 19, 20, 35H, 36G, 37D

Distoleon littoralis Miller & Stange, 1999: 53. Type locality: Taiwan: Ilan County: Hanben Beach.

##### Specimens examined.

[**JBNU**] • 1♀, Gyorae-ri, Jocheon-eup, Jeju-si, Jeju-do, Korea, 23.VII.2021, J.S. Kim; • 4♀, Sindu-ri, Wonbuk-myeon, Taean-gun, Chungcheongnam-do, Korea, 20.VIII.2022, J.S. Kim; • 1♂5♀ (reared from larva), Jungtong-ri, Bogil-myeon, Wando-gun, Jeollanam-do, Korea, 3.III.2023, J.I. Shim; • 1♂ (reared from larva), Sindu-ri, Wonbuk-myeon, Taean-gun, Chungcheongnam-do, Korea, 18.VI.2023, J.S. Kim; • 1♀, Sanghyo-dong, Seogwipo-si, Jeju-do, Korea, 23.IX.2023, J.S. Kim; • 2♂1♀, Gamsan-ri, Andeok-myeon, Seogwipo-si, Jeju-do, Korea, 20.VII.2024, Y.T. Jang; • 1♀, Jungdo-ri, Wando-eup, Wando-gun, Jeollanam-do, Korea, 26.VII.2024, J.S. Kim; • 2♀, Naewol-ri, Bigeum-myeon, Sinan-gun, Jeollanam-do, Korea, 27.VII.2024, M.K. Jeong; • 2♂2♀, Sindu-ri, Wonbuk-myeon, Taean-gun, Chungcheongnam-do, Korea, 31.VII.2024, J.S. Kim; • 1♀, Naewol-ri, Bigeum-myeon, Sinan-gun, Jeollanam-do, Korea, 1.VIII.2024, J.S. Kim; • 2♀, Jungdo-ri, Wando-eup, Wando-gun, Jeollanam-do, Korea, 4.VIII.2024, Y.T. Jang; • 2♀, Seopo-ri, Deokjeok-myeon, Ongjin-gun, Incheon, Korea, 13.VIII,.2024, J.S. Kim; • 2♂, Gureom-ri, Deokjeok-myeon, Ongjin-gun, Incheon, Korea, 14.VIII,.2024, J.S. Kim; • 1♂, Gureom-ri, Deokjeok-myeon, Ongjin-gun, Incheon, Korea, 15.VIII,.2024, J.S. Kim; • 1♂, Oksan-ri, Geoje-myeon, Geoje-si, Gyeongsangnam-do, Korea, 17.VIII,.2024, Y.T. Jang; • 1♂, Gilgok-ri, Maehwa-myeon, Uljin-gun, Gyeongsangbuk-do, Korea, 28.VIII.2024, J.S. Kim; • 2♀, Sa-ri, Heuksan-myeon, Sinan-gun, Jeollanam-do, Korea, 3.IX.2024, J.S. Kim; • 2 larvae (3^rd^ instar), Jungtong-ri, Bogil-myeon, Wando-gun, Jeollanam-do, Korea, 3.III.2023, J.I. Shim.

##### Diagnosis.

*Distoleon littoralis* has pale dark brown spots in the cubital area of the forewing and the rhegma area of the hindwing. Forewing presectoral area has eight or nine crossveins. Fore coxa has many elongate white hairs. In larvae, the head is yellowish white and has an anterior dark marking and a V-shaped small dark brown marking on the dorsal side, as well as two pairs of dark spots on the ventral side. Abdominal sternite IX has sparse short digging setae and a paired rastra each with four digging setae.

##### Description.

**Male, adult. *Head*** (Fig. 19B, C). Vertex slightly narrow, slightly raised, dark brown. Frons reddish brown, with broad dark brown band extending from below vertex to below base of antenna; clypeus yellowish brown, with long pale yellow hairs. Antenna reddish brown, long, with slightly defined club, densely covered with short black hairs; flagellum comprising ~50 flagellomeres, each flagellomere with distal yellow annulation. Mouthparts brown; labrum brown, with hyaline brown hairs; maxillary palpus yellowish brown; labial palpus yellowish brown.

***Thorax*** (Fig. 19C). Pronotum broad, length shorter than width, dark brown, with narrow yellow longitudinal middle stripe, with long black hairs. Mesonotum and metanotum dark brown, with several yellow spots.

***Legs*.** Coxae mostly yellow, moderately covered with long white hairs. Femora yellowish brown; moderately covered with black and white hairs. Tibiae mostly yellowish brown; partly black; densely covered with short black hairs. Tibial spurs dark brown proximally, reddish brown distally, long, curved, in forelegs and midlegs approximately as long as combined lengths of tarsomeres 1–4, in hindleg approximately as long as combined lengths of tarsomeres 1–3. Tarsi yellowish white, Tarsomere 5 as long as combined lengths of tarsomeres 1–4; claws reddish brown.

***Wings*** (Fig. 19A). With dark brown markings. Forewings veins and crossveins dark brown and pale yellow; presectoral area with eight or nine crossveins; RP arising beyond CuA fork; CuP supporting one cell before fusing with 1A; 2A fused with 3A; pterostigma yellowish white; cubital area with pale dark brown marking; anterior Banksian lines distinct; posterior Banksian lines distinct. Hindwing slightly shorter than forewing; presectoral area with one crossvein; RP arising before MP fork; pterostigma yellowish white; rhegma area with pale brown marking; pterostigma yellowish white; anterior Banksian lines absent; posterior Banksian lines absent; male without pilula axillaris.

***Abdomen*** (Fig. 19A). Shorter than hindwing, dark brown, tergites II– VIII with variable yellow markings, densely covered with short hyaline hairs.

***Genitalia*** (Fig. 19D, E, H–K). Ectoproct semicircular, covered with long black setae. Sternite IX narrow, covered with long black setae. Gonarcus white, arched. Mediuncus absent. Parameres well sclerotized, brown, with long black setae, moderately hooked in lateral view.

Size. BL: 29.9–33.3 mm; FWL: 30.5–34.8 mm; HWL: 28.0–34.1 mm.

**Female, adult.** Except terminalia, generally similar to male. Terminalia (Fig. 19F, G): tergite VIII wider than tergite IX; tergite IX narrow, rectangular in lateral view; ectoproct semicircular in lateral view; lateral gonapophyses semicircular in lateral view, smaller than ectoproct; posterior gonapophyses long, with long black setae; anterior gonapophyses absent; pregenital plate absent.

Size. BL: 27.8–33.1 mm; FWL: 29.8–38.2 mm; HWL: 28.5–37.4 mm.

**Larva, 3^rd^ instar.** General color reddish brown, with dark brown markings (Fig. 20A–C). Head longer than wide, with an anterior dark marking and a V-shaped small dark brown marking on dorsal side; with two pairs of dark spots on ventral side; with a dark brown stripe in lateral side; mandibles reddish brown; interdental mandibular setae (4) (1) (1) (0); external setae short (Fig. 20D, E). Abdominal sternite VIII with sparse black setae and prominent odontoid processes. Abdominal sternite IX with sparse short digging setae in front of rastra; a paired rastra each with four digging setae (Fig. 20F).

Size. BL: 12.1 mm; HL: 3.2 mm, HW: 2.6 mm, ML: 3.1 mm.

##### Biological notes.

*Distoleon littoralis* is frequently observed in South Korea, primarily in coastal areas, though it is also occasionally observed inland (Fig. 37D). Adults mainly emerge from July to September in South Korea. Larvae are ambush predators, concealing themselves in the sand of dunes. They were collected in sandy areas near the roots of *Pinus* trees (Fig. 36G).

##### Distribution.

Korea (new record), China (Stange et al. 2003; Wang et al. 2018).

##### Remarks.

The first record of *Distoleon littoralis* in Korea was by Okamoto (1926), who identified it as *D. contubernalis* (McLachlan, 1875). However, additional taxonomic research is needed on the species status of *D. littoralis* and its associated species complex.

#### ﻿ Distoleon nigricans

Taxon classificationAnimaliaNeuropteraMyrmeleontidae

(Okamoto, 1910)

553555B6-C1BE-588E-927D-4FB519F73AA2

Figs 21, 37D

Formicaleo nigricans Okamoto, 1910: 288. Type locality: Japan.
Formicaleon nigricans (Okamoto, 1910): Okamoto 1914: 249.
Distoleon nigricans (Okamoto, 1910): Kuwayama 1956: 30.

##### Specimens examined.

[**JBNU**] 1♂1♀, Oeseonmi-ri, Onjeong-myeon, Uljin-gun, Gyeongsangbuk-do, Korea, 9.VII.2022, J.S. Kim; • 1♀, Cheonggye-ri, Paldeok-myeon, Sunchang-gun, Jeonbuk-do, Korea, 21.VII.2022, J.I. Shim; • 1♀, Oeseonmi-ri, Onjeong-myeon, Uljin-gun, Gyeongsangbuk-do, Korea, 27.VII.2022, J.S. Kim; • 1♀, Sindu-ri, Wonbuk-myeon, Taean-gun, Chungcheongnam-do, Korea, 17.VI.2023, J.S. Kim; • 2♀, Yulji-ri, Susan-myeon, Jecheon-si, Chungcheongbuk-do, Korea, 24.VI.2023, J.S. Kim; • 2♀, Samjung-ri, Macheon-myeon, Hamyang-gun, Gyeongsangnam-do, Korea, 14.VII.2023, H. Han; • 1♀, Changwon-ri, Nam-myeon, Yeongwol-gun, Gangwon-do, Korea, 12.VI.2024, J.S. Kim; • 3♀, Yeokpyeong-ri, Daebyeong-myeon, Hapcheon-gun, Gyeongsangnam-do, Korea, 19.VI.2024, Y.T. Jang; • 2♀, Daegwang-ri, Sinseo-myeon, Yeoncheon-gun, Gyeonggi-do. 25.VII.2024, H. Han.

##### Diagnosis.

*Distoleon nigricans* has large, nearly circular, well-developed, dark brown spots in the cubital area of the forewing and the rhegma area of the hindwing. Pronotum has narrow yellow longitudinal middle stripe.

##### Description.

**Figure 21.**
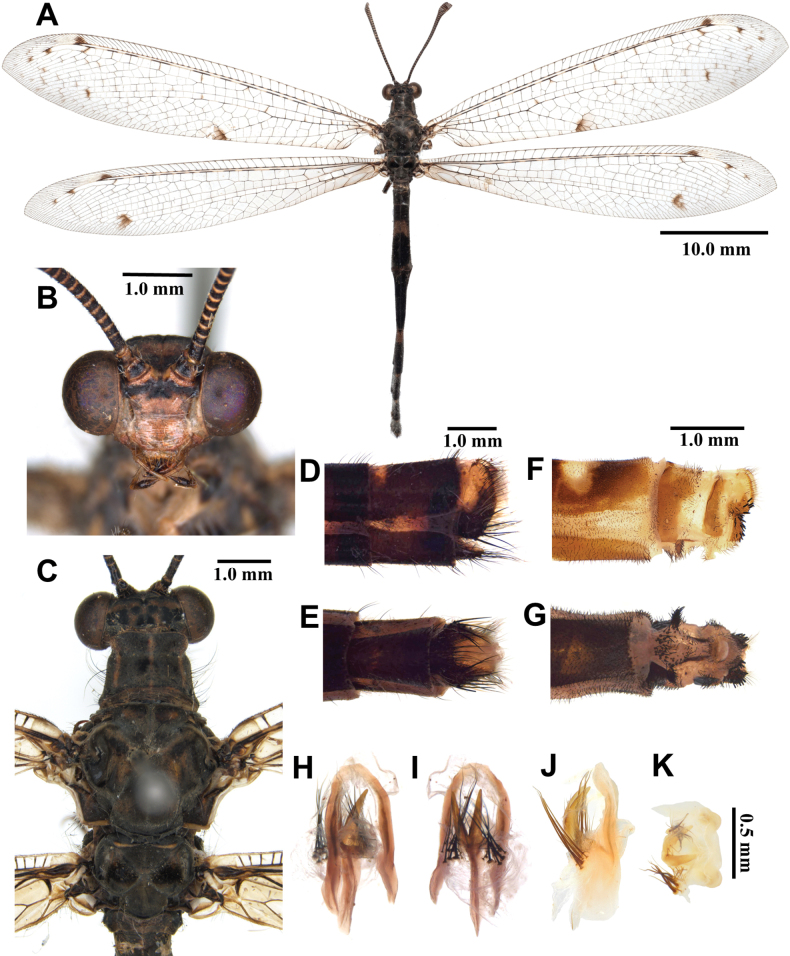
*Distoleon nigricans* (Okamoto, 1910), adult. A. Dorsal habitus, male; B. Head, frontal view; C. Head and thorax, dorsal view; D, E. Male terminalia: D. Lateral view; E. Ventral view; F, G. Female terminalia: F. Lateral view; G. Ventral view; H–K. Male genitalia: H. Dorsal view; I. Ventral view; J. Lateral view; K. Caudal view.

**Male, adult. *Head*** (Fig. 21B, C). Vertex slightly narrow, slightly raised, reddish brown. Frons reddish brown, with broad dark brown band extending from below vertex to below base of antenna; clypeus yellow, with long pale brown hairs. Antenna dark brown, long, with slightly defined club, densely covered with short black hairs; flagellum comprising ~45 flagellomeres, each flagellomere with distal yellow annulation. Mouthparts brown; labrum brown, with hyaline brown hairs; maxillary palpus brown; labial palpus brown, 3^rd^ labial palpomere dark brown.

***Thorax*** (Fig. 21C). Pronotum broad, length shorter than width, dark brown, with narrow yellow longitudinal middle stripe, with long black hairs. Mesonotum and metanotum dark brown, with several yellow spots.

***Legs*.** Coxae mostly dark brown, moderately covered with long white hairs. Femora mostly dark brown, partly brown; moderately covered with black and white hairs. Tibiae dark brown; moderately covered with black hairs. Tibial spurs brown proximally, reddish brown distally, long, curved, in forelegs and midlegs approximately as long as combined lengths of tarsomeres 1–4, in hindleg approximately as long as combined lengths of tarsomeres 1–3. Tarsi yellowish white, Tarsomere 5 longer than combined lengths of tarsomeres 1–4; claws reddish brown.

***Wings*** (Fig. 21A). With dark brown markings. Forewings veins and crossveins dark brown and pale yellow; presectoral area with 7–9 crossveins; RP arising beyond CuA fork; CuP supporting one cell before fusing with 1A; 2A fused with 3A at same time, connected by one crossvein; pterostigma yellowish white; cubital area with large dark brown marking; anterior Banksian lines distinct; posterior Banksian lines distinct. Hindwings shorter and narrower than forewings; presectoral area with one crossvein; RP arising before MP fork; pterostigma yellowish white; rhegma area with large dark brown marking; pterostigma yellowish white; anterior Banksian lines absent; posterior Banksian lines absent; male without pilula axillaris.

***Abdomen*** (Fig. 21A). Shorter than hindwing, dark brown, tergites II–VIII with variable yellow markings, densely covered with short black hairs.

***Genitalia*** (Fig. 21D, E, H–K). Ectoproct semicircular, covered with long black setae. Sternite IX narrow, covered with long black setae. Gonarcus reddish brown, arched. Mediuncus absent. Parameres well sclerotized, reddish brown, with long black setae, moderately hooked in lateral view.

Size. BL: 32.4 mm; FWL: 33.9 mm; HWL: 31.9 mm.

**Female, adult.** Except terminalia, generally similar to male. Terminalia (Fig. 21F, G): tergite VIII wider than tergite IX; tergite IX narrow, rectangular in lateral view; ectoproct semicircular in lateral view; lateral gonapophyses semicircular in lateral view, smaller than ectoproct; posterior gonapophyses short, with long black setae; anterior gonapophyses absent; pregenital plate absent.

Size. BL: 33.2–36.1 mm; FWL: 36.9–43.2 mm; HWL: 34.8–39.9 mm.

##### Biological notes.

*Distoleon nigricans* is a species that is observed in South Korea, primarily in inland areas across grasslands and mountainous topography (Fig. 37D). Adults emerge from June in South Korea. Larvae are known to be ambush hunters. They were not found during this study; for details on the larval ecology, refer to Ikeda and Okui (2017).

##### Distribution.

Korea, Japan, China (Wang et al. 2018).

##### Remarks.

Okamoto (1926) first reported *Distoleon nigrigans* from Korea, misidentifying it as *D. tetragrammicus*, a widespread species in the western Palaearctic, only avoiding cold climates and true deserts (Badano and Pantaleoni 2014a). In contrast, *D. nigricans* has only been reported to be distributed in northeast Asia (Sekimoto 2014; Wang et al. 2018).

### ﻿Tribe Megistopini Navás, 1912

#### ﻿Genus Paraglenurus

Taxon classificationAnimaliaNeuropteraMyrmeleontidae

van der Weele, 1909

DA862E87-2B2A-52D2-A88E-8471D32EFCBC

Paraglenurus van der Weele, 1909: 29. Type species: Myrmeleon scopifer Gerstaecker, 1887. Type locality: Indonesia: Seram.
Glenuroides Okamoto, 1910: 294.
Eoleon Navás, 1921: 65.

##### Diagnosis.

Adult. Antenna longer than head plus thoracic length.; eye large, nearly as wide as frons; leg slender, hind femur plus tibia nearly as long as entire length of head plus thorax; pretarsal claw opposable; male paramere curved slender plate-like; mediuncus prominent; female tergite VII with some thick setae on posterior margin; posterior gonapophyses elongate, digitiform; lateral gonapophyses covered with thick digging setae. Third instar larva. Distance between base of mandible and first tooth shorter than that between first and third teeth; third tooth larger than second tooth; abdominal spiracles developed, prominent; abdominal sternite VIII without odontoid processes; rastra of abdominal sternite IX with inner digging seta shorter by 1/3 than the others (Zheng and Liu 2025).

##### Distribution.

Asia (China, Indonesia, Japan, Korea, Russia (Far east), Vietnam), Africa (Madagascar, Seychelles) (Sekimoto 2014; Ábrahám 2023; Zheng and Liu 2025)

#### ﻿ Paraglenurus albiventris

Taxon classificationAnimaliaNeuropteraMyrmeleontidae

Matsumoto, Kikuta & Hayashi, 2021

BDC5E412-785E-5B96-9903-2684B165A8F1

Figs 22, 30, 37E

Paraglenurus albiventris Matsumoto, Kikuta & Hayashi, 2021: 19. Type locality. Japan: Nara: Soni-mura: Kameyama: Taroji.

##### Specimens examined.

[**JBNU**] • 1♂, Oeseonmi-ri, Onjeong-myeon, Uljin-gun, Gyeongsangbuk-do, Korea, 27.VII.2022, J.S. Kim; • 1♀, Samjung-ri, Macheon-myeon, Hamyang-gun, Gyeongsangnam-do, Korea, 17.IX.2022, J.S. Kim; • 4♀, Sindu-ri, Wonbuk-myeon, Taean-gun, Chungcheongnam-do, Korea, 17.VI.2023, J.S. Kim; • 1♀, Yulji-ri, Susan-myeon, Jecheon-si, Chungcheongbuk-do, 24.VI.2023, J.S. Kim; • 2♀, Woro-ri, Ugok-myeon, Goryeong-gun, Gyeongsangbuk-do, Korea, 22.VI.2024, Y.T. Jang; • 1♀, Sangwon-ri, Gachang-myeon, Dalseong-gun, Daegu, Korea, 23.VI.2024, Y.T. Jang; • 2♀, Cheonbu-ri, Buk-myeon, Ulleung-gun, Gyeongsangbuk-do, Korea, 10.VII.2024, J.S. Kim; • 1♀, Samgot-ri, Jung-myeon, Yeoncheon-gun, Gyeonggi-do, Korea, 11.VII.2024, Y.T. Jang; • 2♂, Nambu-ri, Inje-eup, Inje-gun, Gangwon-do, Korea, 28.VII.2024, Y.T. Jang; • 3♀, Yulji-ri, Susan-myeon, Jecheon-si, Chungcheongbuk-do, Korea, 30.VII.2024, J.S. Kim; • 1♀, Gureom-ri, Deokjeok-myeon, Ongjin-gun, Incheon, Korea, 15.VIII.2024, J.S. Kim.

##### Diagnosis.

Compared to other species in the genus *Paraglenurus*, *P. albiventris* has the morphological characteristics of largely whitish tergites II–V in the male, a usually weak preapical dark brown marking on the hindwing, and a vertex that is very wide and well-developed.

##### Description.

**Figure 22.**
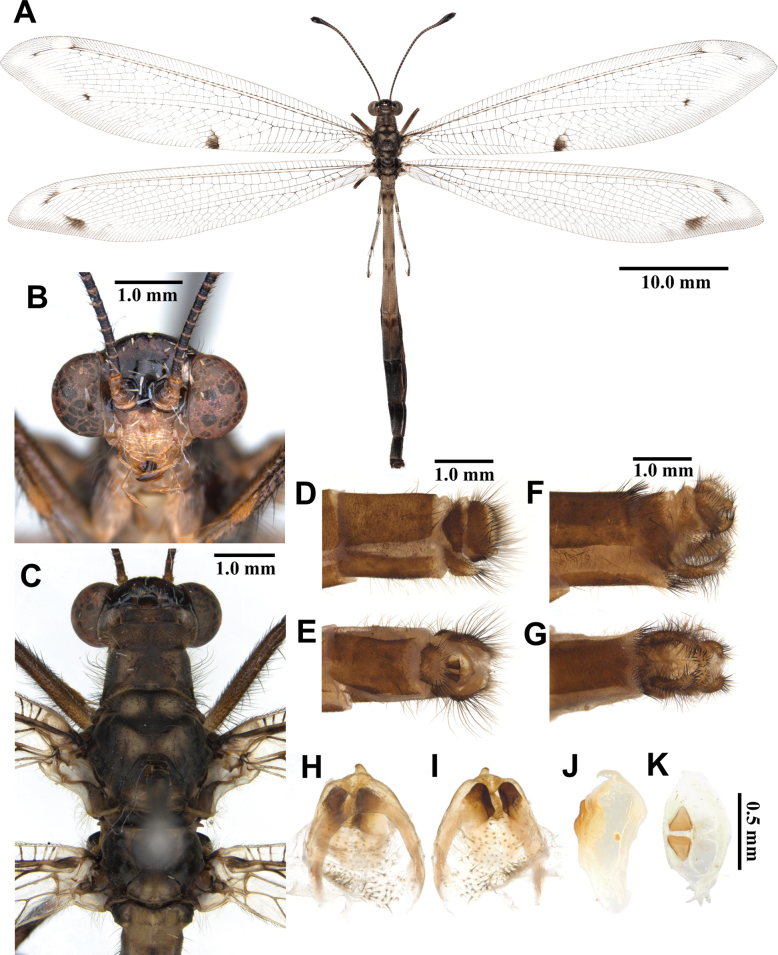
*Paraglenurus albiventris* Matsumoto, Kikuta & Hayashi, 2021, adult. A. Dorsal habitus, male; B. Head, frontal view; C. Head and thorax, dorsal view; D, E. Male terminalia: D. Lateral view; E. Ventral view; F, G. Female terminalia: F. Lateral view; G. Ventral view; H–K. Male genitalia: H. Dorsal view; I. Ventral view; J. Lateral view; K. Caudal view.

**Male, adult. *Head*** (Fig. 22B, C). Vertex wide, strongly raised, reddish brown. Frons yellow, with broad dark brown band extending from below vertex to below base of antenna; clypeus yellow, with long black hairs. Antenna dark brown, long, with slightly defined club, densely covered with short black hairs; flagellum comprising ~42 flagellomeres, each flagellomere with distal yellow annulation. Mouthparts yellowish white; labrum yellowish white, with hyaline yellow hairs; maxillary palpus yellow; labial palpus yellow, spindle-shaped.

***Thorax*** (Fig. 22C). Pronotum slender, longer than broad, brown, with long brown hairs. Mesonotum and metanotum dark brown, with yellow portions.

***Legs*.** Coxae yellow, moderately covered with yellow hairs. Femora mostly dark brown, partly brown; moderately covered with black hairs. Tibiae dark brown; moderately covered with black hairs. Tibial spurs dark yellowish brown proximally, reddish brown distally, slightly long, slightly curved, approximately as long as tarsomere 1. Tarsi yellowish brown, tarsomere 5 as long as tarsomere 1; claws brown.

***Wings*** (Fig. 22A). With white and dark brown markings. Forewings veins and crossveins dark brown; presectoral area with 10 or 11 pterostigma yellowish white; RP arising beyond CuA fork; CuP supporting one cell before fusing with 1A; 2A fused with 3A; pterostigma white; anterior Banksian lines absent; posterior Banksian lines absent. Hindwing slightly shorter and narrower than forewing; presectoral area with one crossvein; RP arising before MP fork; pterostigma white; anterior Banksian lines absent; posterior Banksian lines absent; male without pilula axillaris.

***Abdomen*** (Fig. 22A). Shorter than hindwing, yellowish brown, densely covered with pale brown hairs; tergite II–V largely yellowish white.

***Genitalia*** (Fig. 22D, E, H–K). Ectoproct semicircular, with long black setae. Sternite IX narrow, covered with long black setae. Gonarcus brown, arched. Mediuncus lightly sclerotized, brown, moderately hooked in lateral view. Parameres well sclerotized, dark brown, triangular in caudal view.

Size. BL: 31.7–34.7 mm; FWL: 32.7–34.6 mm; HWL: 32.5–33.3 mm.

**Female, adult.** General morphology, except coloration of abdomen and terminalia, almost as in male. Abdomen: dark brown, tergite II–VII each with median triangular pale marking at posterior end; tergites III–V each with pair of large pale spot medially (Fig. 25). Terminalia (Fig. 22F, G): tergite VIII wider than tergite IX; tergite IX narrow, triangular in lateral view; ectoproct semicircular in lateral view; lateral gonapophyses semicircular in lateral view, smaller than ectoproct; posterior gonapophyses long, curved, with long black setae; anterior gonapophyses absent; pregenital distinct, plate triangular, presented on membrane below tergite VIII.

Size. BL: 25.8–31.1 mm; FWL: 29.4–36.2 mm; HWL: 28.3–35.2 mm.

##### Biological notes.

*Paraglenurus albiventris* is a species frequently observed in open environments, such as coastal dunes and inland grasslands, and is distributed throughout South Korea (Fig. 37E). Adults emerge earlier than other *Paraglenurus* species in Korea, appearing from June. Larvae are known to be ambush hunters. They were not examined during this study; for details on their ecology, refer to Matsumoto et al. (2021).

##### Distribution.

Korea (new record), Japan (Matsumoto et al. 2021).

##### Remarks.

*Paraglenurus albiventris* was described by Matsumoto et al. (2021) based on specimens from Japan. A taxonomic review of the genus *Paraglenurus* is needed in countries where it has been previously recorded.

#### ﻿ Paraglenurus japonicus

Taxon classificationAnimaliaNeuropteraMyrmeleontidae

(McLachlan, 1867)

130B6329-8FC8-5220-B86F-2BDCBC02427F

Figs 23, 37E

Glenurus japonicus McLachlan, 1867: 248. Type locality. Japan
Glenuroides communis Okamoto, 1910: 295. Type locality: Japan: Nakano.
Eoleon japonicus (McLachlan, 1867): Navás 1921: 66.
Paraglenurus japonicus (McLachlan, 1867): Miller and Stange 1999: 60.
Paraglenurus littoralis Miller & Stange, 1999: 56. Type locality: Taiwan: Ilan County: Hanben Beach.
Paraglenurus riparius Miller & Stange, 1999: 59. Type locality: Taiwan: Ilan County: Yinshih Bridge.

##### Specimens examined.

[**JBNU**] • 1♂, Daegok-ri, Janggye-myeon, Jangsu-gun, Jeonbuk-do, Korea, 14.VII.2022, J.S. Kim; • 2♀, Sindu-ri, Wonbuk-myeon, Taean-gun, Chungcheongnam-do, Korea, 20.VIII.2022, J.S. Kim; • 1♂1♀, Oeseonmi-ri, Onjeong-myeon, Uljin-gun, Gyeongsangbuk-do, Korea, 27.VII.2022, J.S. Kim; • 1♂4♀, Samjung-ri, Macheon-myeon, Hamyang-gun, Gyeongsangnam-do, Korea, 14.VII.2023, H. Han; • 1♂12♀, Yulji-ri, Susan-myeon, Jecheon-si, Chungcheongbuk-do, Korea, 30.VII.2024, J.S. Kim; • 7♂9♀, Sindu-ri, Wonbuk-myeon, Taean-gun, Chungcheongnam-do, Korea, 31.VII.2024, J.S. Kim; • 1♀, Seopo-ri, Deokjeok-myeon, Ongjin-gun, Incheon, Korea, 13.VIII.2024, J.S. Kim; • 2♂4♀, Gureom-ri, Deokjeok-myeon, Ongjin-gun, Incheon, Korea, 14.VIII.2024, J.S. Kim; • 4♂11♀, Gureom-ri, Deokjeok-myeon, Ongjin-gun, Incheon, Korea, 15.VIII.2024, J.S. Kim; • 1♂, Gancheok-ri, Gandong-myeon, Hwacheon-gun, Gangwon-do, Korea, 23.VIII.2024, J.S. Kim; • 1♂, Gilgok-ri, Maehwa-myeon, Uljin-gun, Gyeongsangbuk-do, Korea, 28.VIII.2024, J.S. Kim; • 1♂3♀, Ye-ri, Heuksan-myeon, Sinan-gun, Jeollanam-do, Korea, 2.IX.2024, J.S. Kim; • 1♂, Gilgok-ri, Maehwa-myeon, Uljin-gun, Gyeongsangbuk-do, Korea, 7.IX.2024, J.S. Kim; • 1♂, Dae-ri, Yeonghae-myeon, Yeongdeok-gun, Gyeongsangbuk-do, Korea, 20.VII.2024, H. Han.

##### Diagnosis.

Compared to other species in the genus *Paraglenurus*, *P. japonicus* has the morphological characteristics of the apex of each flagellum being slightly pale yellow starting from the apical ~1/4 of the antenna, with a usually distinct preapical dark brown marking on the hindwing and an adjacent white marking that is indistinct and oval-shaped.

##### Description.

**Figure 23.**
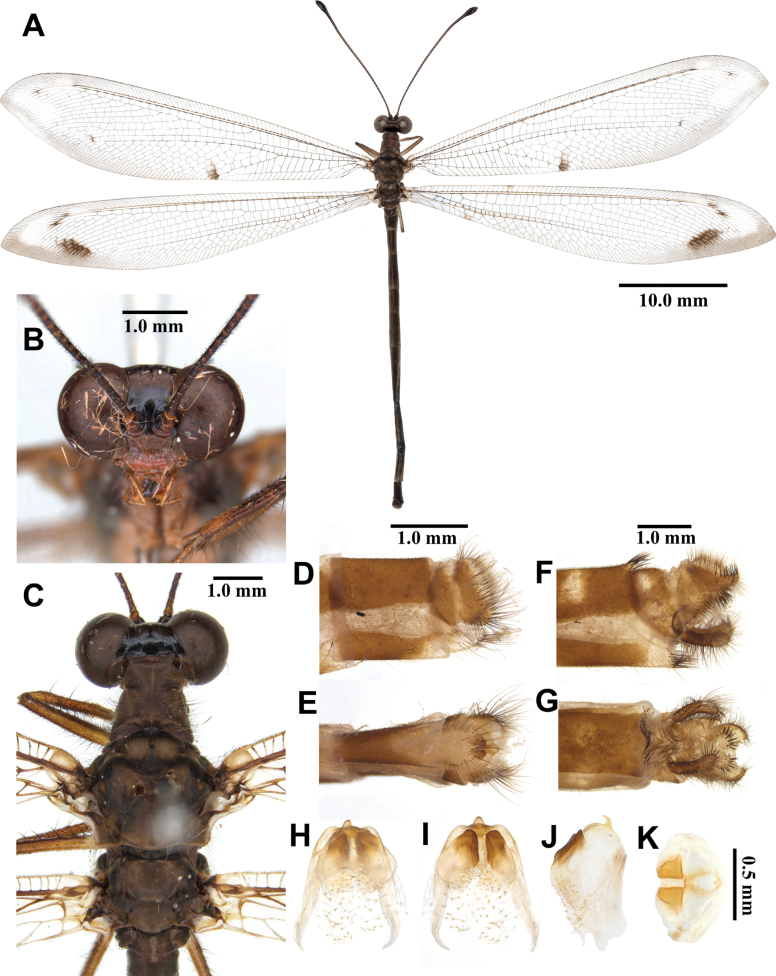
*Paraglenurus japonicus* (McLachlan, 1867), adult. A. Dorsal habitus, male; B. Head, frontal view; C. Head and thorax, dorsal view; D, E. Male terminalia: D. Lateral view; E. Ventral view; F, G. Female terminalia: F. Lateral view; G. Ventral view; H, I. Male genitalia: H. Dorsal view; I. Lateral view.

**Male, adult. *Head*** (Fig. 23B, C). Vertex narrow, weakly raised, reddish brown. Frons yellowish brown, with broad dark brown band extending from below vertex to below base of antenna; clypeus yellow, with long black hairs. Antenna dark brown, long, with slightly defined club, densely covered with short black hairs; flagellum comprising ~45 flagellomeres, each flagellomere with distal yellow annulation. Mouthparts reddish brown; labrum reddish brown, with hyaline brown hairs; maxillary palpus yellowish brown; labial palpus yellowish brown, spindle-shaped.

***Thorax*** (Fig. 23C). Pronotum slender, longer than broad, brown, with long brown hairs. Mesonotum and metanotum dark brown, with yellow portions.

***Legs*.** Coxae yellow, moderately covered with yellow hairs. Femora mostly dark brown, partly brown; moderately covered with black hairs. Tibiae dark brown; moderately covered with black hairs. Tibial spurs dark brown, slightly long, slightly curved, approximately as long as tarsomere 1. Tarsi yellowish brown, tarsomere 5 slightly longer than tarsomere 1; claws brown.

***Wings*** (Fig. 23A). With white and dark brown markings. Forewings veins and crossveins dark brown; presectoral area with 10 or 11 crossveins; RP arising beyond CuA fork; CuP supporting one cell before fusing with 1A; 2A fused with 3A fused; pterostigma white; anterior Banksian lines absent; posterior Banksian lines absent. Hindwing slightly longer and narrower than forewing; presectoral area with one crossvein; RP arising before MP fork; pterostigma white; anterior Banksian lines absent; posterior Banksian lines absent; male without pilula axillaris.

***Abdomen*** (Fig. 23A). Shorter than hindwing, dark brown, posterior margin of tergites II–VII bordered with yellow, tergites III–V sometimes with median yellow marking, densely covered with brown hairs.

***Genitalia*** (Fig. 23D, E, H–K). Ectoproct semicircular, covered with long black setae. Sternite IX narrow, covered with long black setae. Gonarcus brown, arched. Mediuncus brown, lightly sclerotized, lightly hooked in lateral view. Parameres well sclerotized, reddish brown, triangular in caudal view.

Size. BL: 27.3–38.5 mm; FWL: 29.8–37.3 mm; HWL: 30.1–37.4 mm.

**Female, adult.** General morphology, except head and terminalia, almost as in male. Head: vertex slightly narrow, strongly raised. Terminalia (Fig. 23F, G): tergite VIII wider than tergite IX; tergite IX narrow, triangular in lateral view; ectoproct semicircular in lateral view; lateral gonapophyses semicircular in lateral view, smaller than ectoproct; posterior gonapophyses long, curved, with long black setae; anterior gonapophyses absent; pregenital distinct, plate triangular, presented on membrane below tergite VIII.

Size. BL: 27.5–31.2 mm; FWL: 31.7–39.4 mm; HWL: 31.9–40.9 mm.

##### Biological notes.

*Paraglenurus japonicus* is a species that is commonly observed throughout South Korea. It is observed in various environments, from coastal dunes to inland grasslands and mountains (Fig. 37E). Adults emerge from July to September in South Korea. Larva are known to be ambush hunters but were not examined during this study; for details on their ecology, refer to Matsumoto et al. (2021).

##### Distribution.

Korea, Japan, Taiwan, Russia. However, their *Paraglenurus japonicus* includes multiple species described in past studies; therefore, past distributional records outside Korea and Japan need to be confirmed (Matsumoto et al. 2021).

##### Remarks.

*Paraglenurus japonicus* is a species with large morphological variation in size and the pattern of its wing markings.

#### ﻿ Paraglenurus melanostictus

Taxon classificationAnimaliaNeuropteraMyrmeleontidae

Matsumoto, Kikuta & Hayashi, 2021

B27B317B-807D-56A0-A73A-F30DCAC8A207

Figs 24, 37E

Paraglenurus melanostictus Matsumoto, Kikuta & Hayashi, 2021: 21. Type locality. Japan: Nara: Yamatokuriyama-shi: Shinmachi.

##### Specimens examined.

[**JBNU**] • 1♀, Daegok-ri, Janggye-myeon, Jangsu-gun, Jeonbuk-do, Korea, 14.VII.2022, J.S. Kim; • 2♀, Oeseonmi-ri, Onjeong-myeon, Uljin-gun, Gyeongsangbuk-do, Korea, 27.VII.2022, J.S. Kim; • 3♂14♀, Samjung-ri, Macheon-myeon, Hamyang-gun, Gyeongsangnam-do, Korea, 14.VII.2023, H. Han; • 6♀, Gilgok-ri, Maehwa-myeon, Uljin-gun, Gyeongsangbuk-do, Korea, 20.VII.2023, DB Choi; • 1♂2♀, Gilgok-ri, Maehwa-myeon, Uljin-gun, Gyeongsangbuk-do, Korea, 27.VII.2023, H. Han; • 7♂5♀, Oeseonmi-ri, Onjeong-myeon, Uljin-gun, Gyeongsangbuk-do, Korea, 13.VII.2024, J.S. Kim; • 2♂2♀, Yulji-ri, Susan-myeon, Jecheon-si, Chungcheongbuk-do, Korea, 30.VII.2024, J.S. Kim; • 4♀, Gancheok-ri, Gandong-myeon, Hwacheon-gun, Gangwon-do, Korea, 23.VIII.2024, J.S. Kim; • 1♀, Gilgok-ri, Maehwa-myeon, Uljin-gun, Gyeongsangbuk-do, Korea, 28.VIII.2024, J.S. Kim; • 4♂1♀, Gilgok-ri, Maehwa-myeon, Uljin-gun, Gyeongsangbuk-do, Korea, 7.IX.2024, J.S. Kim; • 1♂2♀, Dae-ri, Yeonghae-myeon, Yeongdeok-gun, Gyeongsangbuk-do, Korea, 20.VII.2024, H. Han.

##### Diagnosis.

Compared to other species in the genus *Paraglenurus*, *P. melanostictus* has the morphological characteristics of the apical 1/3–1/2 of each flagellum being pale yellow starting from the apical ~1/4 of the antenna, a very distinct preapical dark brown marking on the hindwing, and an adjacent white marking is very distinct and rounded.

##### Description.

**Figure 24.**
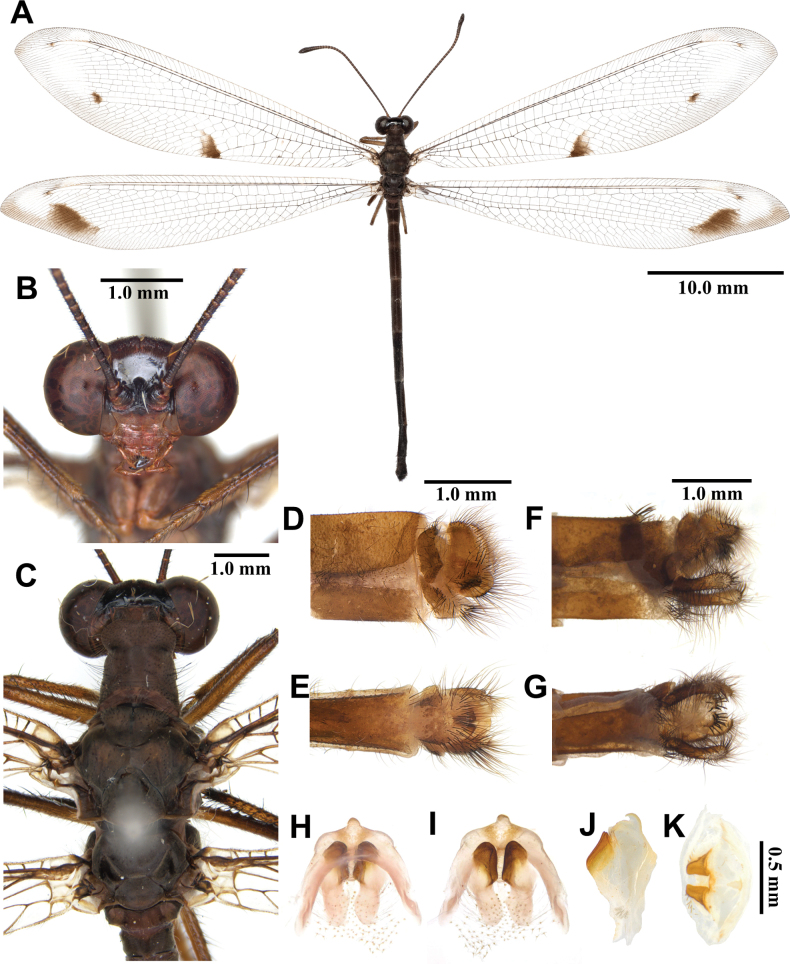
*Paraglenurus melanostictus* Matsumoto, Kikuta & Hayashi, 2021, adult. A. Dorsal habitus, male; B. Head, frontal view; C. Head and thorax, dorsal view; D, E. Male terminalia: D. Lateral view; E. Ventral view; F, G. Female terminalia: F. Lateral view; G. Ventral view; H–K. Male genitalia: H. Dorsal view; I. Ventral view; J. Lateral view; K. Caudal view.

**Male, adult. *Head*** (Fig. 24B, C). Vertex slightly narrow, moderately raised, reddish brown. Frons yellowish brown, with broad dark brown band extending from below vertex to below base of antenna; clypeus yellow, with long black hairs. Antenna dark brown, long, with slightly defined club, densely covered with short black hairs; flagellum comprising ~44 flagellomeres, each flagellomere with distinct distal yellow annulation. Mouthparts reddish brown; labrum reddish brown, with hyaline brown hairs; maxillary palpus yellowish brown; labial palpus yellowish brown, spindle-shaped.

***Thorax*** (Fig. 24C). Pronotum slender, longer than broad, brown, with long brown hairs. Mesonotum and metanotum dark brown, covered with hyaline hairs.

***Legs*.** Coxae yellow, moderately covered with yellow hairs. Femora mostly dark brown, partly brown; moderately covered with black hairs. Tibiae dark brown; moderately covered with black hairs. Tibial spurs dark brown, slightly long, slightly curved, approximately as long as tarsomere 1. Tarsi yellowish brown, tarsomere 5 slightly longer than tarsomere 1; claws brown.

***Wings*** (Fig. 24A). With white and dark brown markings. Forewing veins and crossveins dark brown; presectoral area with 11 or 12 crossveins; RP arising beyond CuA fork; CuP supporting one cell before fusing with 1A; 2A fused with 3A; pterostigma white; anterior Banksian lines absent; posterior Banksian lines absent. Hindwing slightly longer and narrower than forewing; presectoral area with one crossvein; RP arising before MP fork; pterostigma white; anterior Banksian lines absent; posterior Banksian lines absent; male without pilula axillaris.

***Abdomen*** (Fig. 24A). Shorter than hindwing, dark brown, posterior margin of tergites II–VII bordered with yellow, tergites III–V sometimes with median yellow marking, densely covered with brown hairs.

***Genitalia*** (Fig. 24D, E, H–K). Ectoproct semicircular, covered with long black setae. Sternite IX narrow, covered with long black setae. Gonarcus reddish brown, arched. Mediuncus lightly sclerotized, lightly hooked in lateral view. Parameres well sclerotized, dark brown, triangular in caudal view.

Size. BL: 24.5–32.4 mm; FWL: 26.4–32.4 mm; HWL: 25.5–30.0 mm.

**Female, adult.** General morphology, except head and terminalia, almost as in male. Head: vertex slightly wide, strongly raised. Terminalia (Fig. 24F, G): tergite VIII wider than tergite IX; tergite IX narrow, triangular in lateral view; ectoproct triangular in lateral view; lateral gonapophyses semicircular in lateral view, smaller than ectoproct; posterior gonapophyses long, curved, with long black setae; anterior gonapophyses absent; pregenital distinct, plate triangular, presented on membrane below tergite VIII.

Size. BL: 22.8–26.4 mm; FWL: 29.4–33.5 mm; HWL: 29.3–33.3 mm.

##### Biological notes.

*Paraglenurus melanostictus* is a species that is mainly observed in mountainous regions throughout South Korea (Fig. 37E). Adults emerge from July to September in South Korea. Larvae are known to be ambush hunters. They were not examined during this study; for details on their ecology, refer to Matsumoto et al. (2021).

##### Distribution.

Korea (new record), Japan (Matsumoto et al. 2021).

##### Remarks.

*Paraglenurus melanostictus* was described as new based on specimens from Japan. A taxonomic review of the genus *Paraglenurus* is needed in countries where this species has been previously recorded.

### ﻿Subfamily Ascalaphinae Lefèbvre, 1842

**Tribe Ascalaphini Lefèbvre, 1842**

#### ﻿Genus Ascalohybris

Taxon classificationAnimaliaNeuropteraMyrmeleontidae

Sziráki, 1998

B66596DF-EA24-59A7-A996-0D03BABE682A

Ascalohybris Sziráki, 1998: 59. Type species: Ascalaphus javanus Brumeister, 1839. Type locality: Indonesia: Java.
Hybris Lefèbvre, 1842: 6.

##### Diagnosis.

Adult. Antennae without hairs, as long as forewing or at least reaching pterostigma, basal half of the male antenna curved; compound eyes with upper and lower parts subequal in size; abdomen long, cylindrical, without hairs; in both sexes, abdomen shorter than hindwing, about 2/3 its length; male ectoproct process long and forcipate (Wang et al. 2018).

##### Distribution.

Asia (Wang et al. 2018).

#### ﻿ Ascalohybris subjacens

Taxon classificationAnimaliaNeuropteraMyrmeleontidae

(Walker, 1853)

565B3E92-894C-5928-8182-CDC6B3F93FDB

**Figure 25.**
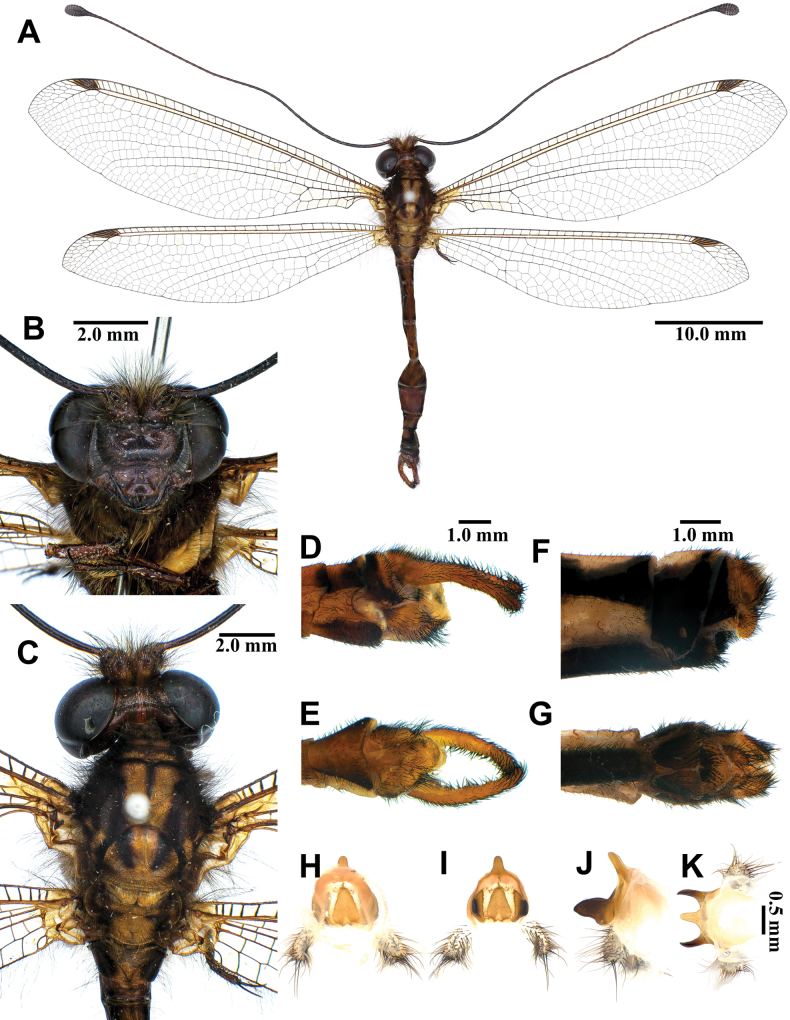
*Ascalohybris subjacens* (Walker, 1853), 2021, adult. A. Dorsal habitus, male; B. Head, frontal view; C. Head and thorax, dorsal view; D, E. Male terminalia: D. Lateral view; E. Ventral view; F, G. Female terminalia: F. Lateral view; G. Ventral view; H–K. Male genitalia: H. Dorsal view; I. Ventral view; J. Lateral view; K. Caudal view.

**Figure 26.**
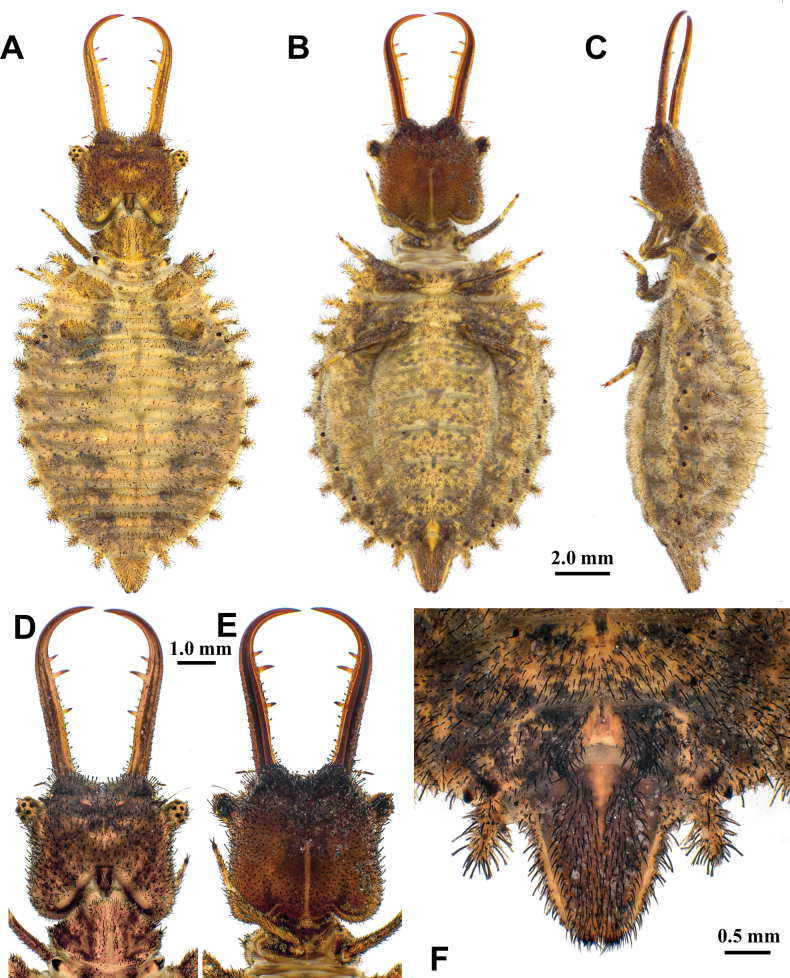
*Ascalohybris subjacens* (Walker, 1853), third instar larva. A–C. Habitus: A. Dorsal view; B. Ventral view; C. Lateral view; D, E. Head: D. Dorsal view; E. Ventral view; F. Abdominal sternite IX.

Figs 25, 26, 33B, 34B, 35I, 36H, 37G

Ascalaphus subjacens Walker, 1853: 431. Type locality: China.
Ascalaphus remotus Walker, 1853: 447. Type locality: China.
Hybris subjacens (Walker, 1853): McLachlan 1871: 267.
Glyptobasis brunnea Esben-Petersen, 1913: 224. Type locality: Taiwan: Banshoryo-district: Sokutsu.
Ascalohybris subjacens (Walker, 1853): Sziráki 1998: 59.

##### Specimens examined.

[**JBNU**] • 1♂, Samdu-ri, Gunoe-myeon, Wando-gun, Jeollanam-do, Korea, 10.VII.2023, D.K. Ra; • 1♂, Gilgok-ri, Maehwa-myeon, Uljin-gun, Gyeongsangbuk-do, Korea, 27.VII.2023, H. Han; • 1♂1♀, Jungdo-ri, Wando-eup, Wando-gun, Jeollanam-do, Korea, 26.VII.2024, J.S. Kim; 12♂5♀, Naewol-ri, Bigeum-myeon, Sinan-gun, Jeollanam-do, Korea, 26.VII.2024, M.K. Jeong; • 2♂1♀, Yulji-ri, Susan-myeon, Jecheon-si, Chungcheongbuk-do, Korea, 30.VII.2024, J.S. Kim; • 1♂, Naewol-ri, Bigeum-myeon, Sinan-gun, Jeollanam-do, Korea, 1.VIII.2024, J.S. Kim; 1♀, Gwangdae-ri, Bigeum-myeon, Sinan-gun, Jeollanam-do, Korea, 1.VIII.2024, J.S. Kim; • 1♂1♀, Gureom-ri, Deokjeok-myeon, Ongjin-gun, Incheon, Korea, 14.VIII.2024, J.S. Kim; • 1♂, Ye-ri, Heuksan-myeon, Sinan-gun, Jeollanam-do, Korea, 2.IX.2024, J.S. Kim; 1♀, Sa-ri, Heuksan-myeon, Sinan-gun, Jeollanam-do, Korea, 3.IX.2024, J.S. Kim; 1 larva (3^rd^ instar), Seolgye-ri, Yeongdong-eup, Yeongdong-gun, Chungcheongbuk-do, Korea, 14.IV.2024, J.S. Kim.

##### Diagnosis.

Frons and gena are dark brown. Antenna is long and reaches pterostigma. In lateral view, mesonotum has a broad yellow stripe medially. Male ectoprocts are elongated, forcipate, and covered with long black setae; their length is longer than 4 × of width. In larvae, head is quadrate and approximately as long as it is wide. Dorsal side of the head capsule is brown with some yellow markings, and the mandibles are brown. Dorsal side of the abdominal tergites has a dark brown median longitudinal stripe.

##### Description.

**Male, adult. *Head*** (Fig. 25B, C) Vertex slightly narrow, moderately depressed, dark brown, with sparse long dark brown hairs. Frons dark brown, with sparse long black hairs; clypeus dark brown, with sparse black hairs. Eye with a transverse furrow. Antenna dark brown, considerably long, with strongly defined club; flagellum comprising ~60 flagellomeres. Mouthparts dark brown; labrum dark brown, with black hairs; maxillary palpus yellowish brown; labial palpus yellowish brown.

***Thorax*** (Fig. 25C). Pronotum narrow, considerably shorter than width, dark brown, with longitudinal yellow stripe, moderately covered with long brown hairs. Mesonotum and metanotum generally dark brown, moderately covered with long brown hairs. Mesonotum medially with a broad yellow stripe in lateral view.

***Legs*.** Coxae reddish brown, moderately covered with black setae. Femora reddish brown, moderately covered with black setae. Tibiae reddish brown, covered with sparse black setae. Tibial spurs black, slightly long, slightly curved, approximately as long as combined lengths of tarsomeres 1–3. Tarsi reddish brown, tarsomere 5 approximately as long as combined lengths of tarsomeres 1–3. Claws black.

***Wings*** (Fig. 25A). Without markings. Membrane completely transparent, sometimes shaded with light brown. Veins and crossveins mostly dark brown. Forewings presectoral area with 6–9 crossveins; Cu with six or seven rows of cells; pterostigma dark brown. Hindwings shorter and narrower than forewings; presectoral area with 6–8 crossveins; Cu with five or six rows of cells; pterostigma dark brown.

***Abdomen*** (Fig. 25A). Shorter than hindwing, reddish brown, covered with sparse black setae.

***Genitalia*** (Fig. 25D, E, H–K). Ectoproct elongated, forcipate, covered with long black setae. Sternite IX broad, covered with long black setae. Gonarcus brown, triangular, with short lateral arm. Parameres well sclerotized, dark brown, strongly raised in lateral view. Pulvinus symmetrical, elongated, digitiform, attached to gonarcus, covered with long black setae.

Size. BL: 31.1–34.6 mm; FWL: 32.6–37.3 mm; HWL: 29.4–33.9 mm

**Female, adult.** Except terminalia, generally similar to male. Terminalia (Fig. 25F, G): tergite IX narrow, triangular in lateral view; ectoproct triangular in lateral view; distivalvae semicircular in lateral view, smaller than ectoproct; ventrovalvae triangular in ventral view; interdens distinct.

Size. BL: 30.1–34.4 mm; FWL: 34.9–39.1 mm; HWL: 30.2–34.8 mm.

**Larva, 3^rd^ instar.** General color yellowish brown, with dark brown markings (Fig. 26A–C). Head quadrate, approximately as long as broad; dorsal side of the head capsule brown with some yellow markings; mandibles brown, covered with short black setae; interdental pseudo teeth (3–4) (3–4) (1) (Fig. 26D, E). Dorsal side of the abdominal tergites with a dark brown median longitudinal stripe (Fig. 26A). Abdominal sternite VIII with a pair of brown spots in correspondence of the odontoid processes; abdominal sternite IX triangular, with yellow marking on anterior margin (Fig. 26F).

Size. BL: 17.0 mm; HL: 3.7 mm, HW: 3.6 mm, ML: 4.2 mm.

##### Biological notes.

*Ascalohybris subjacens* is commonly observed throughout the country in various habitats such as grasslands, mountainous regions, and coastal areas (Fig. 37G). Adults emerge from July to September in South Korea. They are nocturnal and can be observed flying actively or resting on grass stems at night (Fig. 33B). More than 40 eggs are laid on substrates like dry grass stems in grasslands. At the oviposition site, hatched larvae can be observed either clustered together or scattered nearby (Fig. 34B). Larvae are ambush hunters and a single specimen was collected from under a rock on a rocky hill (Fig. 36H).

##### Distribution.

Korea, China, Japan, Vietnam, Cambodia (Wang et al. 2018).

##### Remarks.

Okamoto (1924) identified and reported specimens of *Ascalohybris subjacens* from Jeju Island. Okamoto (1926) described this species as common in southern Korea. Indeed, it is a representative owlfly species commonly observed throughout South Korea.

#### ﻿Genus Libelloides

Taxon classificationAnimaliaNeuropteraMyrmeleontidae

Schäffer, 1763

F977D799-E714-5B8D-9FC9-4446B4D78E93

Libelloides Schäffer, 1763: 1. Type species: Papilio coccajus Denis & Schiffermüer, 1775. Type locality: Austria.
Ascalaphus Fabricius, 1775: 313.

##### Diagnosis.

Adult. Wings conspicuously colored with numerous black, yellow, and white markings; triangular, short, and broad; forewing vein CuA2 runs nearly parallel to CuP to the wing margin; abdomen short and stout; male ectoproct elongated, forming a distinct ectoproct (Wang et al. 2018). Third instar Larva. Mandibles with three teeth, the median tooth is the largest and closer to the apical tooth than to basal tooth; mandibles with interdental pseudo-teeth; abdomen with eight pairs of dorsal cylindrical scolus-like processes; sternite VIII with short odontoid processes; sternite IX with two short rastra each four digging setae (Fig. 3E) (Badano and Pantaleoni 2014b).

##### Distribution.

Palaearctic region.

#### ﻿ Libelloides sibiricus

Taxon classificationAnimaliaNeuropteraMyrmeleontidae

(Eversmann, 1850)

224871AD-8860-52FD-A6D2-0D7144BFC811

Figs 27, 28, 29B, 33A, 34A, 35J, 36H, 37G

Ascalaphus sibiricus Eversmann, 1850: 279. Type locality: Russia: eastern Siberia: near Kyakhta
Ascalaphus radians Gerstaecker, 1885: 8. Type locality: Russia: Amur.
Ascalaphus sibiricus var. niveus Navás, 1929: 33. Type locality: Russia: “Borochojewa, Transbaikal”.
Libelloides sibiricus (Eversmann, 1850): Tjeder 1972: 153.

##### Specimens examined.

[**JBNU**] • 2♂, Seolgye-ri, Yeongdong-eup, Yeongdong-gun, Chungcheongbuk-do, Korea, 17.IV.2021, J.S. Kim; • 1♂3♀, Changwon-ri, Nam-myeon, Yeongwol-gun, Gangwon-do, Korea, 18.V.2024, M.K. Jeong; 1 larva (3^rd^ instar), Seolgye-ri, Yeongdong-eup, Yeongdong-gun, Chungcheongbuk-do, Korea, 21.II.2022, J.S. Kim; • 2 larvae (1^st^ instar), Yulji-ri, Susan-myeon, Jecheon-si, Chungcheongbuk-do, 25.V.2025, J.S. Kim.

##### Diagnosis.

Frons is black and densely covered with long yellowish brown hairs. Pronotum is narrow, considerably shorter than its width, black, with transverse yellow stripe. Hindwing is pale brown at the distal part. Hindwing has dark brown stripes along crossveins M and CuP, and the area between them is yellow. In larvae, head capsule is dark brown with some yellow markings and has a darker anterior portion. On the dorsal side, each abdominal tergite has a dark brown V-shaped marking, and a pair of setal tufts is located along it.

##### Description.

**Figure 27.**
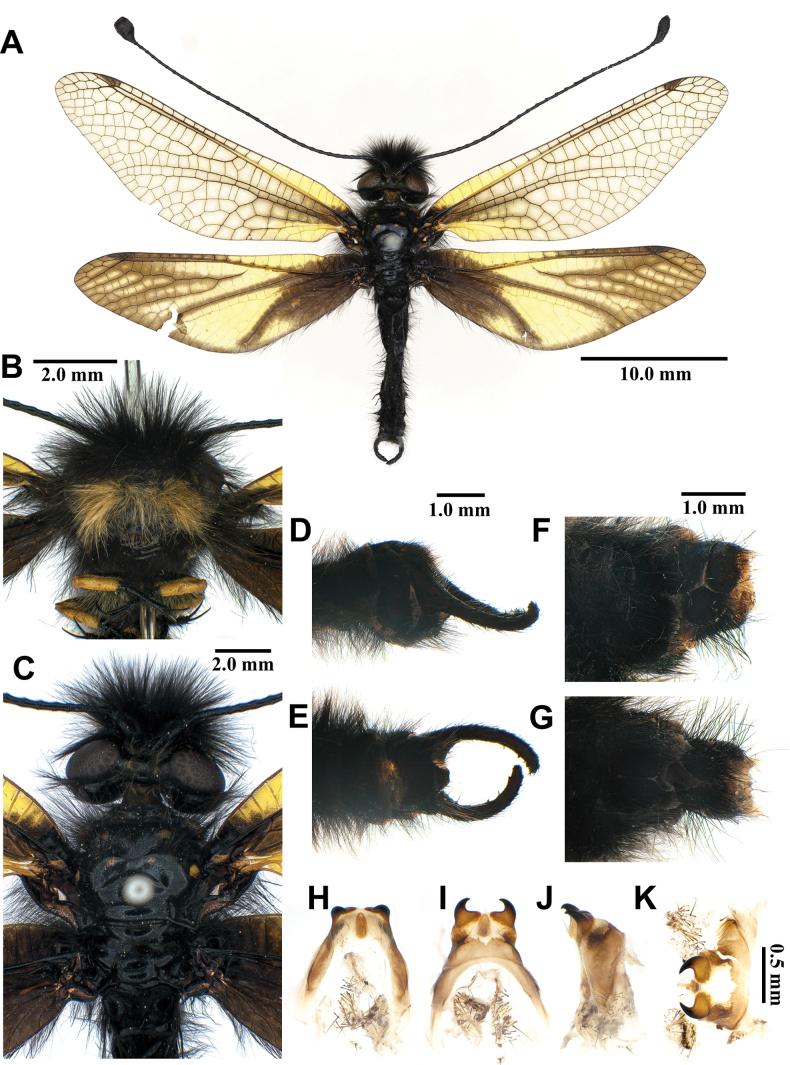
*Libelloides sibiricus* (Eversmann, 1850), adult. A. Dorsal habitus, male; B. Head, frontal view; C. Head and thorax, dorsal view; D, E. Male terminalia: D. Lateral view; E. Ventral view; F, G. Female terminalia: F. Lateral view; G. Ventral view; H–K. Male genitalia: H. Dorsal view; I. Ventral view; J. Lateral view; K. Caudal view.

**Male, adult. *Head*** (Fig. 27B, C). Vertex slightly narrow, slightly depressed, black, densely covered with long black hairs. Frons black, densely covered with long yellowish brown hairs; clypeus black, moderately covered with brown hairs. Eye with a transverse furrow. Antenna dark brown, considerably long, with strongly defined club; flagellum comprising ~40 flagellomeres. Mouthparts black; labrum black, with brown hairs; maxillary palpus black; labial palpus black.

***Thorax*** (Fig. 27B). Pronotum narrow, considerably shorter than width, black, with transverse yellow stripe, moderately covered with long black hairs. Mesonotum and metanotum generally black, with some small yellow markings; moderately covered with long black hairs.

***Wing*** (Fig. 27A). With yellow and dark brown markings. Forewings membrane mostly transparent, basal quarter yellow; veins and crossveins mostly brown; presectoral area with 6–9 crossveins; Cu with five or six rows of cells; pterostigma dark brown. Hindwings shorter and broader than forewings; membrane shaded with yellow and dark brown; veins and crossveins dark brown and yellowish white; presectoral area with 5–8 crossveins and some cells; Cu with five or six rows of cells; pterostigma dark brown.

***Legs*.** Coxae black, densely covered with long black hairs. Femora generally black, distally yellow, moderately covered with long black and yellowish white hairs. Tibiae generally yellow, distally black, covered with sparse black setae. Tibial spurs black, short, slightly curved, approximately as long as tarsomere 1. Tarsi black, tarsomere 5 approximately as long as combined lengths of tarsomeres 1–4. Claws black.

***Abdomen*** (Fig. 27A). Shorter than hindwing, black, densely covered with long black hairs.

***Genitalia*** (Fig. 27D, E, H–K). Ectoproct elongated, forcipate, covered with long black setae. Sternite IX broad, covered with long black setae. Gonarcus brown, arched, with lateral arm. Mediuncus brown, oval in dorsal view. Parameres well sclerotized, dark brown, strongly hooked in lateral view. Pulvinus symmetrical, attached to gonarcus, covered with long black setae.

Size. BL: 19.8–24.4 mm; FWL: 19.4–24.1 mm; HWL: 17.6–20.2 mm.

**Female, adult.** Except terminalia, generally similar to male. Terminalia (Fig. 27F, G): tergite IX narrow, rectangular in lateral view; ectoproct rectangular in lateral view; distivalvae circular in lateral view, smaller than ectoproct; ventrovalvae rectangular in ventral view; interdens absent.

Size. BL: 20.3–23.4 mm; FWL: 27.4–29.5 mm; HWL: 23.3–26.0 mm.

**Figure 28.**
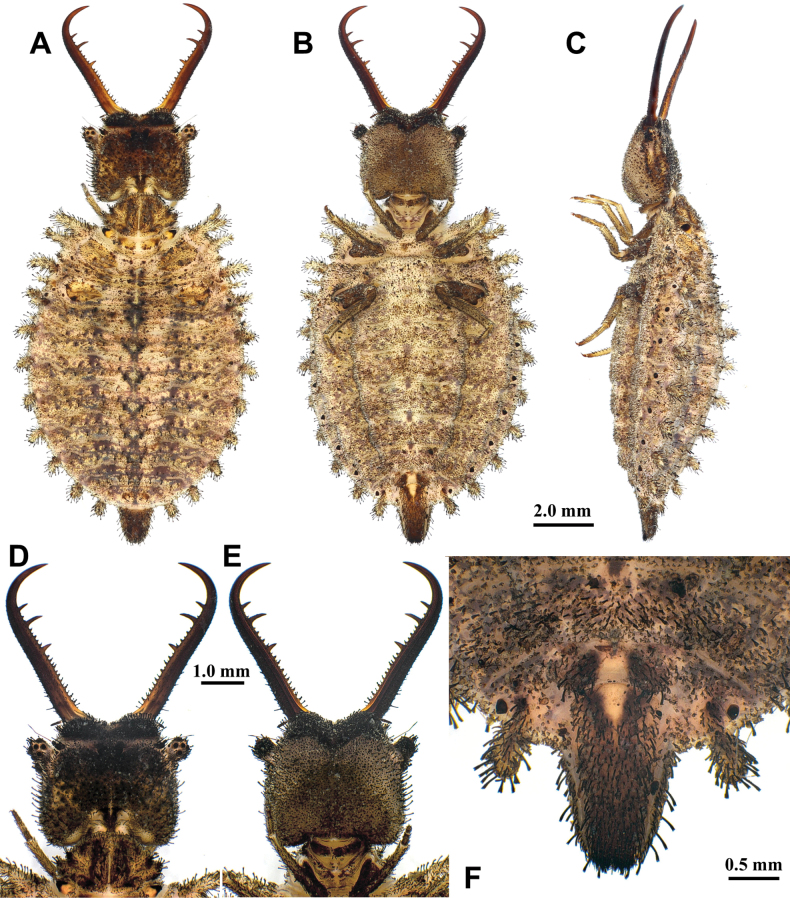
*Libelloides sibiricus* (Eversmann, 1850), third instar larva. A–C. Habitus: A. Dorsal view; B. Ventral view; C. Lateral view; D, E. Head: D. Dorsal view; E. Ventral view; F. Abdominal sternite IX.

**Larva, 3^rd^ instar.** General color yellowish brown, with dark brown markings (Fig. 28A–C). Head quadrate, shorter than wide; dorsal side of the head capsule dark brown with some yellow markings, with a darker anterior portion; mandibles dark brown, covered with short black setae; interdental pseudo teeth (6–7) (2–3) (1) (Fig. 28D, E). Each abdominal tergite with a dark brown V-shaped marking on the dorsal side; a pair of setal tufts located along the marking (Fig. 28A). Abdominal sternite VIII with a pair of brown spots in correspondence of the odontoid processes; abdominal sternite IX triangular, with yellow marking on anterior margin (Fig. 28F).

Size. BL: 14.3 mm; HL: 2.9 mm, HW: 3.2 mm, ML: 3.7 mm.

##### Biological notes.

**Figure 29.**
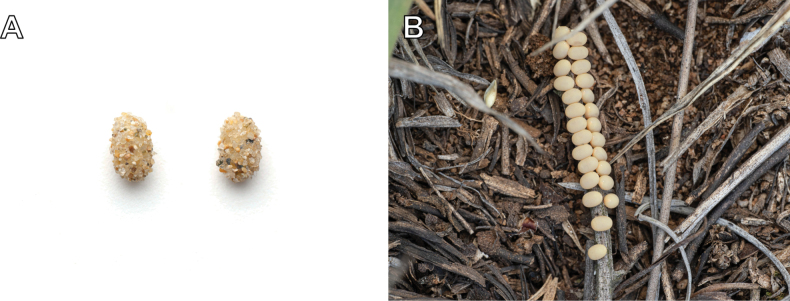
Photographs of eggs, A. *Synclisis japonica*; B. *Libelloides sibiricus*.

*Libelloides sibiricus* inhabits inland grasslands and is particularly common on rocky hills and in calcareous grasslands (Fig. 37G). Adults mainly emerge from April to May in South Korea. They are diurnal and can be observed flying actively or resting on grass stems during the daytime (Fig. 33A). More than 20 eggs are laid on substrates like dry grass stems in grasslands (Fig. 29B). At the oviposition site, hatched larvae can be found clustered together or scattered nearby (Fig. 34A). The larvae are ambush hunters with only one specimen collected from under a rock on a rocky hill (Fig. 36H).

**Figure 30.**
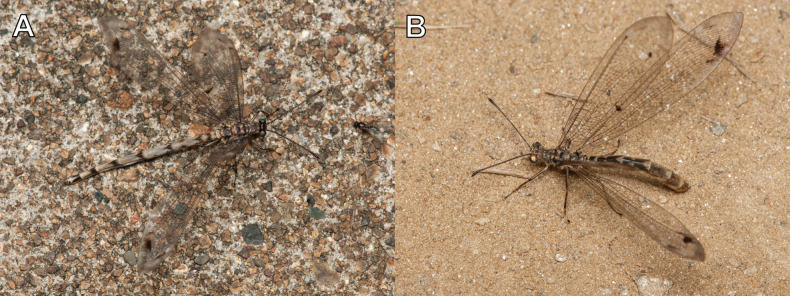
Photographs of *Paraglenurus albiventris* in the field, A. Male; B. Female.

**Figure 31.**
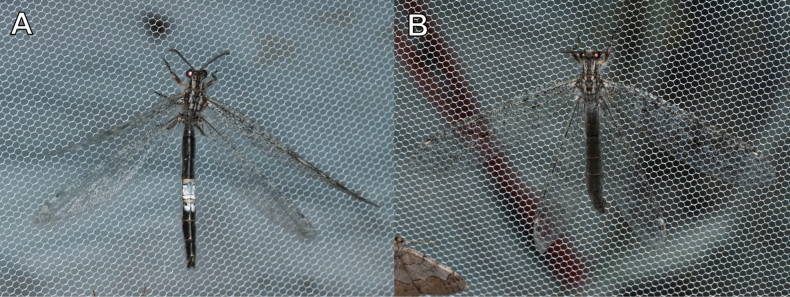
Photographs of *Synclisis japonica* in the field, A. Male; B. Female.

##### Distribution.

Korea, China, Russia (Wang et al. 2018).

##### Remarks.

van der Weele (1909) reported specimens collected from Wonsan-si (Gangwon-do, North Korea) identified as *Libelloides sibiricus*. Based on morphological differences between specimens from China, he also described *Libelloides sibiricus chinensis* (van der Weele, 1909) as a new subspecies. A variation in which the yellow parts of the wings are white is known from Russia (van der Weele, 1909), but this variation has not been observed in Korea.

**Figure 32.**
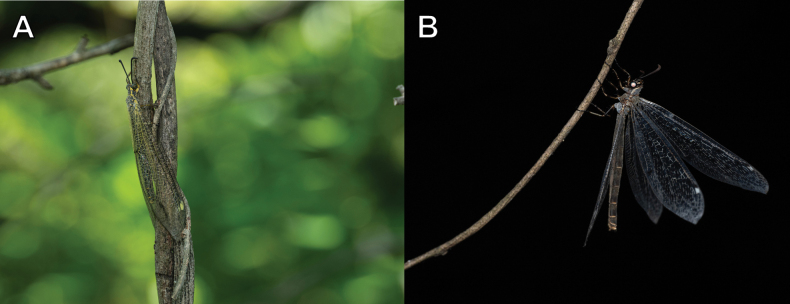
Photographs of adults in the field, A. *Deutoleon lineatus lineatus*; B. *Euroleon coreanus*.

**Figure 33.**
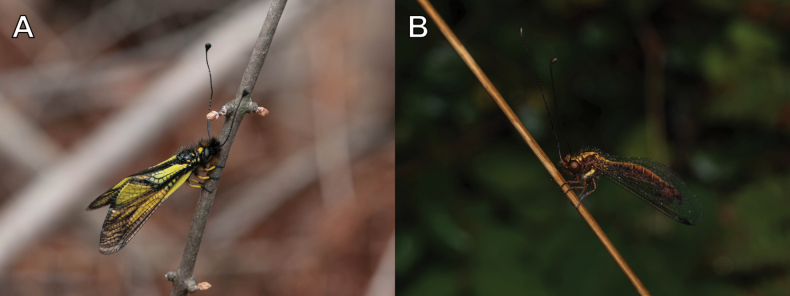
Photographs of adults in the field, A. *Libelloides sibiricus*; B. *Ascalohybris subjacens*.

**Figure 34.**
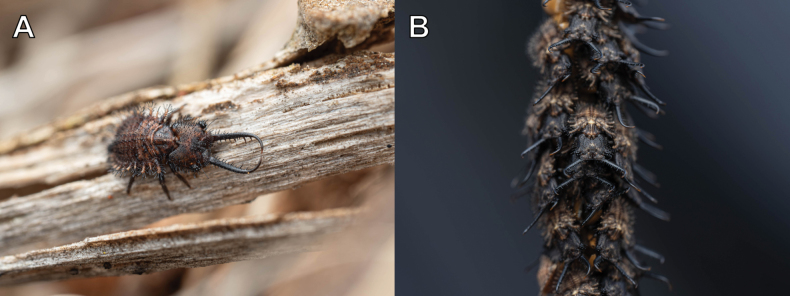
Photographs of first instar larvae in the field, A. *Libelloides sibiricus*; B. *Ascalohybris subjacens*.

**Figure 35.**
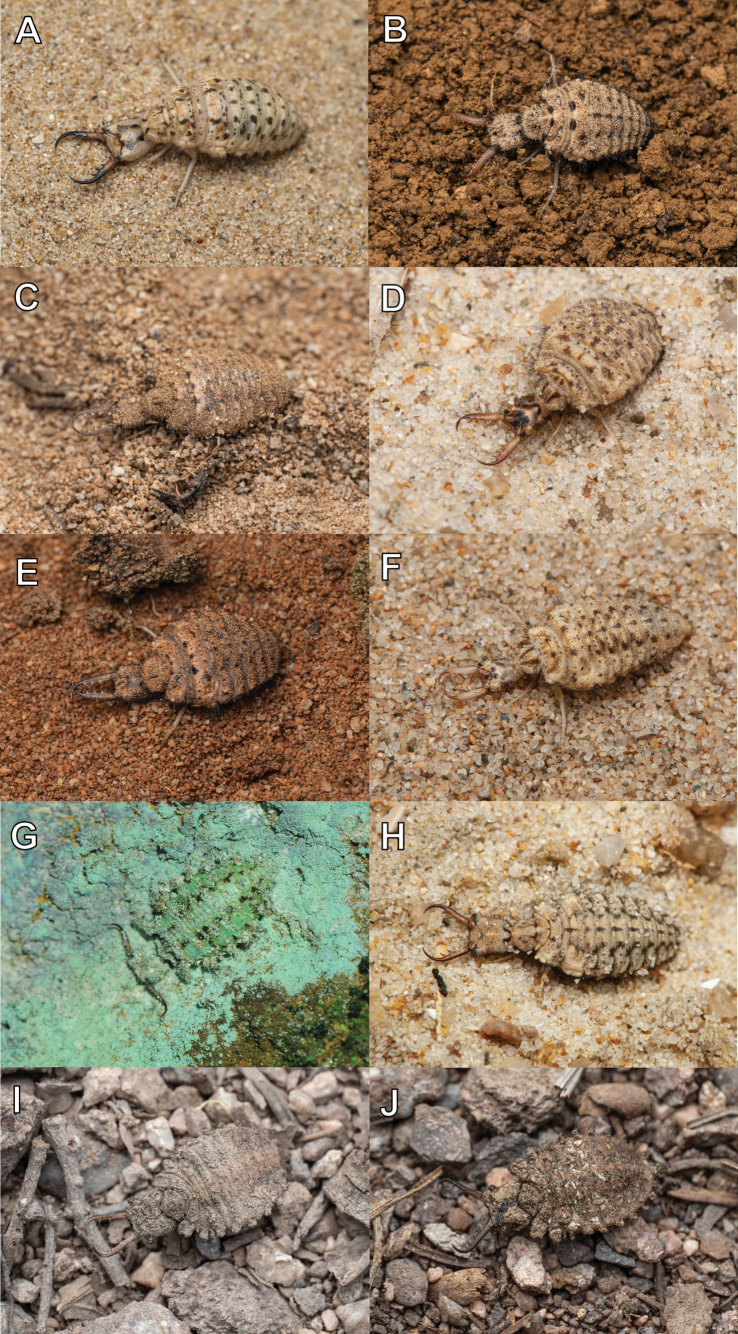
Living third instar larvae. A. *Synclisis japonica*; B. *Baliga micans*; C. *Euroleon coreanus*; D. *Myrmeleon bore*; E. *Myrmeleon formicarius*; F. *Myrmeleon immanis*; G. *Nepsalus jezoensis*; H. *Distoleon littoralis*; I. *Ascalohybris subjacens*; J. *Libelloides sibiricus*.

**Figure 36.**
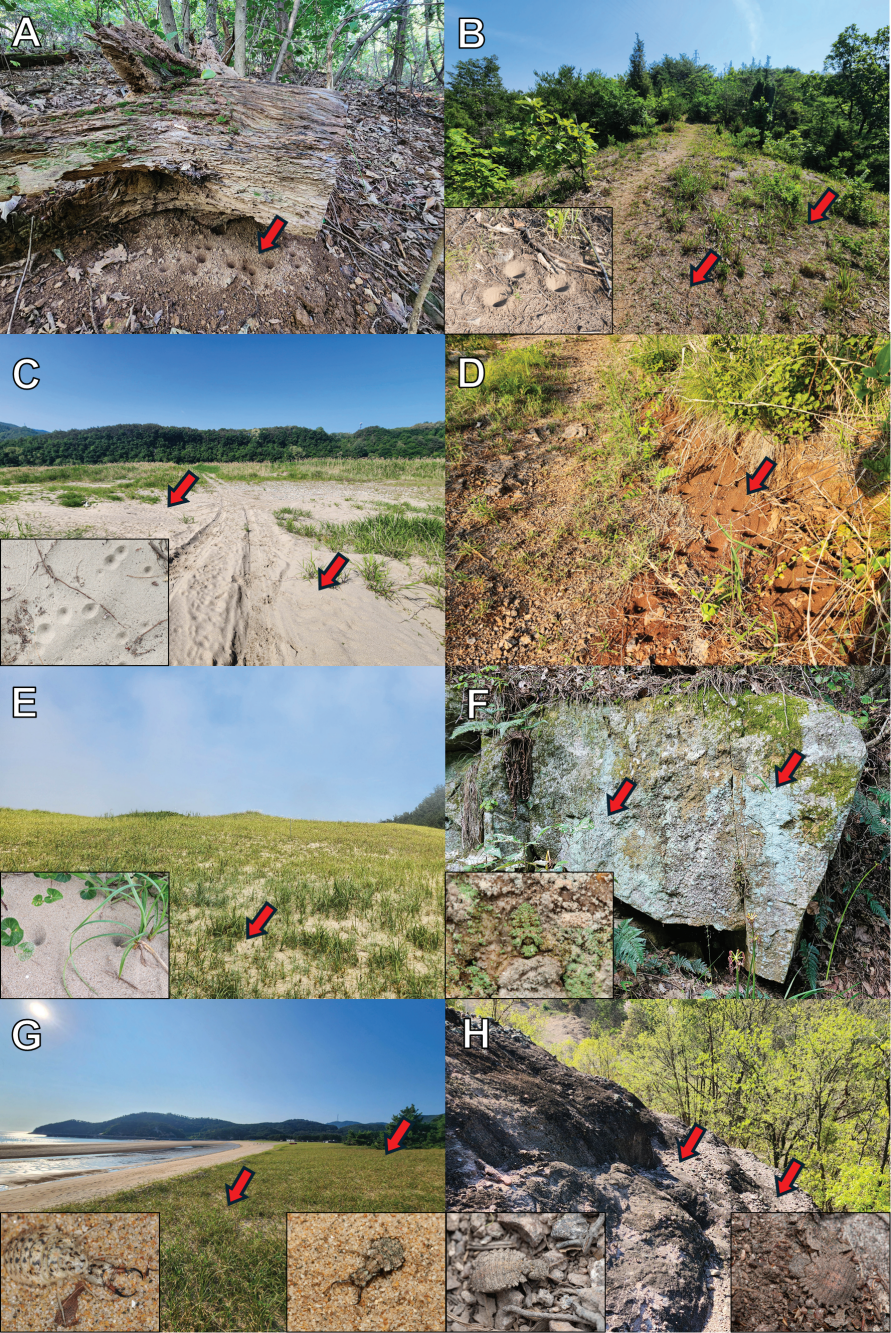
Habitats of the Myrmeleontidae larvae, with arrows indicating the positions where the larvae were collected. A. Under a dead tree trunk (*Baliga micans*); B. Calcareous grassland in limestone area (*Euroleon coreanus*); C. Sandy riverbank (*Myrmeleon bore*); D. Cut slope beside trail (*Myrmeleon formicarius*); E. Foredune (*Myrmeleon immanis*); F. Rock wall covered with lichen (*Nepsalus jezoensis*); G. Natural coastal dune (*Synclisis japonica* and *Distoleon littoralis*); H. Rocky hill (*Ascalohybris subjacens* and *Libelloides sibiricus*).

### ﻿Key to species of Myrmeleontidae in Korea

**Table:** 

1	Antennae < 1/3 of body length. Eye entire, without transverse furrow	**2**
–	Antennae > 1/2 of body length. Eye divided by a transverse furrow into upper and lower portions. (tribe Ascalaphini)	**15**
2	Larger in size, forewing length ~49–59 mm. Marginal pronotum and legs with dense hairs. Male tergite V and proximal 1/2 of tergite VI covered with silver pubescens. (tribe Acanthaclisini)	***Synclisis japonica* (Hagen)**
–	Smaller in size, forewing length ~22–44 mm. Marginal pronotum and legs without dense hairs. Male tergites V and VI without silver pubescens	**3**
3	Forewing veins 2A and 3A separate. (tribe Dendroleontini)	**4**
–	Forewing veins 2A and 3A fused	**5**
4	Larger in size, forewing length ~35–37 mm. Hindwing with large brown marking extending from proximal part of pterostigma to posterior margin	***Dendroleon pupillaris* Gerstaecker**
–	Smaller in size, forewing length ~23–31 mm. Hindwing with small brown markings along posterior margin	***Nepsalus jezoensis* Okamoto**
5	Hindwing presectoral area with ≥ 4 crossveins. (tribe Myrmeleontini)	**6**
–	Hindwing presectoral area with 1 or 2 crossveins	**10**
6	Forewing with distinct dark brown markings; forewing vein CuA2 and CuP+1A generally parallel	***Euroleon coreanus* Okamoto**
–	Forewing without any marking; forewing vein CuA2 and CuP+1A converging toward wing margin	**7**
7	Antenna approximately as long as length of head plus thorax. Hind coxae pale yellow. Female anterior gonapophyses much longer than wide, digitiform. Abdominal sternite IX of larva with 0–2 short digging setae in front of rastra	***Baliga micans* (McLachlan)**
–	Antenna shorter than length of head plus thorax. Hind coxae dark brown. Female anterior gonapophyses wider than long, tuberculate. Abdominal sternite IX of larva with ≥ 3 short digging setae in front of rastra. (genus *Myrmeleon*)	**8**
8	Clypeus without dark brown marking. Wing veins and crossveins mostly pale yellow. Abdominal sternite IX of larva with ~10 dense short digging setae in front of rastra	***Myrmeleon immanis* Walker**
–	Clypeus with dark brown marking. Wing veins and crossveins mostly dark brown. Abdominal sternite IX of larva with only four short digging setae in front of rastra	**9**
9	Wing veins MA mostly pale yellow. Male without pilula axillaris. Larval hind coxa with some dark markings	***Myrmeleon formicarius* (Linnaeus)**
–	Wing veins MA mostly dark brown. Male with pilula axillaris. Larval hind coxa without markings	***Myrmeleon bore* Tjeder**
10	Antenna approximately as long as length of head plus thorax. Eye small, narrower than frons. Leg thick, hind femur plus tibia shorter than length of head plus thorax; claw not opposable. Male genitalia with a forked paramere. (tribe Nemoleontini)	**11**
–	Antenna longer than length of head plus thorax. Eye big, as wide as frons. Leg slender, hind femur plus tibia approximately as long as length of head plus thorax; claw opposable. Male genitalia with a pair of plate-like paramere. (tribe Megistopini, genus *Paraglenurus*)	**13**
11	Hindwing presectoral area with 2 crossveins	***Deutoleon lineatus lineatus* (Fabricius)**
–	Hindwing presectoral area with only 1 crossvein	**12**
12	3^rd^ labial palpomere dark brown. Hindwing rhegma area with distinct large dark brown marking	***Distoleon nigricans* (Okamoto)**
–	3^rd^ labial palpomere yellowish brown. Hindwing rhegma area without distinct large dark brown marking	***Distoleon littoralis* Miller & Stange**
13	Abdominal tergites III–V dark brown, each with pair of median pale spots. Forewing with white marking along posterior margin	**14**
–	Abdominal tergites II–V largely yellowish white in male. Forewing without white marking along posterior margin	***Paraglenurus albiventris* Matsumoto, Kikuta & Hayashi**
14	Antenna apical ~1/4, each flagellum with apical 1/3–1/2 pale yellow. Hindwing with a distinct preapical dark brown marking and a distinct and rounded adjacent white marking	***Paraglenurus melanostictus* Matsumoto, Kikuta & Hayashi**
–	Antenna apical ~1/4, each flagellum with apex only slightly pale yellow. Hindwing with a distinct preapical dark brown marking and an indistinct and oval-shaped adjacent white marking	***Paraglenurus japonicus* (McLachlan)**
15	Wings with yellow and dark brown color. Abdomen with long black hairs. Abdominal tergite of larva with a dark brown median longitudinal stripe on the dorsal side	***Libelloides sibiricus* (Eversmann)**
–	Wings without yellow and dark brown color. Abdomen without long black hair. Abdominal tergite of larva with a dark brown V-shaped marking on the dorsal side	***Ascalohybris subjacens* (Walker)**

## ﻿Discussion

Within the family Myrmeleontidae, some species have specialized larval niche requirements that lead to restricted distributions (Stange and Miller 1990; Stange et al. 2003), while others are known to be generalists with wider habitat ranges (Mansell and Erasmus 2002; Hévin et al. 2023; Zheng et al. 2024b; Ascenzi et al. 2025). Antlions in Korea exemplify these characteristics, with many habitat types observed, ranging from euryoecious species like *Paraglenurus japonicus*, widely distributed from mountainous to coastal areas, to specialists such as *Dendroleon pupillaris* in high mountains, *Myrmeleon bore* in sandy terrains from riverbanks to coastal dunes, and *Nepsalus jezoensis*, whose larvae inhabit lichen-covered rocks. Based on our research, the habitat types of Korean antlion species can be broadly classified as forest-dwelling, coastal, and grassland-dwelling. Forest-dwelling species like *Baliga micans* and *Paraglenurus melanostictus* were found to be relative generalists with wider distributions, whereas the coastal and grassland categories included multiple species with restricted distributions dependent upon specific environmental conditions.

**Figure 37.**
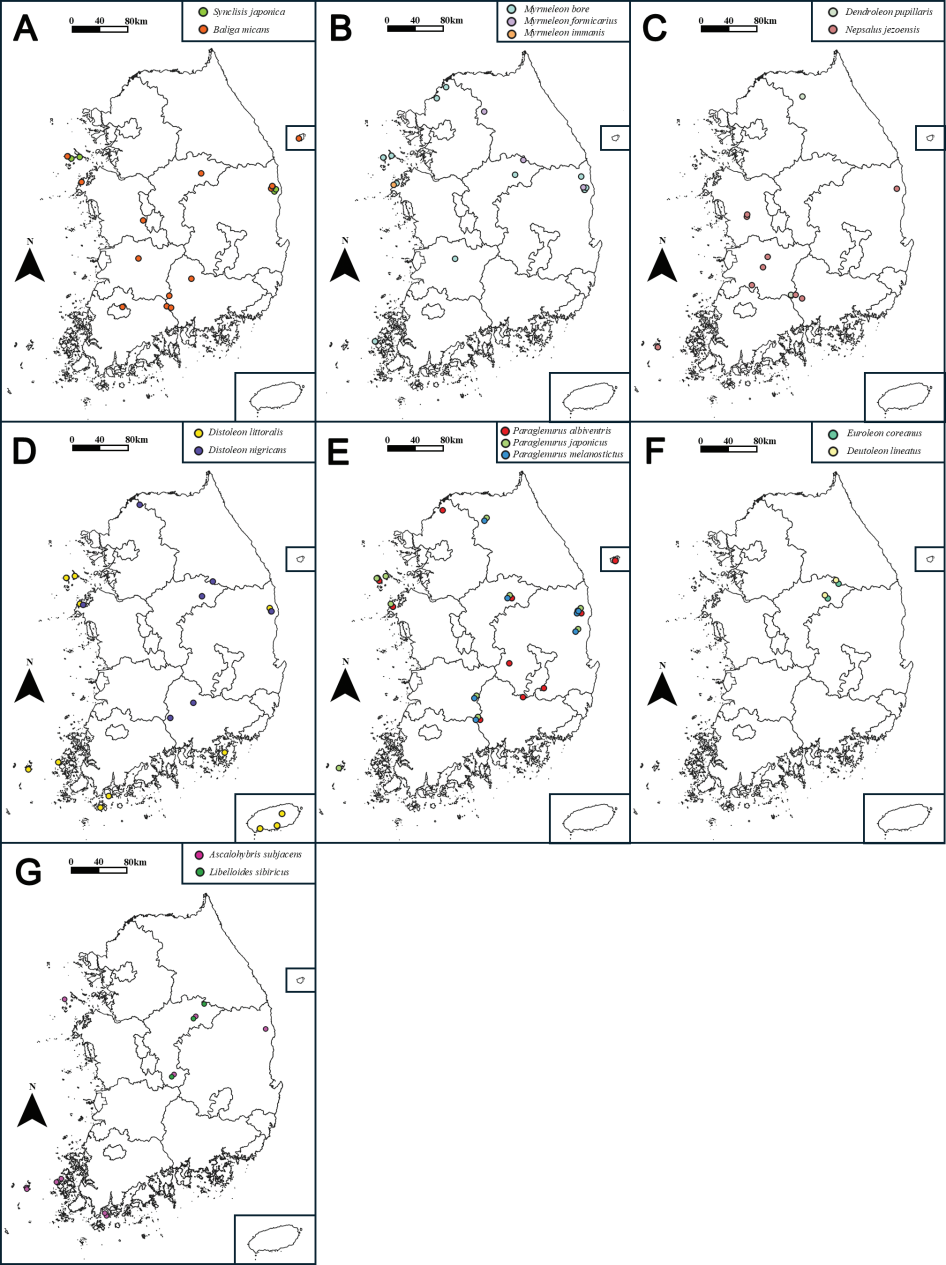
Distribution maps. A. *Synclisis japonica* and *Baliga micans*; B. Genus *Myrmeleon* (*Myrmeleon bore*, *Myrmeleon fomicarius*, and *Myrmeleon immanis*); C. *Dendroleon pupillaris* and *Nepsalus jezoensis*; D. *Genus Distoleon* (*Distoleon littoralis* and *Distoleon nigricans*); E. Genus *Paraglenurus* (*Paraglenurus albiventris*, *Paraglenurus japonicus*, and *Paraglenurus melanostictus*); F. *Euroleon coreanus* and *Deutoleon lineatus lineatus*; H. *Ascalohybris subjacens* and *Libelloides sibiricus*.

**Figure 38.**
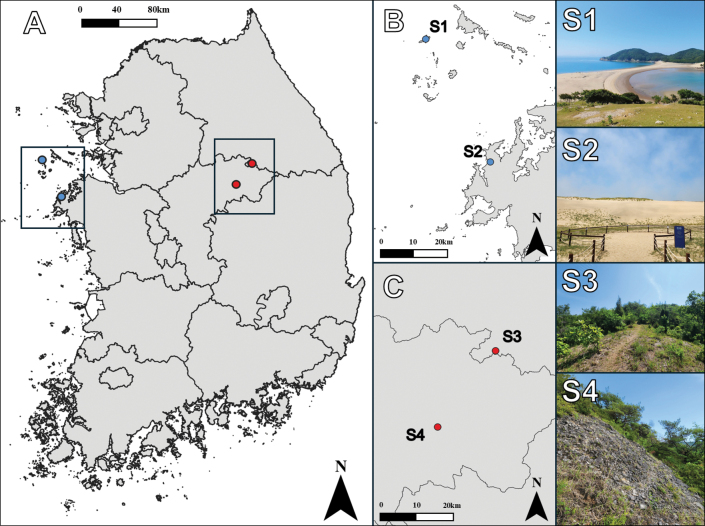
Areas for the conservation of Myrmeleontidae in Korea. A. Map showing the locations of the most noteworthy; blue dots indicate coastal sites, and red dots indicate grassland sites. B. Detailed map of the coastal sites. C. Detailed map of the grassland sites. S1. Gureom-ri (Ongjin-gun, Incheon); S2. Sindu-ri (Taean-gun, Chungcheongnam-do); S3. Changwon-ri (Yeongwol-gun, Gangwon-do); S4. Yulji-ri (Jecheon-si, Chungcheongbuk-do).

In Japan, *Synclisis japonica* and *Myrmeleon solers* have been reported as restricted to natural coastal dunes (Matsumoto et al. 2016). In this study, we confirmed that *Synclisis japonica* and *Myrmeleon immanis* also have restricted distributions in well-preserved coastal dunes in Korea (Fig. 37A, B). This is significant in that coastal ecosystems around the world are rapidly disappearing due to rising sea levels, coastal development, and the influx of invasive species (He and Silliman 2019; Rippel et al. 2021). The case of another Korean coastal insect illustrates how urgent this threat is for antlions: the tiger beetle *Abroscelis anchoralis* (Chevrolat, 1845), an endangered coastal insect, was historically found locally along the west coast of Korea but is now confined to extremely narrow habitat ranges in Taean-gun (Chungcheognam-do) and Sinan-gun (Jeollanam-do). Consequently, this species is designated as a Class I Endangered Wildlife and listed as Critically Endangered (CR) on the Korean Red List (National Institute of Biological Resources 2023; Lee et al. 2025). This drastic population decline is presumably caused primarily by the destruction of coastal dunes by development as well as sea level rises, suggesting that Korean coastal antlions face similar threats. The west coast of Korea, from Chungcheongnam-do to Incheon, represented by areas like Gureom-ri (Ongjin-gun, Incheon) and Sindu-ri (Taean-gun, Chungcheongnam-do), feature well-developed coastal dunes (Fig. 38A, B). These areas were found to support abundant populations of coastal antlion species, including not only *Synclisis japonica* and *Myrmeleon immanis* but also *M. bore* and *Distoleon littoralis* (Table 1). Therefore, these regions are of high value as core areas for the conservation of coastal antlions and will need legal protection.

**Table 1.** List of Myrmeleontidae collected at each site.

Site	Species
Gureom-ri (Ongjin-gun, Inchoen)	*Synclisis japonica*, *Baliga micans*, *Myrmeleon bore*, *Distoleon littoralis*, *Paraglenurus albiventris*, *Paraglenurus japonicus*, *Ascalohybris subjacens*
Sindu-ri (Taean-gun, Chungcheongnam-do)	*Baliga micans*, *Myrmeleon bore*, *Myrmeleon immanis*, *Distoleon littoralis*, *Distoleon nigricans*, *Paraglenurus albiventris*, *Paraglenurus japonicus*
Changwon-ri (Yeongwol-gun, Gangwon-do)	*Baliga micans*, *Euroleon coreanus*, *Myrmeleon formicarius*, *Deutoleon lineatus lineatus*, *Distoleon nigricans*, *Libelloides sibiricus*
Yulji-ri (Jecheon-si, Chungcheongbuk-do)	*Baliga micans*, *Euroleon coreanus*, *Myrmeleon bore*, *Myrmeleon formicarius*, *Deutoleon lineatus lineatus*, *Distoleon nigricans*, *Paraglenurus albiventris*, *Paraglenurus japonicus*, *Paraglenurus melanostictus*, *Ascalohybris subjacens*, *Libelloides sibiricus*

Our study confirmed that the antlions *Euroleon coreanus* and *Deutoleon lineatus lineatus* are restricted to calcareous grasslands in limestone regions such as Jecheon-si (Chungcheongbuk-dp) and Yeongwol-gun (Gangwon-do) (Fig. 37F), presenting a stark contrast with historical records. Okamoto (1926) recorded these species in Seoul and Suwon-si (Gyeonggi-do), where they are no longer found, and even referred to *E. coreanus* as a ‘common species’ at the time, indicating that the distribution of these species has significantly contracted.

This pattern of range contraction is mirrored in other insect groups. The nymphalid butterflies *Melitaea scotosia* Butler, 1878 and *Euphydryas sibirica* (Staudinger, 1861), formerly distributed in Gyeonggi-do, are now found only in some grasslands of Jecheon-si and Yeongwol-gun, similar to the antlions (Joo et al. 2021; National Institute of Biological Resources 2022). As a result, these butterflies are now listed as Endangered (EN) and Vulnerable (VU) on the Korean Red List. Calcareous grasslands are thus serving as a final refuge for insects that once had much wider distributions.

Semi-natural grasslands, maintained by traditional agricultural practices, have long supported high biodiversity (Tilman et al. 2001; Benton et al. 2003; Kleijn et al. 2011). However, the shift to modern land-use has led to the loss of these habitats, causing declines in many endangered insect populations (Louto et al. 2003; Tscharntke et al. 2005; Uematsu et al. 2010; Uchida and Ushimaru 2014). In this context, calcareous grasslands are considered critically important habitats for insect conservation (Van Swaay 2002; Wenzel et al. 2006). The calcareous grasslands from southern Gangwon-do to northern Chungcheongbuk-do, represented by areas like Changwon-ri (Yeongwol-gun) and Yulji-ri (Jecheon-si) that support abundant populations of *Euroleon coreanus*, *Deutoleon lineatus lineatus*, *Myrmeleon formicarius*, and *Libelloides sibiricus*, can be considered core conservation areas (Fig. 38A, C; Table 1).

Antlion larvae occupy well-defined ecological niches to avoid interspecific competition, and their distribution and behavior are influenced by specific factors of habitats. This ecological sensitivity means they are differentially affected by human interference and environmental changes (Ascenzi et al. 2025). The dependence of Korean antlions on specific habitats like coastal dunes and calcareous grasslands, combined with the trend of range contraction due to habitat destruction, demonstrates that they can serve as valuable bioindicators for assessing the impacts of habitat alteration. Therefore, the conservation of antlions is not merely about protecting individual species but can also serve as a crucial milestone for understanding alterations in the ecosystem.

## Supplementary Material

XML Treatment for Synclisis

XML Treatment for Synclisis japonica

XML Treatment for Baliga

XML Treatment for Baliga micans

XML Treatment for Euroleon

XML Treatment for Euroleon coreanus

XML Treatment for Myrmeleon

XML Treatment for Myrmeleon bore

XML Treatment for Myrmeleon formicarius

XML Treatment for Myrmeleon immanis

XML Treatment for Dendroleon

XML Treatment for Dendroleon pupillaris

XML Treatment for Nepsalus

XML Treatment for Nepsalus jezoensis

XML Treatment for Deutoleon

XML Treatment for Deutoleon lineatus lineatus

XML Treatment for Distoleon

XML Treatment for Distoleon littoralis

XML Treatment for Distoleon nigricans

XML Treatment for Paraglenurus

XML Treatment for Paraglenurus albiventris

XML Treatment for Paraglenurus japonicus

XML Treatment for Paraglenurus melanostictus

XML Treatment for Ascalohybris

XML Treatment for Ascalohybris subjacens

XML Treatment for Libelloides

XML Treatment for Libelloides sibiricus

## ﻿Additional information

### Use of AI

No use of AI was reported.

### Author ORCIDs

Jiseung Kim https://orcid.org/0009-0004-8986-4362

Neung-Ho Ahn https://orcid.org/0009-0006-7859-068X

Sora Kim https://orcid.org/0000-0003-4402-5421

### Data availability

All of the data that support the findings of this study are available in the main text.
